# Sequence of a Complete Chicken BG Haplotype Shows Dynamic Expansion and Contraction of Two Gene Lineages with Particular Expression Patterns

**DOI:** 10.1371/journal.pgen.1004417

**Published:** 2014-06-05

**Authors:** Jan Salomonsen, John A. Chattaway, Andrew C. Y. Chan, Aimée Parker, Samuel Huguet, Denise A. Marston, Sally L. Rogers, Zhiguang Wu, Adrian L. Smith, Karen Staines, Colin Butter, Patricia Riegert, Olli Vainio, Line Nielsen, Bernd Kaspers, Darren K. Griffin, Fengtang Yang, Rima Zoorob, Francois Guillemot, Charles Auffray, Stephan Beck, Karsten Skjødt, Jim Kaufman

**Affiliations:** 1 Basel Institute for Immunology, Basel, Switzerland; 2 Department of Veterinary Disease Biology, University of Copenhagen, Copenhagen, Denmark; 3 Department of International Health, Immunology and Microbiology, University of Copenhagen, Copenhagen, Denmark; 4 Department of Pathology, University of Cambridge, Cambridge, United Kingdom; 5 Pirbright Institute (formerly Institute for Animal Health), Compton, United Kingdom; 6 Department of Zoology, Oxford University, Oxford, United Kingdom; 7 Department of Medical Microbiology, University of Oulu and Nordlab, Oulu, Finland; 8 Institute for Animal Physiology, Department of Veterinary Sciences, Ludwig Maximilians University, Munich, Germany; 9 School of Biosciences, University of Kent, Canterbury, United Kingdom; 10 Wellcome Trust Sanger Institute, Hinxton, United Kingdom; 11 Institute for Cellular and Molecular Embryology, CNRS UMR 7128, Nogent-sur-Marne, France; 12 Institute Andre Lwoff, CNRS FRE 2937, Villejuif, France; 13 Department of Cancer and Inflammation, University of South Denmark, Odense, Denmark; 14 Department of Veterinary Medicine, University of Cambridge, Cambridge, United Kingdom; Harvard University, United States of America

## Abstract

Many genes important in immunity are found as multigene families. The butyrophilin genes are members of the B7 family, playing diverse roles in co-regulation and perhaps in antigen presentation. In humans, a fixed number of butyrophilin genes are found in and around the major histocompatibility complex (MHC), and show striking association with particular autoimmune diseases. In chickens, BG genes encode homologues with somewhat different domain organisation. Only a few BG genes have been characterised, one involved in actin-myosin interaction in the intestinal brush border, and another implicated in resistance to viral diseases. We characterise all BG genes in B12 chickens, finding a multigene family organised as tandem repeats in the BG region outside the MHC, a single gene in the MHC (the BF-BL region), and another single gene on a different chromosome. There is a precise cell and tissue expression for each gene, but overall there are two kinds, those expressed by haemopoietic cells and those expressed in tissues (presumably non-haemopoietic cells), correlating with two different kinds of promoters and 5′ untranslated regions (5′UTR). However, the multigene family in the BG region contains many hybrid genes, suggesting recombination and/or deletion as major evolutionary forces. We identify BG genes in the chicken whole genome shotgun sequence, as well as by comparison to other haplotypes by fibre fluorescence *in situ* hybridisation, confirming dynamic expansion and contraction within the BG region. Thus, the BG genes in chickens are undergoing much more rapid evolution compared to their homologues in mammals, for reasons yet to be understood.

## Author Summary

Many immune genes are multigene families, presumably in response to pathogen variation. Some multigene families undergo expansion and contraction, leading to copy number variation (CNV), presumably due to more intense selection. Recently, the butyrophilin family in humans and other mammals has come under scrutiny, due to genetic associations with autoimmune diseases as well as roles in immune co-regulation and antigen presentation. Butyrophilin genes exhibit allelic polymorphism, but gene number appears stable within a species. We found that the BG homologues in chickens are very different, with great changes between haplotypes. We characterised one haplotype in detail, showing that there are two single BG genes, one on chromosome 2 and the other in the major histocompatibility complex (BF-BL region) on chromosome 16, and a family of BG genes in a tandem array in the BG region nearby. These genes have specific expression in cells and tissues, but overall are expressed in either haemopoietic cells or tissues. The two singletons have relatively stable evolutionary histories, but the BG region undergoes dynamic expansion and contraction, with the production of hybrid genes. Thus, chicken BG genes appear to evolve much more quickly than their closest homologs in mammals, presumably due to increased pressure from pathogens.

## Introduction

Many of the genes involved in immunity are part of multigene families. In some families, each gene is conserved for a specific function dedicated to a particular outcome, in others allelic polymorphism and copy number variation allow rapid evolution in response to new challenges, and in still other families both kinds of genes are found. Some well-characterised examples for adaptive immunity include genes of the major histocompatibility complex (MHC). For example, both the MHC class I and class II genes of humans and other higher apes have been relatively stable over 10 million years (My), whereas these genes have undergone many changes including extreme copy number variation (CNV) in monkeys [1], [2]. Further examples out of many are the genes encoding natural killer (NK) receptors, which not only undergo enormous CNV, but even use different structural families to carry out similar functions [3], [4]. Understanding the forces involved in this complex interplay of genomic structure, biological function and evolution is one of the challenges of modern genetics, with intense theoretical and experimental interest over many decades [for example: 5–22].

The regions in and around the mammalian MHC also include genes involved in innate immunity, such as the family of butyrophilin (and butyrophilin-like) genes for which an important role in the immune response is emerging. These genes are members of the B7 gene superfamily, many members of which are involved in immune co-regulation [23]–[26]. Some butyrophilin molecules function as inhibitory co-regulators, some may be involved in recognition of stress responses by γδ T cells, while others seem to have more specialised functions (such as synthesis of milk fat globules) and the functions of still others are as yet unknown [23]–[34]. Most importantly, butyrophilin genes have strong genetic associations with a variety of diseases in humans [35]–[49]. These genes encode transmembrane glycoproteins with two extracellular immunoglobulin (Ig)-like domains (one or four for butyrophilin-like molecules), and a few cytoplasmic heptad repeats followed by a B30.2 (or PRY-SPRY) domain [23]–[25], [34], [50], [51]. In humans, one of these genes is located in the MHC and the others in the extended MHC region, while in mouse some of these genes have been translocated elsewhere [23]–[25], [51], [52]. However, within each species the number and kinds of butyrophilin (and butyrophilin-like) genes seem to be fixed.

The SKINT genes are another multigene family within the B7 superfamily for which important roles in immune responses are emerging [24], [25], [53]–[55]. These genes have an extracellular V-like region related to butyrophilins and other members of the B7 superfamily, but have at least three transmembrane regions followed by short cytoplasmic tail. The SKINT1 gene is responsible for selection of a population of γδ T cells which become located specifically in mouse skin. Around the SKINT1 gene (located on a non-MHC chromosome) are several other SKINT genes and pseudogenes, the exact number of which varies between mouse strains. The single member of this family in humans is a pseudogene. Thus the SKINT family provides an example of B7 superfamily genes which appear to be evolving more rapidly than the butyrophilins.

Instead of butyrophilin genes, a related family of BG genes is found in and near the chicken MHC on chromosome 16. Indeed the chicken MHC was discovered as a serological blood group (the “B locus”) determining the highly polymorphic BG antigen on erythrocytes [56]–[59]. It is now clear that there is a multigene family of BG genes, with one gene in the MHC (the BF-BL region of the B locus) and an unknown number of BG genes in the nearby BG region of the B locus. It is also clear that BG genes are expressed, not only on erythrocytes, but with a wide tissue distribution and a number of associated immunological phenomena [60]–[68]. BG genes encode disulfide-linked dimers, each chain having a single extracellular Ig-like region (part of the V domain family) and a long cytoplasmic tail composed of many heptad repeats which presumably form an alpha helical coiled-coil. However, there is one chicken butyrophilin-like gene, Tvc-1, in the chicken genome which was described as the receptor for avian leukosis virus subgroup C, located on chromosome 28 [69].

Thus, chicken BG genes might be derived from ancestral butyrophilin genes, and perhaps have similarly important functions. Despite much speculation concerning associated immunological functions (reviewed in [59]), there are only two clear indications of functions for BG genes. One fortuitous discovery was the “zipper protein”, originally described as a soluble cytoplasmic protein which turned out to be the tail of a BG protein, and which has a role in controlling actin-myosin interaction in intestinal epithelial cells [70]. The other important study re-examined chickens that had been used to show that the BF-BL region (and not the BG region) determined resistance to the tumours caused by Marek's disease virus (an oncogenic herpesvirus) and Rous sarcoma virus (an acutely transforming retrovirus). By single nucleotide polymorphism (SNP) analysis and resequencing of genomic DNA, the authors found that a retroviral insertion into the 3′UTR of the single BG gene of the BF-BL region, the 8.5 or BG1 gene, correlated with resistance to the tumours. Moreover, they presented evidence that an immuno-receptor tyrosine-based inhibitory motif (ITIM) present in the cytoplasmic tail might be important to BG1 function [71]. Thus, the cytoplasmic tail has been identified as important in the two best studied examples of a functional effect for any BG gene, opening the question of what role the high level of polymorphism in the extracellular region might play.

It has become clear that the BG multigene family is quite complex in comparison to butyrophilin genes, and that an understanding of the true functions of particular BG genes will only be possible once a detailed picture of genomics and expression is available. In this paper, we provide the genomic organisation of the BG genes in the B12 haplotype, determine cell and tissue expression for each gene of the B12 haplotype, compare the B12 haplotype in detail with a red junglefowl haplotype used for the whole genome shotgun (WGS) sequence and at less resolution with five other haplotypes, and then consider what the data may mean in terms of multigene family evolution. The results take us to a new level of understanding, from which more detailed analyses can be launched.

## Results

### There are 14 BG genes in the B12 haplotype of C line chickens: One on chromosome 2, another in the MHC on chromosome 16 along with a cluster of 12 in the BG region

A cosmid library constructed from the genomic DNA of a CB congenic chicken line (B12 haplotype on a CC inbred chicken line background) had previously been used to define contigs, one of which (cluster I) was the BF-BL region (the classical MHC of the chicken) and three others (clusters II–IV) were later recognised as the Rfp-Y region (a region of non-classical MHC genes) [72]–[74]. We screened this library with a BG cDNA probe and picked 50 colonies, which were grown up. Analysis by Southern blot allowed the cosmid clones to be grouped into several contigs, but already many of the cosmids had suffered deletion of the BG genes, and in retrospect others had lost portions of their sequences.

**Figure 1:**
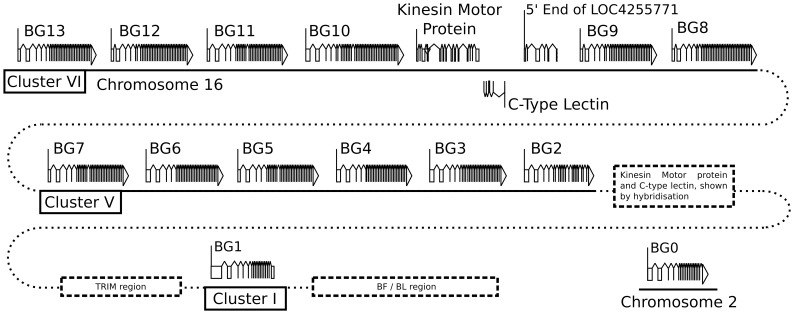
Fourteen BG genes of the B12 haplotype are present as two singletons (BG0 on chromosome 2, and BG1 in the BF-BL region or classical MHC on chromosome 16) and a cluster of twelve genes in the BG region on chromosome 16 (BG2-BG13, all in the same transcriptional orientation but separated into clusters V and VI by a region containing a kinesin motor protein gene, a C-type lectin gene, and an unassigned gene called LOC4255771). The genes are depicted with their introns, exons and intragenic regions to scale (except for regions with dotted lines) and in the orientation as typically shown for the chicken MHC and surrounding regions. The BG0 gene was discovered as a cDNA from a CB (B12) chicken caecal tonsil library, but the sequence of the gene is based on the whole genome shotgun sequence (release 2.1), located at positions 100590000–100600000 on chromosome 2.

Three authentic contigs were eventually defined by extensive restriction double digest mapping, subcloning, limited sequencing after PCR, and comparison to genomic DNA by Southern blot (Figure S1). One of these contigs corresponded to cluster I (the chicken MHC, or BF-BL region) which we had already shown contained a BG gene provisionally named the 8.5 gene (later renamed BG1). We fully sequenced the 8.5 gene (accession number KC963427, [59]), and later the whole of the cluster I contig (accession number AL023516, [73]). The other two contigs (named cluster V and VI) each contained six BG genes, which were given a variety of provisional names (now renamed BG2 through BG13). In addition, a related region without BG genes was found in each cluster. Three representative cosmids covering most of clusters V and VI were fully sequenced by standard shotgun techniques, confirming the presence of the six BG genes in each cluster along with a small region containing genes for a kinesin motor, a C-type lectin-like receptor and an unidentified protein (Figure 1, Figure S2, accession number KC955130).

We were concerned whether we had cloned all of the BG genes from the CB chicken. Screening revealed one additional B12 BG sequence (accession number KC955131) from one of our caecal tonsil cDNA libraries, which was called CTBG (and which we will now rename BG0). BLAST analysis of the chicken WGS sequence (www.ensembl.org, release 2.1) showed that this gene is present on chromosome 2 (positions 100590000–100600000), a different chromosome from the chicken MHC on chromosome 16 (Figure S3).

**Figure 2:**
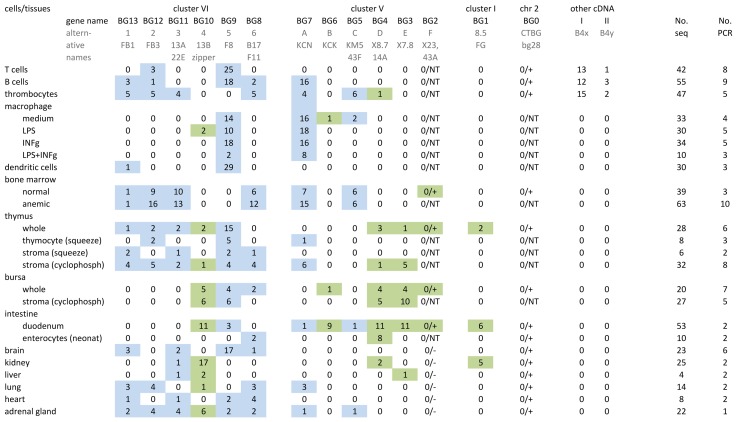
Individual BG genes of the B12 haplotype have striking expression patterns, as assessed by RT-PCR from cells and tissues using what were expected to be “universal primers” followed by cloning and sequencing. At the top, the heading of columns indicates the genes (with their present names along with alternative names previously used) in the same orientation as in Figure 1, and sequences labelled I and II apparently picked up from the B4 haplotype during derivation of CB congenic line chickens from the B12 haplotype of C line chickens. Also shown are the number of independent PCR reactions, and the number of total BG clones sequenced. On the left, the labels for rows describe the isolated cells and tissues from which the RNA was derived, along with separation techniques and treatments that were carried out (as described in Materials and Methods). Values in the table indicate the number of sequences found by RT-PCR, cloning and sequencing for each gene. After the work was well underway, it was realised that the primers were not “universal”, and therefore presence and absence of BG0 and BG2 were determined by specific primers (designated by a number for the sequences, followed by a plus or minus); NT indicates not tested. The coloured boxes indicate the results for presumed haemopoietic (blue) and tissue (green) genes. To be clear, complete separation of these expression patterns in tissues is not expected: all tissues contain blood vessels, some tissues contain tissue-resident macrophages and some tissues contain primary or secondary lymphoid tissue.

Using the partial sequences of all the genes identified at the time, we had designed potentially universal primers, and performed RT-PCR on a variety of cells and tissues (Figure 2, Figure S4). We found all of the genes from the cosmids expressed (except one, BG2, which we later realised had a single nucleotide change compared to the 3′ end of one of the primers). In addition, we found our supposedly universal primers did not amplify BG0, but specific primers showed that it has a wide if not ubiquitous tissue distribution.

In addition, we found several BG sequences from cDNA isolated from B12 haplotype chickens of the CB congenic line, but not B12 haplotype chickens from the parent C line. The congenic line CB (B12) was derived from the C line (which contains both B4 and B12 haplotypes) by backcrossing with the highly inbred CC (B4) line. The additional sequences (I, II, IIIa and IV) were eventually found to be BG genes from the B4 haplotype (Figure S4) that presumably were acquired during the backcrossing to produce the CB congenic chicken line.

**Figure 3:**
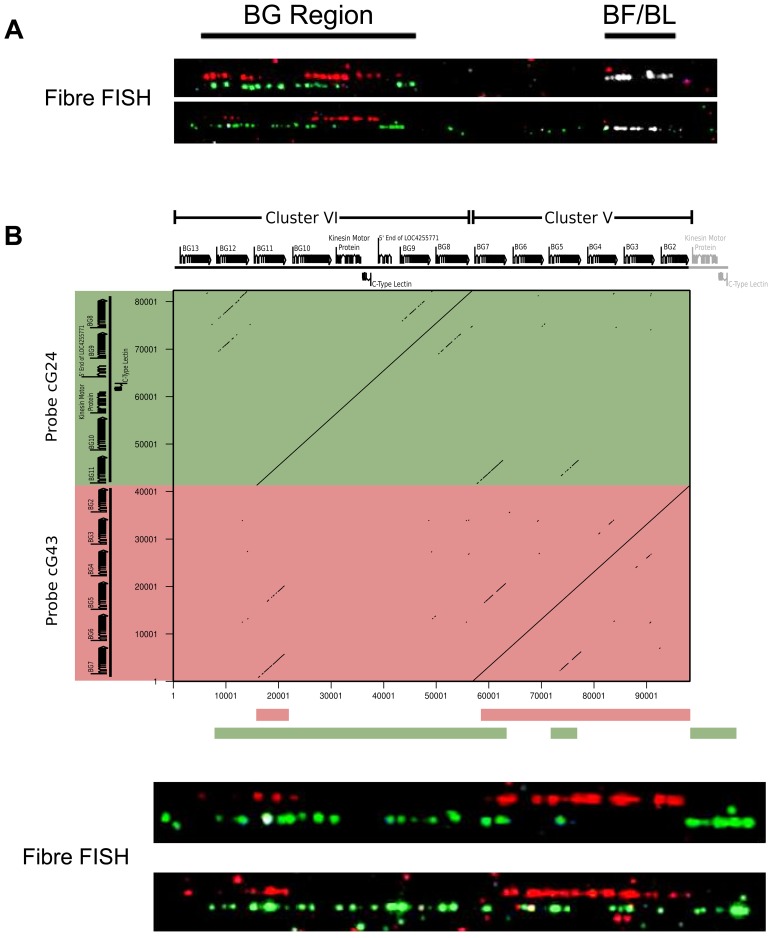
The two cosmid clusters are contiguous with the orientation cluster VI-cluster V, followed by the TRIM and BF-BL regions, as assessed by fibre-FISH and sequence comparison. A. Fibre-FISH of DNA from Con A-stimulated C-B12 spleen cells (B12 haplotype) with a BF-BL probe (cosmid c4.5 in white), a cluster V probe (cosmid cG43 in red) and a cluster VI probe (cosmid cG24 in green), with the image of red hydridisation shifted above for clarity. Note the single spot of hybridisation at the inner edge of the white hybridisation, which indicates the BG1 gene and correctly orients the BG region. B. Detailed comparison of two BG region probes indicates orientation of the two clusters. Upper panel, on top are the gene sequences for BG2-BG13 (as depicted in Figure 1), and to the left are the sequences for the two probes (cG43 for cluster V in red and cG24 for cluster VI in green), with a dot plot showing sequence identity (dottup program set to 150 nucleotide word size, as described in Materials and Methods). Lower panel, interpretation of hybridisation patterns expected based on the dot plot, compared to two representative examples of actual fibre-FISH, with cG43 in green and cG24 in red.

Finally, to ascertain the relative location of the clusters, we used some cosmid clones as probes in metaphase and fibre-fluorescence in situ hybridisation (fibre-FISH) of chromosomes from B12 splenocytes stimulated with the mitogen concanavalin A (Figure 3). We found a single large cluster of BG genes defined by hybridisation with cosmids from clusters V and VI, separated from the chicken MHC as detected by a cluster I cosmid. In some fibres, hybridisation corresponding to a BG gene was found at the end of the cluster I towards the BG cluster, which oriented the end of the MHC with BG1 toward the BG cluster. Comparison of the hybridisation pattern of the cluster V and VI probes with hybridisation expected by relative nucleotide sequence identity showed that cluster V and VI are contiguous, with cluster V closest to cluster I.

This organisation of the BG and MHC clusters was confirmed at the level of DNA sequence (Figure S5). Several BACs that span the BF-BL region through the TRIM region to some unidentified BG genes have been isolated from CB chickens [75]. Amplification from these BACs identified four BG genes located at one end of the cluster V contig, with the outermost being BG2, followed by BG3, BG4 and BG5. Similarly, amplification between BG8 of cluster VI and BG7 of cluster V physically linked these two clusters, with 1047 nucleotides of DNA between them.

Thus, we found 14 BG genes present in the B12 haplotype (as defined by the parent C line). There are two singleton genes, BG0 present on chromosome 2 (in the chicken WGS sequence) and BG1 found in the BF-BL region of the chicken MHC on chromosome 16. Upstream of the BG1 gene is a region containing TRIM genes among others, upstream of which is the BG region, the sequence of which is 99,274 nucleotides long. There are 12 BG genes located in this BG region, all in the same transcriptional orientation, and split into two clusters by the presence of kinesin, lectin and other genes.

### Each BG gene has very specific cell and tissue expression, with one group expressed in haemopoietic cells and another group expressed in tissues

As mentioned above, we performed reverse transcriptase-PCR (RT-PCR) on a variety of cells and tissues with what we thought at the time were universal primers (which however turn out not to amplify BG0 or BG2). For each cell and tissue, we cloned the PCR products and counted the number of clones from several independent amplifications, a method used successfully for assessing the relative expression of MHC genes [76], [77]. With this simple assay, we found truly striking patterns of expression for each of the analysed genes, with only a few genes expressed in each cell type and restricted patterns even for tissues (Figure 2). To provide additional support for this approach, we developed specific RT-qPCR assays for two haemopoietic and two tissue BG genes, and found that the results with spleen, bone marrow, liver and duodenum confirm our expectations based on the data from the approach of amplifying, cloning and sequencing (Figure S6).

In the blood cells of chickens with the B12 haplotype, T cells strongly express BG9 and express weakly BG12. B cells also express BG9 and BG12, but in addition strongly express BG7 and weakly express BG8 and BG13. In contrast, thrombocytes do not express BG9, but more evenly express BG5 and BG11 along with BG7, BG8 and BG13 (like B cells) and BG12 (like both B and T cells). Macrophages isolated from blood, whether or not treated with agents like lipopolysaccharide (LPS) and interferon-gamma (INFγ), strongly express BG7 (like B cells) and BG9 (like T and B cells). Dendritic cells developed in culture with IL4 and GM-CSF from precursors in the blood strongly express BG9 (like B cells, T cells and macrophages).

BG molecules were originally discovered as blood group antigens on erythrocytes, which do not contain appropriate messenger RNA. However, bone marrow from phenylhydrazine-treated chickens should be enriched compared to normal bone marrow in erythroid precursor cells which have RNA for the BG molecules found on erythrocytes. On this basis, erythrocyte BG molecules of the B12 haplotype may include BG7 (like B cells, thrombocytes and macrophages), BG8 (like B and thrombocytes), BG11 (like thrombocytes) and BG12 (like B cells, T cells and thrombocytes). Also found but not enriched were BG5 (like thrombocytes) and BG13 (like T and B cells).

Primary lymphoid organs such as thymus and bursa are the source of mature peripheral T and B cells, respectively. In these organs, precursor lymphoid cells undergo complex differentiation and selection events dependent on a variety of other cell types, including one or more types of so-called stromal cells. These stromal cells, at least in the thymus, are known to include both haemopoietic and non-haemopoietic cell types. As might be expected, the expression of BG genes in these organs is complex. Comparison of the expression of BG genes in thymus and bursa with the small lymphoid cells suggest that (at least some of) the precursor T cells (thymocytes) express BG9 and BG12 like T cells. At least some of the precursor B cells in the bursa may express BG9 like B cells, but there was no evidence of expression of BG7 (which was strongly expressed in B cells), BG12 or BG13 (more weakly expressed in B cells), so these latter three may be differentiation antigens in the B cell lineage. Comparison of stromal cell populations prepared by gradient centrifugation from precursor cells or by killing most of the rapidly dividing precursor cells with cyclophosphamide suggests that in the B12 haplotype the various stromal cells of thymus may express BG3, BG7, BG8, BG9, B12 and BG13, while the stromal cells of bursa may express BG3, BG4, BG9 and BG10.

In other tissues, the expression patterns were complex, which may be the result of a single cell type expressing several BG molecules, or may reflect the presence of multiple cell types each of which expresses certain BG molecules. Notably, we only detected BG1 expression in intestine (adult duodenum but not embryonic enterocytes), even though the original 8.5 genomic fragment was found to hybridise to RNA from chicken thymus, liver, a T cell line and a B cell line [72]. Also, we found intestinal expression of BG10, which has a nearly identical sequence of the cytoplasmic tail to the previously identified zipper protein (Figure S3), a protein described to regulate actin-myosin interaction in the intestinal epithelium [70]. Duodenum strongly expressed BG1, BG3, BG4, BG6 and BG10. By contrast, embryonic enterocytes expressed only BG4. Brain strongly expressed BG9 (if macrophage-like microglia are the source, then they differ from peripheral macrophages which strongly express both BG7 and BG9), while kidney strongly expressed BG10 (like stromal cells of thymus and bursa).

Overall, we were able to discern two types of expression, one primarily in haemopoietic cells and the other primarily in tissues (presumably from other cell types, at least some of which we expect to be epithelial/stromal cells). Haemopoietic BG genes include BG5, BG7, BG8, BG9, BG11, BG12 and BG13; while the tissue BG genes include BG1, BG3, BG4, BG6 and BG10. As described below, the assignments of haemopoietic- and tissue-type BG genes correlate perfectly with the relationship of the presumed promoter and 5′UTR of these genes.

### All the BG genes have similar structure, but there are many hybrid genes with the 5′ end determining cell and tissue expression

In order to better define these 14 BG genes, we compared them with published cDNA clones [38], [44], all of which were from other haplotypes, so we could not be sure whether we were comparing a cDNA with the appropriate gene. Using CLUSTLx, we found the same general organisation for every BG gene in the B12 haplotype (Figure 1): a first exon composed of roughly 200 nucleotide 5′UTR followed by a short signal sequence, a second exon encoding the immunoglobulin variable-like (Ig V-like) extracellular domain, a third exon encoding a connecting peptide and transmembrane region, a large number of small exons encoding 7 (or sometimes 8) amino acids which altogether would result in a cytoplasmic region with the potential to produce a coiled-coil, followed by an exon of the 3′UTR.

We then compared the predicted intron-exon structure with our authentic cDNAs of BG0 isolated from a B12 library or amplified from transfectants with BG1, BG10 and BG11 genes of the B12 haplotype (Figure S7). The exons encoding the V-like region, transmembrane region and the cytoplasmic domains were perfectly predicted, with the exceptions due to alternative splicing or read-through in the cytoplasmic exons (and one predicted cytoplasmic exon in BG10 that was not found). Similarly, the end of the first exon and the beginning of the last exon were perfectly predicted. For BG0, the WGS sequence showed two 3′ ends, but they turned out to be due to mis-assembly in the genome sequence (Figure S8). For BG1, BG10 and BG11, the location of the primers for amplification of the cDNA preclude assignment of the very 5′and 3′ ends of the genes. However, sequence comparison strongly suggests that 5′ of all the BG genes start at roughly the same place. Moreover, the 3′ ends seem clear from the conserved location of the single polyadenylation site in all genes.

**Figure 4:**
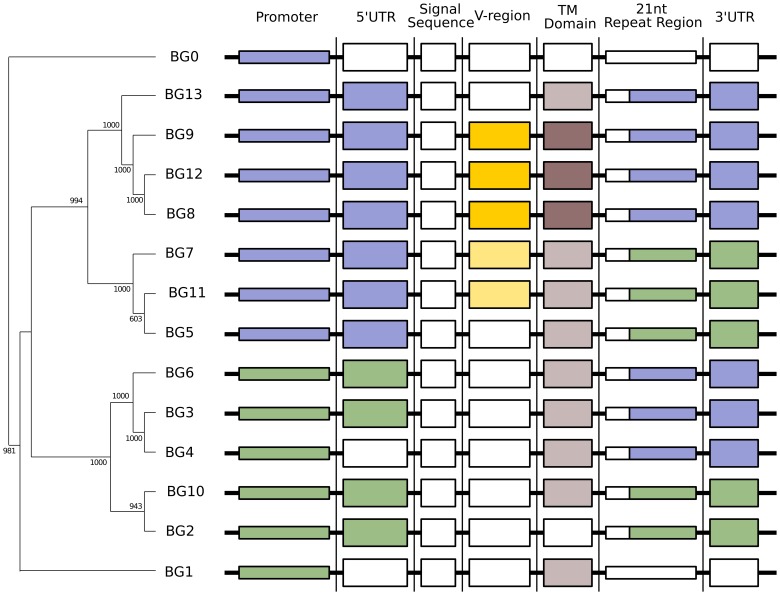
Phylogenetic analysis reveals six kinds of BG genes in the B12 haplotype: The two singletons each separately, and the twelve BG genes of the BG region in four groups indicating the presence of hybrid genes. Left, relationships of whole BG gene sequences (from 500 bp upstream to near the end of the 3′UTR as determined by the predicted polyadenylation site) as assessed by phylogenetic analysis (numbers at nodes indicate boot strap values determined from 1000 replicates). Right, relationships of different regions of BG genes indicated by colour, as determined by separate phylogenetic analyses in Figure 5.

In order to determine the relationship of the 14 BG genes, we performed phylogenetic analysis by neighbour joining (NJ) on the whole genes as well as portions of the genes. Dendrograms of the whole genes show two groups of paralogues, one for BG genes expressed in haemopoietic cells and the other for BG genes expressed in tissue BG genes in the BG region (Figure 4). As will become clear below, the topology of these trees depend on the relative amount of sequence from different parts of the gene.

**Figure 5:**
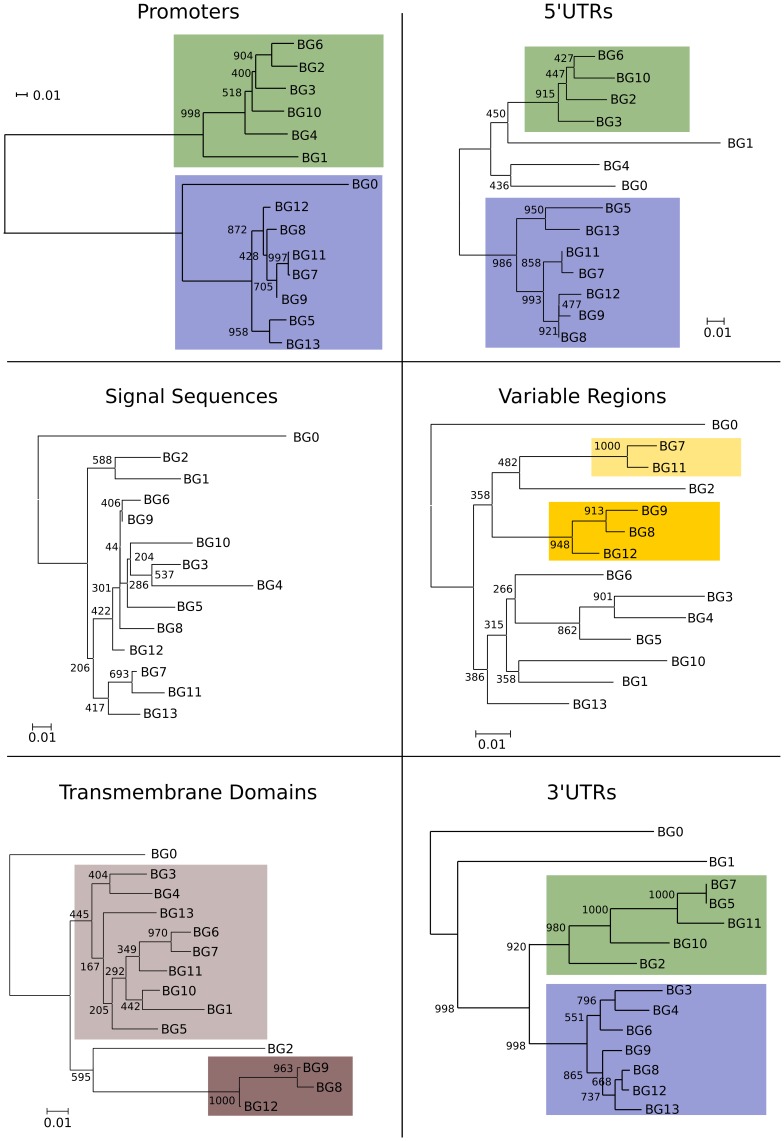
Phylogenetic analysis of nucleotide sequences for different regions of BG genes indicates separate evolutionary histories, consistent with recombination and/or deletion leading to hybrid genes in the BG region. The proximal promoters (500 bp upstream of the presumed transcriptional start site) and 5′UTRs fall into two well-supported groups that correlate with hemopoietic (blue) and tissue (green) expression as determined in Figure 2 (the separation of the BG0, BG1 and BG2 are due to short deletions in the 5′UTR, as seen by sequence alignment in Figure 6). In contrast, short branches with generally poor bootstrap support characterise the signal sequences and variable Ig-like regions. Transmembrane regions fall into two groups (as seen by sequence alignment in Figure 7), except for BG0. 3′UTRs fall into two well-supported groups, except for the two singletons, which include sequence of apparently distinct evolutionary origin at the very 3′ end.

The dendrograms of the presumed promoter (as defined by the sequence of the first 500 nucleotides upstream of the 5′UTR) and the 5′UTR (as defined by sequence similarity to published cDNA sequences) showed the same topology as the whole genes, a topology with long branches and strongly supported by the bootstrap values (Figure 5). Comparison of these trees to the expression patterns in Figure 2 indicates that the promoter region (and possibly the 5′UTR) of a BG gene is the primary determinant(s) of cell and tissue-specific expression.

There is almost no sequence identity between the two groups of promoters out to 1000 nucleotides before the 5′UTR. However, the analyses were carried out with 500 nucleotides corresponding to the proximal promoters, because the distal promoters of two BG genes contain sequence brought in from neighbouring genes (Figure S9). The distal promoter region of BG1 includes a duplication of a portion of the promoter region in between the neighbouring BNK and Blec genes, followed by a region of sequence of unknown evolutionary origin, and finally the proximal promoter region that is similar to other tissue BG genes. Similarly, the distal promoter of the BG9 gene in the B12 haplotype is unlike the consensus BG genes, and appears to have been derived from a hypothetical protein gene which is present in the red junglefowl haplotype but which has been deleted in the B12 haplotype (as shown below). This distal promoter sequence contains several brain-specific transcription factor binding sites (Figure S9), consonant with the expression in brain of BG9 in the B12 haplotype.

**Figure 6:**
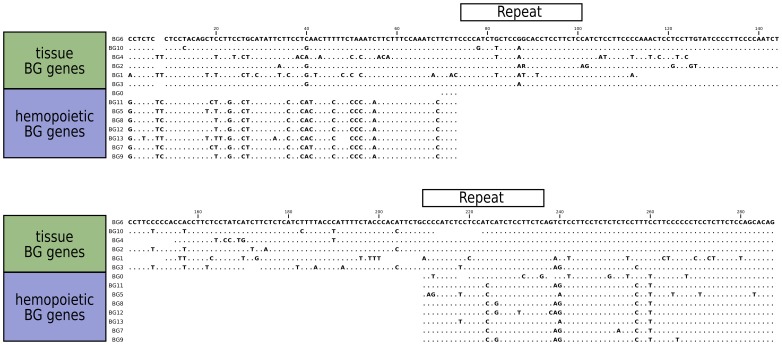
Sequence alignments for the 5'UTR of B12 BG genes, showing the separation into genes expressed in hemopoietic cells and in tissues. A large gap in genes expressed in hemopoietic cells was presumably created by deletion between two direct repeats indicated by boxes, and smaller gaps are found in the genes expressed in tissues.

The sequences of the 5′UTR of the BG genes also fall into two groups (Figure 6), with a large specific deletion in the haemopoietic BG genes compared to the tissue BG genes. It seems likely that the difference is a true deletion, since two 27 nucleotide direct repeats are found in the tissue 5′UTRs which upon recombination would yield the deletion found in haemopoietic 5′UTRs. The simplest interpretation is that all the haemopoietic BG genes in the B12 haplotype are descended from a single ancestor, but it is also possible that concerted evolution between BG genes could lead to the same result.

In contrast to the unambiguous dendrograms of the 5′ end, the region corresponding to the signal sequence and the extracellular V-like region formed dendrograms with completely different topologies, which had short branches and were generally poorly supported by the bootstrap values (Figure 5). However, there are three groups each consisting of very similar V sequences: BG3, BG4 and BG5; BG7 and BG11; and BG8, BG9 and BG12. The last group of genes also share a deletion in intron 1, resulting in an intron of 113–144 nucleotides for BG8, BG9 and BG12 compared to 352–354 nucleotides for all the other BG genes. All the signal sequences and V-like regions have the expected sequence features described for BG genes, including the lack of N-linked glycosylation sites in the extracellular domain. This means that, contrary to almost every other type I membrane protein, all BG molecules lack N-linked glycans (as previously shown for BG molecules from erythrocytes, [60]), a curious property that has not yet been explained.

**Figure 7:**
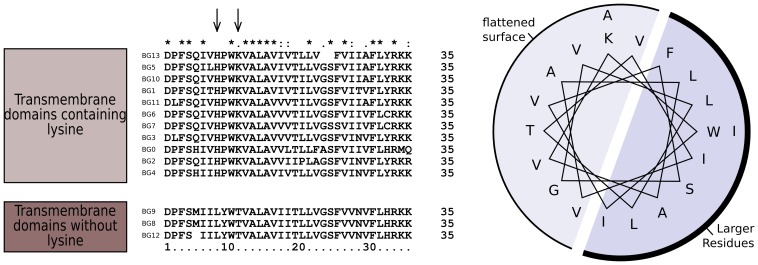
Sequence relationships for the connecting peptide to transmembrane region of B12 BG genes show two groups, those which have histidine and lysine near the N-terminus of the transmembrane region, and those with a leucine and threonine (arrows). A helical wheel shows that one side of an alpha helix through transmembrane region is primarily composed of larger residues (F, phenylalanine; I, isoleucine; L, leucine; W, tryptophan) along with a smaller residue (S, serine), while the other side is composed of smaller residues (A, alanine; G glycine; T, threonine; V, valine). This arrangement suggests that one side of the helix forms a flattened surface for interaction as a dimer, with the signature charged residue (K, lysine) near the edge of this interaction zone.

The dendrograms of the connecting peptide/transmembrane exon also yielded a tree (Figure 5) with short branches and low bootstrap values, but are separated into two broad groups. The sequences of these regions (Figure 7) are virtually identical among the BG genes, with a helical wheel depiction suggesting a flattened side for interaction between the two chains. In addition, some polar residues are found in most sequences, which in transmembrane regions can indicate specific interaction with polar residues of other chains. For all but three of these BG genes, the polar residues include two basic amino acids (histidine and lysine) near the start of the transmembrane region, but there is a well-supported group of three BG genes (BG8, BG9 and BG12) with hydrophobic leucine and polar threonine in those positions. All three are haemopoietic genes in which the V-like regions also form a group, perhaps indicating relatively recent duplication events. One gene (BG2) has a proline in the transmembrane region, which is most unusual. Finally, at the end of the transmembrane there is a tyrosine in all of the BG sequences except BG6 and BG7 which have a cysteine perhaps indicating a palmitylation site, and BG8, BG9 and BG12 which have a histidine.

The cytoplasmic tail is encoded by small exons, 21 (or sometimes 24) nucleotide exons long. The predicted number of such exons varies between BG genes in the B12 haplotype, ranging from 13 in the BG1 (8.5) gene to 36 in the BG10 (zipper protein-like) gene, with a mean number of 26 (Figure S2). Out of 358 total exons, we identified 57 different groups of nucleotide sequences (Figure S10). Removing exons present only once among in the 14 BG genes, we could discern clear patterns, particularly if the first roughly 20% of the exons were removed from analysis. The dendrogram (Figure 4, Figure S10) based on this last 80% (including exons present only once in the 14 BG genes) shows two groups separated by long branches and with strong bootstrap support, along with separate branches for BG0 and BG1. However, the dendrogram has a different topology than the whole gene, the promoter and the 5′UTR, and much like that for the 3′UTR (as shown below).

Some cDNAs show unspliced (or retained) introns for which the sequence remains in frame, and we suggested [78] that these extra stretches coding for protein have the potential for interesting functions. The original cDNAs for BG1 (8.5) have a long stretch of contiguous C-terminal sequence (accession numbers KC955132 to KC955136, [59]), but our analysis shows that this region is in fact due to what was originally an unspliced intron, because the flanking 21 nucleotide repeat exons can be identified which have apparently reasonable splice sites (Figure 2, Figure S11). Interestingly, this region has recently been identified as containing a functional immunoreceptor tyrosine-based inhibitory motif (ITIM) [71], fulfilling our original prediction. We examined all of the sequences for the possibility of unspliced introns with in-frame sequence, and found between one and five per gene (Figure S11). We found ITIMs in translated intron sequences of six other genes (BG3, BG6, BG8, BG9, BG12 and BG13), but translation of all of these introns gave stop codons almost immediately after the ITIM, which would lead to truncated cytoplasmic tails (Figure S11).

The 3′UTRs range from 465 to 481 nucleotides in length, encoded by BG13 and BG11 respectively. Dendrograms (Figure 5) show two groups with the same topology as the cytoplasmic exons, with long branches and good bootstrap support.

Overall, the 5′ end of the gene clearly defines two groups that reflect the tissue distribution, the 3′ end defines two different groups, and the region in between does not fall into simple groups. Phylogenetic trees constructed by Bayesian analysis and by Maximum Parsimony (MP) give comparable topologies as the NJ method (Figures S12 and S13), and AU and SH tests after MP analysis provide statistical support for the presence of the two groups at the 5′ end and the two groups at the 3′ end, but no clear groups in between (Figure S13). This result is most easily explained by the presence of hybrid genes (in the sense used in reference [20]) formed by recombination between the two ends, in which the middle of some (and maybe all) genes has been so randomised by recombination that no phylogenetic signal is left. Neighbour network analysis by SplitsTree, a Phi test and an automated partitioning algorithm all support a history of extensive recombination across the BG genes, with independent histories for the 5′UTR, the V-like region and the 3′UTR (Figures S14 and S15). Recombination is certainly a plausible explanation for the sequence relationships found, since the 12 BG genes in the BG region are all close together in the same transcriptional orientation, so hybrid genes could be produced either by unequal crossing-over (through interchromosomal recombination, also known as non-allelic homologous recombination or NAHR) or by deletion (through intrachromosomal recombination) during meiosis. One of the consequences of such unequal crossing-over or deletion is expansion and contraction of this part of the multigene family, leading to copy number variation (CNV) in the BG region.

**Figure 8:**
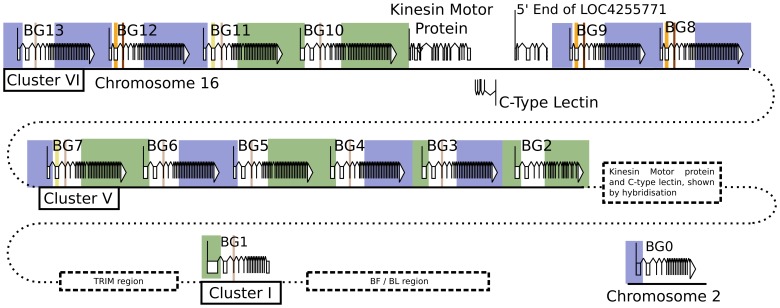
The presence of hybrid BG genes in the B12 haplotype shows no obvious pattern, consistent with a random process of recombination in the centre of the genes. The 14 BG genes of the B12 haplotype (as in Figure 1) are depicted with coloured boxes illustrating presumed origin (as in Figure 5). See Figure S11 for an alternative view.

In this view, haemopoietic genes have either their original haemopoietic 3′ end or a tissue 3′ end, and tissue genes have either their original tissue 3′ end or a haemopoietic 3′ end. Unfortunately, with the data at our disposal, we cannot be absolutely sure which is which, so for the time being we will refer to the 3′ ends as type 1 and 2. Thus, BG8, BG9, BG12 and BG13 might be pure haemopoietic genes, BG5, BG7 and BG11 might be haemopoietic genes with a tissue 3′ end, BG2 and BG10 might be pure tissue genes, and BG3, BG4 and BG6 might be tissue genes with a haemopoietic 3′ end (Figure 8). Alternatively, BG7 and BG11 might be pure haemopoietic genes, BG5, BG8, BG9, BG12 and BG13 might be haemopoietic genes with a tissue 3′ end, BG3, BG4 and BG6 might be pure tissue genes, and BG2 and BG10 might be tissue genes with a haemopoietic 3′ end (Figure S16).

### Definition of BG genes in a red junglefowl haplotype and comparison with the B12 and other haplotypes shows evidence of expansion and contraction of the multigene family through deletion of genes and swapping of whole BG clusters

The WGS sequence was created from a chicken of the UCD001 line, an inbred red junglefowl line with the BQ haplotype, closely related to the standard B21 haplotype in experimental lines of chickens derived from egg layers [79]. Other than BG0, BG1, BG2 and BG10 (with BG10 being zipper protein-like), no BG genes were correctly identified by ENSEMBL in this genome sequence.

**Figure 9:**
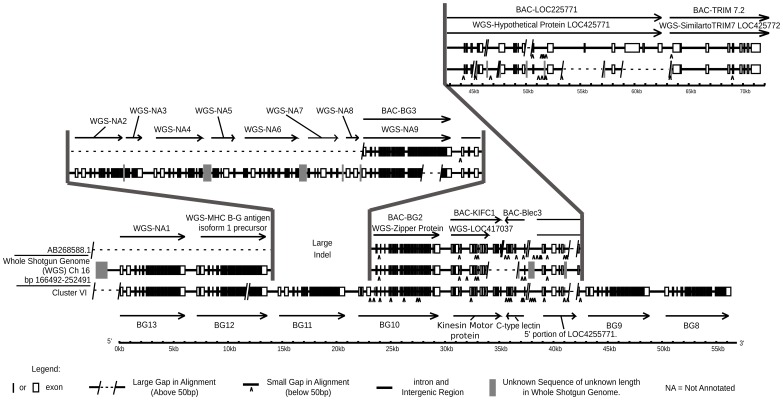
Comparison of cosmid cluster VI from the B12 haplotype with the BQ haplotype from a red junglefowl, showing regions of virtual identity separated by two large indels, one in the middle of the sequences and the other where the red junglefowl haplotype (but not the B12 haplotype) continues into the TRIM region. Genomic organisation on bottom line is from cluster VI of this paper (accession number KC955130) compared to two sequences from the BQ haplotype, middle line from the WGS sequence assembly (nucleotides 166492–252491 on chromosome 16) and top line from the sequence of a BAC from the same individual chicken (accession number AB268588.1). Note that there exist differences between the WGS and BAC sequences, and further that the WGS assembly has regions of unknown sequence with only approximate length. WGS-NA indicates genes not annotated by ENSEMBL at the time of this analysis.

By using BLAST to probe with a 3′UTR sequence, seven BG genes arranged in tandem and in the same transcriptional orientation were identified on a supercontig (covering contigs 318.1 to 318.6) in the contiguous sequence for chromosome 16 (Figure S17). The automatic annotation programme GENSCAN utilised by ENSEMBL apparently did not recognise the 5′ ends of these BG genes, and therefore they were only predicted as producing a single long transcript. The position and orientation of this cluster was verified by comparison to a BAC contig from the same chicken [80], from which the first two BG genes as well as a lectin-like gene, a kinesin gene and the intervening downstream TRIM region had been sequenced (Figure 9). However, in the portion for which there is only the WGS sequence, there are gaps in assembly that raise the possibility of an additional two BG genes.

Direct sequence comparison of the red junglefowl sequence from the BACs, the red junglefowl sequence from the WSG sequence and the B12 sequence from the cosmids (Figure 9) shows that there has been a precise replacement of the BG11 gene in the B12 haplotype with at least four genes in the red junglefowl haplotype, with 99.98 and 98.90% sequence identity between the two haplotypes on the left side and the right side, respectively, of the breakpoints. Moreover, the red junglefowl sequence goes directly into the TRIM region after the lectin-kinesin gene pair, whereas the B12 sequence has two additional BG genes after the lectin-kinesin gene pair and no indication of the TRIM region. There are also some deletions in the WGS sequence compared to the BAC sequence, which may reflect differences in the exact haplotypes or in sequence assembly. However, this comparison strongly supports the notion that recombination leads to strong differences between BG haplotypes.

**Figure 10:**
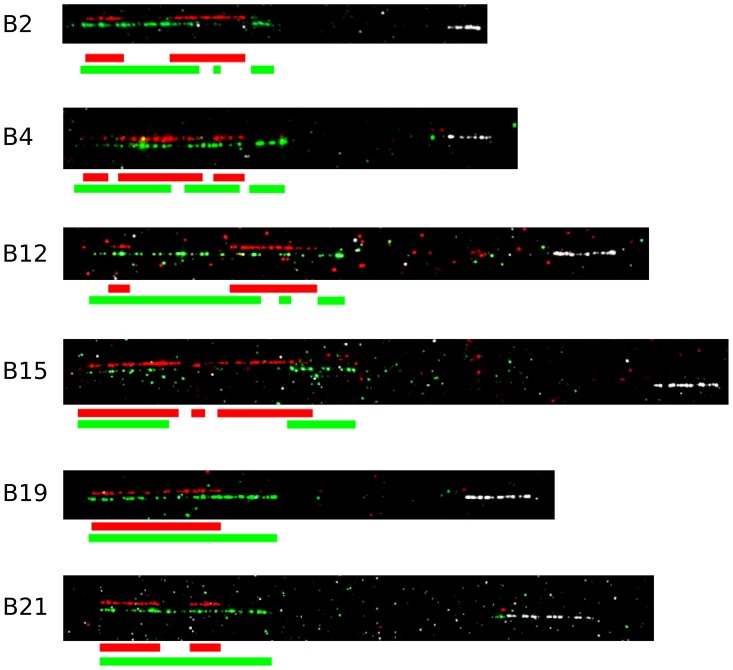
The BG regions of six haplotypes are located in the same orientation from the BF-BL region, but vary in size and composition, as assessed by fibre-FISH using probes corresponding to the cosmids cG43 from BG cluster V (red), cG24 from BG cluster VI (green) and c4.5 from BF-BL cluster I (white). Each panel is representative of several fibre-FISH experiments with genomic DNA from B2 (IS2 cell line), B4 (identical in BF-BL region with B13, UG5 cell line), B12 (Con A-stimulated spleen cells), B15 (TG15 cell line), B19 (IS19 cell line) and B21 (TG21 cell line).

In addition, at least nine red junglefowl BG genes arranged in tandem were identified in the bin “chromosome 16 random”, which consists of contigs predicted to be on chromosome 16 but not assembled with the contiguous portions of the WSG sequence (Figure S17). The order of these genes is not known, but on the basis of fibre-FISH they form another cluster, located next to the first red junglefowl cluster (Figure 10). Thus, there appears to be in the neighbourhood of 18 BG genes in the BG region of the red junglefowl haplotype compared to 12 BG genes in the B12 haplotype, demonstrating CNV for the BG region.

Phylogenetic comparison of these two BG clusters from the red junglefowl haplotype with the B12 haplotype showed that the first red junglefowl cluster is highly related to cluster VI from the B12 haplotype, but the second red junglefowl cluster is not closely related to any of the other clusters (Figure S18). Fibre-FISH shows that the two red junglefowl clusters are contiguous, based on their length and hybridisation to the B12 clusters (Figure 10), and the evidence from comparison to the reported BAC sequence locates and orientates the first cluster next to the TRIM region. Thus, the order of the clusters in B12 is BG cluster VI-BG cluster V-TRIM region-BF/BL region whereas the order of the clusters in red junglefowl is Second BG cluster-First BG cluster (related to cluster VI)-TRIM region-BF/BL region. This remarkable result is most easily explained by large-scale expansion and contraction events in the BG region, with whole clusters swapping in and out.

To test whether the differences between the B12 and red junglefowl sequences were due to one of them being an outlying variant compared to most MHC haplotypes, we performed fibre-FISH on an additional five haplotypes (B2, B4, B15, B19 and the true B21 haplotype). It is apparent that the order of the BG, TRIM and BF-BL regions is stable, but that the BG regions vary in size and order of BG genes (Figure 10). Thus it would appear that the expansion and contraction of the BG genes in the BG region is a general phenomenon.

## Discussion

For the first time in the study of BG genes, we have an understanding of the genomic organisation of a complete BG haplotype, coupled with a comparison to other BG haplotypes and a determination of cell and tissue expression. Two overarching points emerge among the many new findings, which together portray BG genes as a much more dynamic and complex genetic system than their closest mammalian homologues, the butyrophilin genes.

The first major point that we establish in this paper is the very specific cell and tissue expression for each of the BG genes, which overall form two groups (along with one gene that may have a more ubiquitous tissue distribution), strongly supported by the phylogenetic analysis of the presumed promoter regions. Although BG molecules were first discovered as a polymorphic antigen on erythrocytes, it has been clear for some years that there is a multigene family of BG genes, at least some of which were expressed in other cell types, including thrombocytes, B and T cells, bursal and thymic stromal cells, and intestinal cells [63], [65], [78], [80]–[82]. However, there has never been a complete list of BG genes for a haplotype, nor a comprehensive analysis of which genes are expressed in which cells and tissues.

In this paper, we examine all the BG genes of the B12 haplotype both by sequence and expression analyses and find that some BG genes are expressed in one or another cell of the haemopoietic lineage while other BG genes are expressed in tissues, likely from non-haemopoietic lineages. These assignments are strengthened by the fact that the 5′ ends (putative promoter and 5′UTR) of the genes from the BG region also fall into two groups which fit exactly with the presumed cell and tissue distributions (with the exception of the singleton BG genes, discussed below). Interestingly, the haemopoietic genes of the B12 haplotype all have a deletion within the 5′UTR, which almost certainly arose by recombination between two 27 nucleotide direct repeats found in all tissue BG genes. These data might be interpreted to suggest that all haemopoietic BG genes descended from a single BG gene, with the tissue BG genes being ancestral.

Within these broad categories of haemopoietic and tissue BG genes, the specificity of expression of particular BG genes in a single cell type is remarkable, with some genes changing expression during differentiation. For instance, only one BG gene in the B12 haplotype is strongly expressed in T cells sorted from peripheral blood. In contrast, two BG genes are strongly expressed in B cells sorted from peripheral blood, but one of these was not found in bursa, the primary lymphoid organ for the production of B cells. Changes in expression during differentiation are also suggested for the BG3 gene, which is strongly expressed in T and B cells, thymus and bursa, macrophages and dendritic cells, but not thrombocytes nor bone marrow from which all haemopoietic lineages are thought to originate. Interestingly, the BG3 gene is also strongly expressed in brain, and at least one transcription factor binding site specific for neurones is found in the putative promoter of BG3. Expression of particular genes may also change during activation of a cell type, but for macrophages a number of strong stimuli failed to affect the two strongly-expressed BG genes, BG3 and BG13. Overall, much more work needs to be done to explore the complex expression patterns of genes from the BG region.

Of the two genes located outside of the BG region, BG0 has an apparently ubiquitous tissue distribution while BG1 is expressed in intestine and kidney. The 5′ regions of these two genes are different from the other BG genes; the BG1 promoter is in fact partly composed of inverted pieces of the promoters of neighbouring genes.

The second major point that we establish in this paper is the presence of BG genes with different evolutionary histories, some relatively stable and others changing rapidly. As mentioned above, there has long been evidence for multiple BG genes, many of which were located to the BG region and one located to the BF-BL region [62]–[64], [67], [78], [80]. In this paper, we report two single BG genes with relatively stable evolutionary histories compared to the many BG genes located in a cluster of clusters, for which there is dynamic expansion and contraction and thus a much more complex evolutionary history.

The singletons located outside of the BG region are the newly described BG0 gene on chromosome 2, and the long-studied BG1 gene located in the BF-BL region. Although they have similar intron- exon structures and both have promoters different from other BG genes, many other features differ between these genes. We found BG0 as a cDNA from B12 caecal tonsil (a gut-associated lymphoid tissue), and comparison with available sequences showed that it was nearly identical to a fragmentary cDNA clone reported as isolated from B21 embryonic erythrocytes [67]. Specific PCR amplification shows that it has a wide, perhaps even ubiquitous, tissue distribution, and is present in every haplotype examined as a nearly monomorphic transcript (Chattaway, Salomonsen and Kaufman, unpublished). These properties suggest that BG0 has a biological function that is stable and well-conserved, perhaps homeostatic. In contrast, BG1 was originally described as expressed in liver, thymus, a T cell line and a B cell line [72], but our tissue distribution shows that it is well-expressed in thymus, intestine and kidney. Orthologous sequences are present in every haplotype examined, but there is allelic sequence variation throughout the gene, including expansion and contraction of the cytoplasmic exons [71], [83](Chattaway, Salomonsen and Kaufman, unpublished). The properties of BG1 suggest a function that is under some selection to change, perhaps in response to changes in pathogens (as has been suggested by genetic evidence, [71]). However, another possibility that has not been ruled out is that the genetic variation is due to hitch-hiking on nearby MHC genes which are under strong selection for variation [84], [85].

At a descriptive theoretical level, the presence of BG genes as singletons outside the BG region is best understood as a consequence of the “birth and death model” of multigene family evolution [17], [21], for which it has been noted that single gene duplicates can arise by replicative translocation [15]. Moreover, such theoretical considerations suggest that evolution of new functions is likelier in such singletons compared to a tightly-linked multigene family [15], [19]. More recent evolutionary dynamics of these BG singletons is more likely to be governed by a model of divergent evolution for alleles [17], [21].

In contrast, the multigene family of BG genes present in the BG region is organised as a cluster of clusters, and is undergoing significant expansion and contraction. The identification of clusters is based on the presence of non-BG genes at or near the end of each proposed cluster, a kinesin motor gene, a C-type lectin-like gene and an unclassified open reading frame. These genes in a characteristic order are found near the end of each of the B12 cosmid clusters V and VI, and were also identified at the junction of the BG region with the TRIM region in the red junglefowl haplotype used for the WGS sequence [36], [54]. These genes may be considered as “framework genes” in the sense originally proposed by Amadou [86], in which nearly single-copy genes with stable functions flank regions of expanding and contracting multigene families of genes with various functions.

In this paper, we present five pieces of evidence consistent with significant expansion and contraction leading to CNV of the BG region by recombination and deletion: presence of cDNA sequences derived from the B4 haplotype in congenic B12 chickens, presence of apparent hybrid genes in the B12 haplotype by phylogenetic analysis of sequence, deletion of genes evident from sequence comparisons of the proximal cluster of BG genes in the B12 and red junglefowl haplotype, apparent swapping of clusters by comparison of B12 and red junglefowl haplotypes by sequence and fibre-FISH, and differences in length and specific hybridisation patterns by fibre-FISH between six BG region haplotypes. Such change within the BG region is consistent with early biochemical evidence of recombination based on analysis by two-dimensional gel electrophoresis [80], [87].

At a descriptive theoretical level, the appearance of the BG multigene family might be best explained by the “birth and death” model [17], [21], in which duplication leads to multiple gene copies out of which some genes may be retained, while others become nonfunctional. At the moment, there is no obvious evidence for homogenisation of BG genes, so the question does not arise whether there is birth and death followed by purifying selection or concerted evolution by ongoing sequence exchange resulting in homogenisation [14], [15], [17]. However, one of the hallmarks of the “birth and death” model is considered to be the presence of pseudogenes [17], [21], and there is no evidence that any of the BG genes examined are non-functional, given that they all have long open reading frames and they are all expressed at the RNA level. One possibility is that in other BG haplotypes there are pseudogenes that have yet to be described. Another possibility is that the maintenance of the BG multigene family in the BG region is based on a so-called “mixed model” [17], [21], perhaps with sequence exchange repairing any pseudogenes. A third possibility is that a new theoretical model should be considered. In any case, the current evidence suggests that changes in BG genes within the BG region occur by unequal crossing-over and deletion without the obvious appearance of pseudogenes. The importance of recombination in the evolution of these BG genes seems clear, but at the moment there is no evidence to suggest how fast it might be in comparison with other multigene families [22], although the appearance of B4 BG genes in the congenic CB chicken line during back-crossing suggests that it could occur over a few generations. Whatever the speed of such recombination, without selection the number of BG genes would likely gradually reduce to one [9], so this suggests that selection for function is also an important part of BG evolution.

Such rapid evolution based on various outcomes of recombination is not easily reconciled with models for simple specified functions of all the BG molecules encoded by the BG region. We have found that the 5′ and 3′ ends of BG genes can often be switched to make hybrid genes, with the phylogenetic signal of the middle portion of the gene apparently scrambled. However, we also found that the 5′ end of the genes determine cell and tissue expression. It has long been postulated that the extracellular V-like region in the middle of the gene is involved in some immunological or cell-cell interaction function, and the cytoplasmic tail at the 3′ end in interactions with the cytoskeleton [59]. Indeed, there is direct biochemical evidence that the cytoplasmic tail of zipper protein (similar to the BG10 gene of the B12 haplotype) can regulate actin-myosin interaction in the intestinal brush border, and an implication that variation in the number of cytoplasmic exons of BG1 is involved in resistance to certain viruses [70], [71]. The conundrum is how there can be a stable function based on the middle or 3′ end of a gene, when expression of this gene can suddenly be switched by recombination bringing in a new 5′ end to another cell or tissue, presumably a random genetic event. The resolution of this apparent conundrum is one of the next important tasks.

One possibility is that there are BG genes within the BG region that are relatively stable with important and specific functions, and that between them there are expansions and contractions of genes whose expression and function can change rapidly. The existence of such stable “framework genes” flanking regions of genomic change has been particularly well-characterised for the killer inhibitory receptor (KIR) gene cluster which encodes receptors on natural killer cells [3], [20], [88], [89]. Indeed, the KIR genes show other similarities to the properties of the BG genes, including “tail-swapping” in which the inhibitory and activating 3′ ends of genes with different 5′ extracellular regions are switched [90]. If this model of genomic evolution of the BG region is correct, we expect to find some orthologous genes in every BG haplotype, whose alleles will carry out similar functions and be expressed in similar cells and tissues.

Identifying and characterising such framework genes in the BG region is another of the next important tasks. As an example, the sequence of the zipper protein characterised in an unknown chicken line is nearly identical with BG10 of the B12 haplotype, suggesting that this may be a framework gene with a highly conserved function and cell expression. In fact, the zipper protein was discovered as a protein which can control actin-myosin interactions in intestinal brush borders [70], likely to be a homeostatic control for which rapid evolution would not be advantageous.

Another possibility is that there is sufficient functional redundancy between different BG proteins to relieve the selective pressure to maintain the expression of individual genes. If this was the case, perhaps the same function could be achieved in different haplotypes by structurally and functionally similar but not necessarily orthologous genes. At a descriptive level, this kind of evolution might be considered “subfunctionalisation”, in which newly duplicated genes share and then partition the functions of the original gene [14]. It has been noted on theoretical grounds that tight linkage increases the probability of subfunctionalisation at the expense of neofunctionalisation [15]. Unravelling which genes in different haplotypes may serve the same and different functions is a third important task.

At least some of the BG genes that undergo rapid evolutionary change will almost certainly have important functions as well, characterised by the need for diversity and rapid response to changing environmental conditions. The likeliest scenario is a molecular arms race with pathogens, in which the diversity of such BG genes is driven by the ever-changing variation in (certain) pathogens. As mentioned above, resistance and susceptibility to two oncogenic viruses have recently been ascribed to a retroviral insertion in the 3′UTR of the BG1 gene [71]. A fourth important task is thus to understand the mechanisms of how such BG genes enable an effective response to a pathogen.

## Materials and Methods

### Animals

Samples were taken from experimental chicken lines kept at the Basel Institute for Immunology in Switzerland, the Institute for Animal Health in the UK, the Ludwig Maximilians University in Germany, and the University of Cambridge in the UK. The origins and derivations of the chicken lines used in this work are described in some detail [77 and references cited therein]. All animal work was conducted according to the relevant national guidelines in place at the time of the research. Most recently, approval for animal research was through UK Home Office Licenses and local Ethics Committees at the Institute for Animal Health at Compton, and the University of Cambridge.

### Isolation and sequencing of BG genes (Figures 1, S1 and S2)

Standard molecular biology techniques used in this paper are described in detail [91], [92], which typically are referenced along with modifications in the accompanying citations given below. Southern blotting using BG cDNA probes [78] identified the transcribed fragment 8.5 from various cosmid clones of the line CB (B12) chicken [72] as a BG gene (subsequently named BG1). A 6.5 kB fragment from cosmid cβ12 [72] cut with Nru I and Hind III was isolated and cloned into Bluescript (cβ12NK6.5BS2a clone), subclones were sequenced as described [93] by dideoxynucleotide technology using ^32^P-labelled ATP, some portions after isolation of single strands, and the sequence was deposited in Genbank (accession number KC963427).

A cosmid library from a line CB (B12) chicken [72] was screened with cDNA clones G1, G5, G7 and G8 [53] labelled with ^32^P by nick-translation. The 50 positive plaques were picked and grown up for isolation of DNA, which was analysed by Southern blot using various BG cDNA probes isolated from a H.B19 (B19) chicken spleen library [78]. Double-digestion restriction maps and limited sequencing were used to group the cosmids into clusters [59], with extraneous sequence of chimeric inserts determined by genomic Southern blots. DNA from cosmids cG43, cG3 and cG24 was sheared, cloned and sequenced using commercial fluorescent dye reaction kits followed by capillary electrophoresis at the Sanger Institute. The clusters were linked using the data from Figure S5C in Cambridge, and the whole sequence deposited in Genbank (accession number KC955130).

### Polymerase chain reaction

Amplification from DNA and cDNA was carried out using different commercial kits at different times during the work described in this paper. Typically, the amplifications were carried out with commercial kits using manufacturer's instructions (sometimes optimised for Mg, dimethylsulfoxide or polyethyleneglycol concentration), with 2–5 min at 94–96°C for initial denaturation, followed by 30 cycles of 0.6–1 min at 94–96°C for denaturation, 1–2 min at a lower temperature (depending on the primers) for annealing and 1–5 min (depending on amplicon length) at 72°C for extension, and ending with 10 min at 72°C for final extension followed by storage at 4°C.

### Isolation of BG cDNA for comparison to genomic sequences (Figures S3 and S7)

For BG0, clones were isolated from a cDNA library in Basel. Briefly, RNA was isolated from caecal tonsil of a CB chicken and cloned into λZAP vector to make the CT-2 library (much as described in [94]), which was screened with BG cDNA probes as above. Phage clones were picked and converted by plasmid clones by in vivo excision with a helper phage VCSMI3 in BB4 cells. DNA was prepared by the CTAB miniprep method, and the length of insert was determined by digestion and Southern blotting using BG cDNA [78] and genomic clones from the BG1 gene as probes, with clones 45A, 47B and 47C eventually picked for full sequencing using a T3 thermocycler (Biometra), a fluorescent dye terminator kit and a 373A DNA sequencer (both Applied Biosystems).

For BG1, BG10 and BG11, cDNA clones were isolated from transfectants in Basel. Briefly, mouse L cells with inactivated thymidine kinase gene (Ltk^-^ cells) were transfected by standard calcium phosphate procedures [91], [92] with clones cβ12NK6.5BS2a, cG13 and cG22_2_. Cells expressing BG genes were selected by neomycin, then enriched by fluorescence-activated cell sorting and cloned by limiting dilution followed by screening using flow cytometry, in both cases using a pool of mAb to BG including BG2, 3, 4, 5, 6 and 9 [65]. Total RNA was prepared from clones 1.4 and 1.7 (cβ12NK6.5BS2a containing BG1 gene), 6N.39 (cG13 containing BG10 and BG11) and 8N.37 (cG22_2_ containing BG10 and BG11) using the FastTrack kit (Life Technologies) and cDNA was prepared using Superscript reverse transcriptase (Stratagene). BG cDNA was amplified using a T3 thermocycler, a commercial Taq polymerase kit (Roche), with an annealing temperature of 45°C. BG1 cDNAs were amplified using reverse primer 2773 containing a Not I site (ATATATgcggccgcCTYTGGTTTCTTTCTTCCAATTGG) based on the cDNA clones G3 and G8, and a series of forward primers each containing a Sal I site, 2960 (TATATgtcgacTGGCAGAATTACTGGTGCC), 2961 (TATATgtcgacCTGGTGATAGCAGAGACCC) and 2962 (TATATgtcgacGGTAGAAGCTGGGC) designed based on the genomic sequence (accession number KC963427) to establish the 5′ end of the BG1 transcript. BG10 and BG11 cDNAs were amplified with forward primer 8081 containing an Nru I site (CACGTtcgcgaCCATGSNSTTYNYATYRRGMTGC) and reverse primer 2774 (sequence above). Amplified fragments were cut with appropriate restriction enzymes, gel-purified and cloned into Bluescript plasmid, with BG1 clones PCRX1, 3 and 5 from transfectant 1.7 and PCRX11, 29, 31 and 35 from transfectant 1.4, BG10 clones 34, 42, 47 and 50, and BG11 clones JK29, 32, 37, 45 and 49 eventually chosen for full sequencing using a T3 thermocycler (Biometra), fluorescent dye terminators kit and a 373A DNA sequencer (both Applied Biosystems).

### Preparation, amplification and sequencing of cDNA from cells and tissues (Figures 2 and S4)

The cDNA preparations from T cell, B cell and thrombocyte populations purified by flow cytometry from blood of CB chickens in Basel were described in previous publications [76], [77].

The cDNA preparations from macrophages were prepared from blood monocytes isolated from CB chickens in Munich, essentially as described [95]. Briefly, leucocytes from heparinised blood were separated by centrifugation through Ficoll-Paque. Cells at the interface were washed twice in PBS, adjusted to 2×10^7^ cells/ml in RPMI 1640 supplemented with 10% FBS, 100 U/ml penicillin and 100 µg/ml streptomycin, and then incubated on 90-mm cell culture Petri dishes at 5% CO_2_ and 40°C. After 48 hours non-adherent cells were removed by vigorous washing. The remaining cells were over 98% positive for KUL01, a macrophage-specific monoclonal antibody [96], and were further incubated in the same medium under identical conditions with or without activation. Cells were either stimulated with LPS from *E. coli* (O127,B8; Sigma) at a final concentration of 10 µg/ml or with cell culture supernatant of chicken INFγ expressing COS cells [97] at a final dilution of 1∶500 or with a combination of both. After 24 hours cells were washed with PBS and harvested into peqGOLD TriFast (Peqlab, Erlangen, Germany) and RNA extracted according to the manufacturer's protocol. In order to confirm macrophage activation cell nitric oxide release into cell culture supernatant was quantified by Griess reaction [98].

All other cells and tissues were from C-B12 chickens in Compton, with total RNA isolated in TRIzol and cDNA made with Superscript III (Invitrogen). Bone marrow from untreated and chickens rendered anaemic with phenylhydrazine was isolated as described [78]. Dendritic cells were derived from bone marrow and grown in chicken IL4 and GM-CSF for 7 days as described [99]. Intestinal enterocytes from embryonic day 14–15 embryos were isolated from the PBS-45% Percoll interface as described [100], [101]. Lymphocyte depletion was achieved by daily intramuscular injection of 3 mg cyclophosphamide each of the first 4 days after hatching [102]. Thymus and bursa tissues were disrupted by passing through a nylon sieve, and lymphocytes separated from stromal cells by 70%–45% discontinuous Percoll gradient with a PBS overlay [97].

Amplifications in Copenhagen were performed using a GeneAmp PCR System 2700 thermal cycler (Applied Biosystems) using either the High Fidelity PCR Enzyme kit (Fermentas #K0192) or Long PCR Enzyme kit (Fermentas #K0182). Samples of cDNA (usually 1 µl of a standard prep) were amplified using the commercial buffers with Mg concentration optimised, with 20–30 nmol of each primer, 55°C for annealing and 1 min for extension. For the data in Figure 2, the primers used for most BG genes were LP F1 (CCA GWT TCR CCC TYC CCT GGA GGA C), LP F2 (CTC CTG CCT TAT CTC RTG GCT CTG CAC), TM R1 (GAC ARA TGA CCC AMC SAG AWK TGT G) and TM R2 (CAC AGC CAG AGC CAC YKT CCA G), used in all four forward and reverse primer combinations. For BG2, the primers used were LP F1 and LP F2 as above, 43A R1 (GACAAATGACCCAGCCAGAGGAATTATG) and 43 R2 (CACAGCCAGAGCCACCTTCCAAG). For BG0, the primers used were LP F1 as above, CTBG F2 (CTC CTG GCT TAC CTC GTG GCT CTC AAC), CTBG R1 (CGA ATG ACG CAA ACA AAA GTG TGA G) and CTBG R2 (CCA CAG CCA GAG CCA CCT TCC AGG), used in all four forward and reverse primer combinations. Amplicons were isolated from TBE agarose gels using QIAquick Gel Extraction kit (QIAGEN 28706) and cloned using TOPO TA cloning kit (Invitrogen). Plasmid minipreps with right insert size after Eco RI digestion were sequenced using Big Dye Terminator reagent and an ABI automatic sequencer (Applied Biosystems).

### Reverse transcriptase-quantitative polymerase chain reaction (RT-qPCR) of cDNA from tissues (Figure S6)

Approximately 100 mg of spleen, liver, duodenum and bone marrow were collected from two 10 week old female C-B12 chickens from the Institute for Animal Health. Tissues were collected into 500 µl RNA-Later (Ambion) and stored at −20°C. RNA was isolated from ∼100 mg homogenised tissue using the Nucleospin RNA II kit (Machery-Nagel), and 1 µg RNA was converted to cDNA using oligo-dT primer and the Verso cDNA synthesis kit (Thermo Scientific), both according to manufacturer's instructions.

Forward and reverse primers for qPCR were TGTGCTGTGCAAGATGAT and TTCCAGGGATGGATGATG for BG4, TGTGTTGTGCAAGATGAC and GAAAAGCAATGATGACAA for BG7, TGTGCTGTGCAAGATGGT and ACGATCTGGGAAAAGGGG for BG10, and GGACGATCTGGGAAAAGA and TATGCAGAAGCTGTGGTGA for BG11. Targets were amplified over 40 cycles (initial enzyme activation 15 min 95°C, then 40 cycles of 15 s at 94°C, 30 s at 60°C and 30 s at 72°C) using 10 pmol each primer in AbsoluteBlue qPCR Mix (Thermo Scientific). Samples were compared to a 5-point standard curve, and normalised to cyclophilin A and a reference spleen sample. Fluorescence data were collected and analysed using an iCycler (BioRad) with subsequent analyses preformed in Microsoft Excel.

### Further PCR, cloning and sequencing

Amplifications in Cambridge were performed using a DNA Engine Tetrad 2 Peltier thermocycler (BioRad) using three commercial kits with buffers supplied and according to manufacturer's instructions.

For the experiment in Figure S5B, fragments were amplified from BACs P1(26)F6, 34 and P1(186)B6 [52] using 2 ng DNA, 0.6 mM each of the primers uc74 (CTCCTGCCTTATCTCRTGGCTCTGCAC), and uc76 (CACAGCCAGAGCCACYKTCCAG), with 1 U Velocity polymerase (Bioline BIO-21098) in 1×GC rich buffer containing 2 mM MgCl_2_ and 0.04 mM of each dNTP, with the annealing step being 2 min at 53°C.

For the experiment in Figure S5C, genomic DNA was isolated from erythrocytes using a salting-out procedure, as described [103]. Fragments were amplified from 2 ng line C-B12 genomic DNA using 0.2 mM each primer uc244 (F10: TTGGGGAAATAGTGTGACCG) with uc250 (R18: GGAGGGATCAGGAGGGAGC) or uc248 (R11: GGGGGGAAGAATTTAGGGAT) with 0.5 U recombinant Taq DNA polymerase (Invitrogen 10342020) in 1× Invitrogen PCR reaction buffer, 2 mM added MgCl_2_ and 0.25 mM of each dNTP. After initial denaturation, there were 5 cycles of 0.75 min at 95°C, 0.5 min at 60°C and 1.5 min at 72°C, followed by 30 cycles of 0.75 min at 95°C, 1 sec at 60°C and 1.5 min at 72°C, followed by a final extension step.

For the experiment in Figure S8, fragments were amplified from 1 µl (concentration unknown) line N genomic DNA using 0.4 mM of each primer [Pair 1: uc511 (BG0_P1F, TGCCCAGGGATGATTGTGAGGCT) and uc512 (BG0_P1R, TGCAGAACTGGGTGAGTCGTTCC); Pair 2: uc513 (BG0_P2F, TGCCCAGGGATGATTGTGAGGC) and uc514 (BG0_P2R, TGCAGAACTGGGTGAGTCGTTCCT); Pair 3: uc515 (BG0_P3F, GCCCAGGGATGATTGTGAGGCT) and uc516 (BG0_P3R, GCAGAACTGGGTGAGTCGTTCCT)], with 20 U Phusion polymerase (New England Biolabs #M0530S) in 1× HiFi buffer (containing 1.5 mM MgCl_2_), 1.5% polyethylene glycol and 0.04 mM of each dNTP. After initial denaturation, there were 5 cycles of 0.75 min at 95°C, 0.5 min at 59.8°C and 1.5 min at 72°C, followed by 30 cycles of 0.75 min at 95°C, 1 sec at 59.8°C and 1.5 min at 72°C, followed by a final extension step.

The amplicons were cloned using the CloneJET kit (Fermentas) following manufacturer's instructions, and sequenced using commercial fluorescent dye kits followed by capillary electrophoresis at the DNA Sequencing Facility of the Department of Biochemistry, University of Cambridge (www2.bioc.cam.ac.uk/~pflgroup/DNA_Facility).

Sequence data was analyzed using CLC DNA workbench (version 5.7.1, www.clcbio.com/). Alignments were performed using ClustalX (www.clustal.org/) and MAFFT (http://mafft.cbrc.jp/alignment/software/). Some phylogenetic trees were created using the neighbour joining (NJ) method implemented by ClustalX with 1000 bootstrap seeds. Other trees were created using a Bayesian approach implemented by MrBayes [104] (version 3.1.2, http://mrbayes.sourceforge.net/), with the GTR substitution model used with gamma-distributed rate variation across sites, and with the MCMC analysis using one chain, 20000 generations and sampling every 100 generations. Still other trees were created maximum parsimony (MP) phylogenies, using PAUP 4.0b10 for Unix [105] (www.paup.csit.fsu.edu/). AU and SH tests [106] on MP trees were performed in CONSEL version 0.2 (www.is.titech.ac.jp/~shimo/prog/consel/). Finally, other trees were created using neighbour network analysis implemented by SplitsTree4 [107], [108] (http://www.splitstree.org/), and tested for significance using a Phi test for recombination [109]. Phylogenetic trees were visualized using Dendroscope (http://ab.inf.uni-tuebingen.de/software/dendroscope/). After removal of gaps in the alignment of all 14 BG genes by G-blocks using default stringent parameters [110], [111], an automated partitioning analysis performed using SAGUARO [112] (www.sourceforge.net/projects/saguarogw/) gave cacti which were handled with PHYLIP version 1∶3.68-2 [113] from the Ubuntu repositories (www.launchpad.net/ubuntu/lucid/+source/phylip/1∶3.68-2). Dotplots were created with a wordsize of 150, using dottup from the EMBOSS package (version 6.1.0-5, http://emboss.sourceforge.net/). Helical wheels were created using pepwheel from the EMBOSS package (version 6.1.0-5, http://emboss.sourceforge.net/). Read-through exons were found using an in-house Visual BASIC script, and the ITIMs by an in-house PERL script, both written by J. Chattaway.

The 21 nucleotide repeats were grouped using an in-house rule based clustering algorithm (J. Chattaway). The clustering algorithm first created a distance matrix between the nucleotide sequences of all the 21 nucleotide repeats. Then, the algorithm created a list of 21 nucleotide repeats that were at least 80% identical to each repeat, and compared the lists and placed repeats that were similar to the same sets of repeats into the same group, again with an 80% identity threshold. The number of resulting groups was small enough to be checked manually; some groups were merged or split, and each group was given a number as a label, in order from the largest to the smallest group.

### Fibre-FISH (Figures 3 and 10)

The cell lines IS2 (MHC haplotype B2), UG5 (B13), TG15 (B15), IS19 (B19), and TG21 (B21) are reticuloendotheliosis virus (REV) transformed cell lines [114], [115], [116]. Primary B12 splenocytes were isolated from the spleen of a line C-B12 chicken (IAH, Compton; now rebranded as The Pirbright Institute) and stimulated with concanavalin A (ConA, Sigma) for 48 h. All cells were cultured at 37°C, 5% CO_2_ in RPMI 1640 supplemented with 10% FCS, L-glutamine and kanamycin (all from GIBCO/Invitrogen).

Fibre-FISH was performed at Sanger Institute as described [117], except the fibres were not treated with acetic acid and pepsin. Approximately 10 ng of each cosmid was used for amplification by the GenomePlex Whole Genome Amplification (WGA) 2 kit (Sigma) according to manufacturer's instructions, and probes created by amplification using a modified WGA3 kit (Sigma) [118], with cosmid c4.5 labelled with digoxigenin-11-dUTP (Roche); cG24 with biotin-16-dUTP (Roche), and cG43 with dinitrophenyl (DNP)-11-dUTP (Perkin-Elmer). Hybridisation was carried out as described [119] except that the probes were allowed to bind overnight. Detection and imaging was carried out as described [118] with the DNP-11-dUTP probe detected using a 1∶200 dilution of rabbit anti-DNP IgG and a 1∶200 dilution of AlexaFluor 488 donkey anti-rabbit IgG (both from Molecular Probes, part of Invitrogen).

## Supporting Information

Figure S1 The cosmids identified by screening with BG probes were characterised by double restriction enzyme digest and Southern blot, and could be organised into the previously described cluster I (now known as the BF-BL region), and two novel clusters named cluster V and cluster VI. Thick lines represent clusters, with restriction sites (C, Cla I; K, Kpn I; M, Mlu I; N, Nru I; n, Not I; S, Stu I; P, Pvu II; X, Xho I), open boxes indicating presumed BG genes based on hybridisation, and closed boxes indicating similar regions based on hydridisation (now known to contain kinesin and lectin-like genes). Thin lines represent individual cosmids, with dotted lines indicating sequence apparently from outside of the BG region found in chimeric cosmids, and arrows indicating further sequence. Bar indicates approximately 5 kb.
(PDF)

Figure S2 Alignment of the genomic sequence from all of the B12 BG genes, showing the locations of the coding sequence (yellow background), UTRs (green background) and the introns/intergenic sequence (white background). Residues which differ from a majority consensus sequence (not shown) are red; exons are highlighted in green; dashes are gaps included to maximise alignment. Each Genomic Fragment (GF) was cut 76 nucleotides after the predicted end of the 3'UTR, with the start of the 5' end just after the breakpoint of the previous 3' end (except for BG 10, preceded by the hypothetical protein, which was trimmed to match the 5' ends of the other genes). All features of the BG genes were initially predicted by reference to known BG cDNAs (as shown in Figure S5), and most have been validated by comparison to cDNAs from the particular gene. While not shown in this figure, the features of the C-type lectin-like, kinesin motor protein and hypothetical genes were established by reference to ESTs which span at least two exons and are over 90% identical to the genomic sequence (lectin: CD216101,CN217694; kinesin: BU471750, BU333462, BU251676, BU112055, BU108596, AM068009, AM063157, CV892843, CD217632, BU487705, BU330950, AJ442873, AJ398446, AJ398368, DT659179, DT655924, DR419449, CO507363, CN232628, CD217592, BU386334, BU354226, BM491516, BM490485, BM489471, BM425939, BG625424, AJ453838, AJ444817, DT657232, CD738123, CD728354, CD215461, BU199828, BU108016, BI393544, BI391565, AJ452709, AJ398223, AJ393429, BU328290, AJ399432, AJ393414, AJ393212 and the hypothetical protein: BU207055, BU209867, CN232293, BU374683, AJ442408, CB017432, AJ445065, AJ399199, BU451417, BU299883, BU357505, BU333629.
(PDF)

Figure S3 BG0 is identical in two haplotypes, and BG10 is most similar to the zipper protein, particularly in the cytoplasmic tail. A. Phylogenetic tree of nucleotide sequences. Alignments of B. BG10 with zipper protein, C. BG0 from red junglefowl and line CB. Exons alternately coloured grey and white.
(TIF)

Figure S4 Phylogenetic tree and alignment of sequences of coding region from “signal sequence to transmembrane” of B12 genes compared to the sequences amplified by the “universal primers” from cDNA of CB and CC chickens, as described in Figure 2.
(TIF)

Figure S5 PCR, cloning and sequencing links and orients cosmid clusters VI and V, and cosmid cluster V with the TRIM region, validating and extending the interpretations of the fibre-FISH experiments. A. Representation of five BAC clones with the right ends in the BF-BL region, extending to the left into the TRIM cluster (by sequence) or to the BG region by hybridisation indicated by black boxes. Figure from Ruby et al 2005, with permission from Springer 2013. B. Agarose gel of PCR products from three BACs using “universal primers” for BG genes, along with names of BG genes identified following cloning and sequencing. C. Sequence alignment of clones recovered after PCR from genomic DNA (C-B12 chicken) using primers on the right end of the cosmid cG24 from cluster VI and from the left end of cosmid cG43 from cluster V, compared to the ends of the cosmid clusters as determined by sequences of cG43, cG3 and cG24.
(TIFF)

Figure S6 The two haematopoietic BG genes BG7 and BG11 are well expressed in bone marrow but absent from liver, whereas the two tissue BG genes BG4 and BG10 are well expressed in duodenum and poorly expressed in bone marrow. RNA expression levels of BG7, BG11, BG4 and BG10 were compared by RT-qPCR. BG levels across tissues were normalised to the reference gene cyclophilin A, and relative fold difference in expression was calculated by normalising to spleen (spleen  = 1; fold values given above the bars; standard errors are from experimental triplicates on one plate). Data is representative of three experiments.
(TIF)

Figure S7 cDNAs isolated from B12 chickens and transfectants with B12 genes (black) validate the intron/exon structures predicted (blue) based on comparison with BG cDNAs from other haplotypes. A. BG0 (CTBG) cDNA clones from caecal tonsil (accession number KC955131), B. BG1 (8.5) clones after PCR from cDNA from L cells transfected with 8.5 gene (arrows indicate open reading frames) (accession numbers KC955132 to KC955136), C. and D. BG10 (13B, zipper protein-like) and BG11 (13A, 22E) clones after PCR from cDNA from L cells transfected with cosmids cG13 and cG22_2_.
(TIF)

Figure S8 There is only one 3′UTR exon for the BG0 gene in B21 chickens (line N). A. Dot plot analysis of the sequence around the 3′UTR from the WGS sequence shows duplication including the 3′UTR, with an insertion in the second copy. Three pairs of primers were designed just outside the apparent duplication and used for PCR from genomic DNA from a line N chicken, with the sizes expected for amplicons from the region with and without a duplication indicated below the dot plot. B. Picture of the amplification products separated by agarose gel electrophoresis, showing major amplified band below 3 kB compared to markers, as expected if the duplication is not found in the genome. C. Alignment between the WGS sequence 2.1 and the end sequences from the 3 kb band from PCR1.
(PDF)

Figure S9 The promoters of BG1 (8.5) and BG9 (F8) genes contain sequence derived from other genes. A. Sequence alignment of the BG1 promoter with the promoter of the neighbouring Blec (lectin-like) gene and with the consensus of other BG genes, showing that the upstream portion of the BG1 promoter is derived from the Blec promoter, followed by a region of unknown origin, finishing with the proximal region being similar to other BG genes. B. Sequence alignment of the BG9 promoter with the sequence of an upstream gene Loc425771 (hypothetical protein) found in the red junglefowl WGS sequence (positions 182878–183380) and in the sequence of BAC AB268588.1 (positions 29633–30097, sequence used in figure), which presumably became part of the BG9 promoter of the B12 haplotype upon deletion of the intervening sequences, as depicted in Figure 9. Interestingly, this novel sequence contains some tissue specific transcription factor binding sites (such as Gfi-1 described as the repressor induced by T-cell activation, Lyf1 described as important in the maturation of T-cells, and Nkx-2 found only in the brain and spinal cord), which correlate with the expression in T cells and brain found in Figure 2.
(TIFF)

Figure S10 Classification of cytoplasmic exons reveals that the downstream 80% follow the same pattern as the 3′UTR, but the upstream 20% do not. Each cytoplasmic exon of all BG genes from the B12 haplotype was given an individual identification number, a distance matrix for the sequences of all exons against all other exons was constructed, and then a rule-based algorithm was employed to identify groups of exons with sequences with at least 80% nucleotide identity. Visual inspection led to a few instances of splitting or merging groups, based on maximising shared nucleotides. A total of 57 groups were formed, with 18 groups having only one member and the remaining 39 groups having up to 39 members each. The figure shows the cytoplasmic exons (identified by group number) for each BG gene of the B12 haplotype, stacked for maximum alignment. A. Arrangement of all the cytoplasmic exons. B. Arrangement without those exons which are unique (that is, are in groups with only one member). Phylogenetic trees comparing the nucleic acid sequences of exons in common between each pair of BG genes from B12 haplotype for C. first 20% and D. last 80% of cytoplasmic tails.
(TIFF)

Figure S11 Six BG genes have the potential to conditionally express ITIM motifs, and all BG genes contain 1–5 introns which could be expressed as protein sequences without disrupting the continuity of the cytoplasmic tail. A. Depicted are ITIMs which are not preceded by a stop codon, introns which are in-frame with the two flanking cytoplasmic exons, in-frame stop codons and the resulting in-frame intron sequences without in-frame stop codons. B. The amino acid sequences of all predicted introns that contain an ITIM motif which is not preceded by a stop codon are shown as an alignment. ITIMs are highlighted in red. All numbers are relative to the start of the preceding heptad repeat. The sequences are named by the BG gene of origin and the ITIM number, starting with the 5' most ITIM which is not preceded by a stop codon. The amino acid sequence which is encoded by the cytoplasmic repeat exons is highlighted in blue. The first and last amino acids are omitted because they are partially encoded by the neighbouring exons, which may be alternatively spliced. The reading frame of the second short cytoplasmic exon can be altered by the ITIM containing intron, which can introduce in-frame stop codons.
(TIFF)

Figure S12 Trees created using a Bayesian approach are consistent with the trees created using a neighbour joining (NJ) approach. The trees are based on alignments of the promoter, 5'UTR, signal sequence, V-like region, transmembrane region or 3'UTR, and were created using MrBayes (version 3.1.2). The GTR substitution model was used with gamma-distributed rate variation across sites. The MCMC analysis used one chain, 20000 generations and was sampled every 100 generations. Nodes with a posterior probability less than 0.5 have been collapsed. The results of the Bayesian approach are consistent with the neighbour joining (NJ) approach, with the same groups found as in Figure 5.
(PNG)

Figure S13 Neither the AU test nor the SH test can support a single topology for the signal sequences, V-like region sequences or transmembrane sequences, in contrast to the analysis of the 5′UTR for which there are three very closely-related best trees and for 3′UTR for which there is a single best tree. The AU test cannot reject the null hypothesis for any V-like region, signal sequence or transmembrane region topology. The SH test supports multiple topologies; however these supported topologies have different structures. Topologies were created in PAUP using the MP method, then AU and SH tests were performed in CONSEL. For the signal sequence the 66 shortest topologies were compared, for the V-like region the 52 shortest topologies, and for the transmembrane region the 60 shortest topologies. The top ten topologies are shown for each region.
(TIFF)

Figure S14 Neighbour networks show a complex recombination pattern across the whole gene, but with topologies similar to trees made with other approaches. Neighbour networks of whole cDNA, promoter, 5'UTR, signal sequence, V-like region, transmembrane region, and 3'UTR were created with SplitsTree. The groups of genes remain the same as with neighbour joining or Bayesian approaches, save for the whole cDNA of BG10 which clusters with BG5, BG7 and BG10 rather than with BG2. The complex networked structure of the trees indicates substantial past recombination within the exons, which is compatible with the recombination assessed using the Phi test, as implemented in SplitsTree. Tests were performed on sequences of the whole gene (p = 0), promoter (p = 0.86), 5'UTR (p = 0.01), V-like region (p = 0.0017) and 3'UTR (p = 9.13e-12). Only the promoter sequences do not appear to be undergoing significant recombination.
(PDF)

Figure S15 Cacti produced by SAGUARO are compatible with the topologies of NJ trees in Figure 5. A. The genomic fragments detailed in Figure S2 have been aligned. Sequence is represented as a horizontal line, exons as black boxes joined by chevrons, and gaps as white space. B. The alignment was processed using Gblocks to ensure that orthologous bases were in the same column and to remove gapped positions. Retained portions of the alignment are identified using a black line, with white space representing portions of the alignment which were excluded. C. The Gblocks alignment was then processed with SAGUARO, and 33 cacti were produced, indicated by white boxes. D. Cacti which cover regions analysed in Figure 5 were selected for further analysis. A neighbour joining (NJ) tree was produced for each cactus using PHYLIP. Cactus 2 covers part of the promoter alignment, and shows same two groups of promoters as in Figure 5. Cactus 5 covers the first half of the 5'UTR, and shows the same two groups of genes as the 5' UTR in Figure 5. Cactus 19 covers part of the 5'UTR and part of the following intron. This tree has clustered most of the hematopoietic BG genes together, like the 5'UTR tree in Figure 5. Cactus 18 covers the V-like region and produces a similar tree to the V-like region tree in Figure 5. Cactus 36 covers the transmembrane region, and identifies the same groups as the transmembrane tree in Figure 5. E. The 3'UTR sequence has been split into five cacti: 15, 23, 28, 12 and 1 which are 53, 176, 78, 70 and 71 nucleotides in length, respectively. These sequences are short, and the underlying nucleotide sequence is very similar (Figure S2); therefore one or two unique SNPs can radically alter the position of a particular sequence in the tree. Cactus 15 has the same pattern and groups as the 3'UTR tree in Figure 5. The remainder of the 3'UTR cacti produce the same groups of genes as the 3'UTR tree in Figure 5, but each one has a few genes which have migrated elsewhere in the tree. Therefore the 3'UTR partitioning is broadly supported by the SAGUARO analysis, and overall the SAGUARO analysis is compatible with the NJ topologies presented in Figure 5.
(PDF)

Figure S16 The presence of hybrid BG genes in the B12 haplotype shows no obvious pattern, consistent with a random process of recombination in the centre of the genes. The 14 BG genes of the B12 haplotype (as in Figure 1) are depicted with coloured boxes illustrating presumed origin (as in Figure 5 but with the colours for the cytoplasmic tail and 3′UTR reversed).
(TIFF)

Figure S17 Identification of clusters of un-annotated BG genes in the WGS sequence (version 2.1) of the BQ (B21-like) haplotype. Upper panel, first red junglefowl cluster found in representation of the ENSEMBL analysis of a region assembled for chromosome 16. Lower panel, second red junglefowl cluster found in representation of the ENSEMBL analysis of a region of assembled contigs that are suspected but not shown to be part of chromosome 16. These representations taken from the ENSEMBL website show our location of BG genes as defined by a BLAST search with the 3′UTR of BG genes (red vertical lines labelled BLAT/BLAST hits), the location of two identified BG genes (dark red boxes labelled Ensembl), and location of most of the exons of the BG genes inappropriately linked (green boxes labelled Unigene EST clusters). In essence, the prediction programs failed to identify the 5′ end of the BG genes.
(PNG)

Figure S18 Phylogenetic tree comparing 3′UTR nucleotide sequences of all genes from the B12 haplotype and the genomic sequence of red junglefowl (RJF, BQ or B21-like haplotype, except BG1 from B21) showing that many genes from red junglefowl cluster 1 (purple) are the same as B12 cluster VI (yellow), but red junglefowl cluster 2 (teal) is not well-related to B12 cluster VI (orange). Genes from B12 and red junglefowl are named with the same convention: numbers begin with BG1 in the BF-BL region, and then rise in order of the location of the gene (or apparent location the case of red junglefowl cluster 2) compared to the BF-BL region. Note that RJF-BG5 and RJF-BG4 are quite different from all other BG genes.
(TIFF)
