# Supplementary figures and images for: Sequence of a Complete Chicken BG Haplotype Shows Dynamic Expansion and Contraction of Two Gene Lineages with Particular Expression Patterns
Source: PLoS Genet. 2014 Jun 5;10(6):e1004417. doi: 10.1371/journal.pgen.1004417 (PMC4046983; doi:10.1371/journal.pgen.1004417)

cluster I

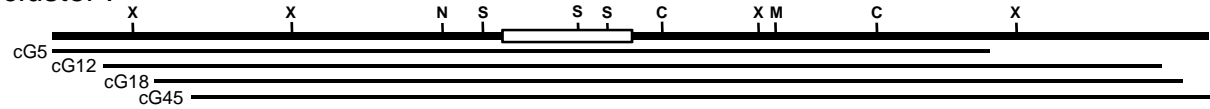

cluster V

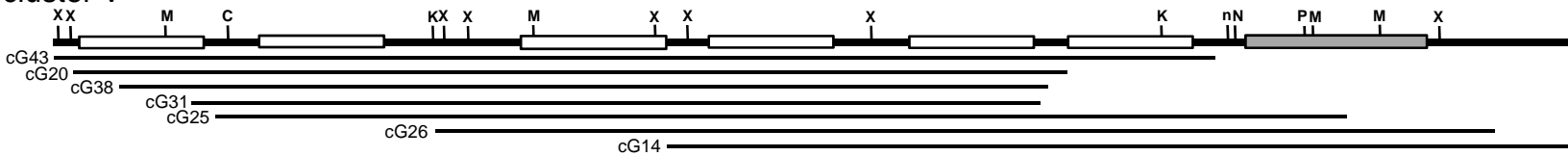

cluster VI

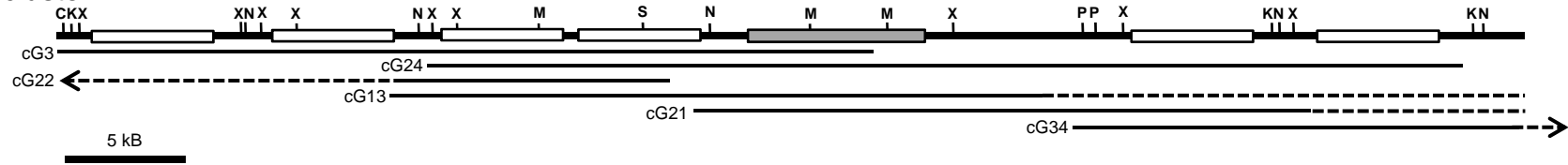

Supplement: Figure S1 — The cosmids identified by screening with BG probes were characterised by double restriction enzyme digest and Southern blot, and could be organised into the previously described cluster I (now known as the BF-BL region), and two novel clusters named cluster V and cluster VI. Thick lines represent clusters, with restriction sites (C, Cla I; K, Kpn I; M, Mlu I; N, Nru I; n, Not I; S, Stu I; P, Pvu II; X, Xho I), open boxes indicating presumed BG genes based on hybridisation, and closed boxes indicating similar regions based on hydridisation (now known to contain kinesin and lectin-like genes). Thin lines represent individual cosmids, with dotted lines indicating sequence apparently from outside of the BG region found in chimeric cosmids, and arrows indicating further sequence. Bar indicates approximately 5 kb. (PDF) [file pgen.1004417.s001.pdf]

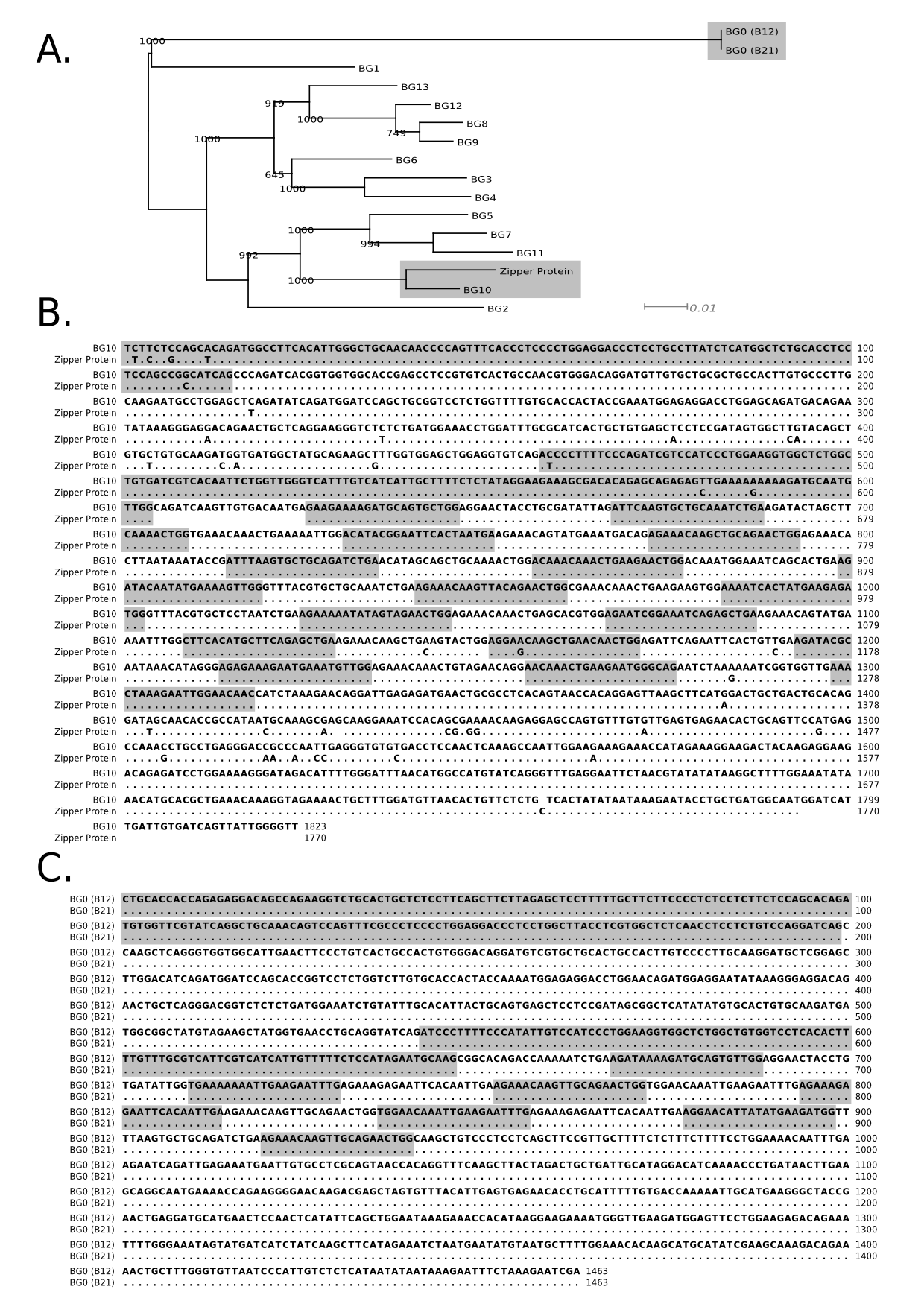

Supplement: Figure S3 — BG0 is identical in two haplotypes, and BG10 is most similar to the zipper protein, particularly in the cytoplasmic tail. A. Phylogenetic tree of nucleotide sequences. Alignments of B. BG10 with zipper protein, C. BG0 from red junglefowl and line CB. Exons alternately coloured grey and white. (TIF) [file pgen.1004417.s003.tif]

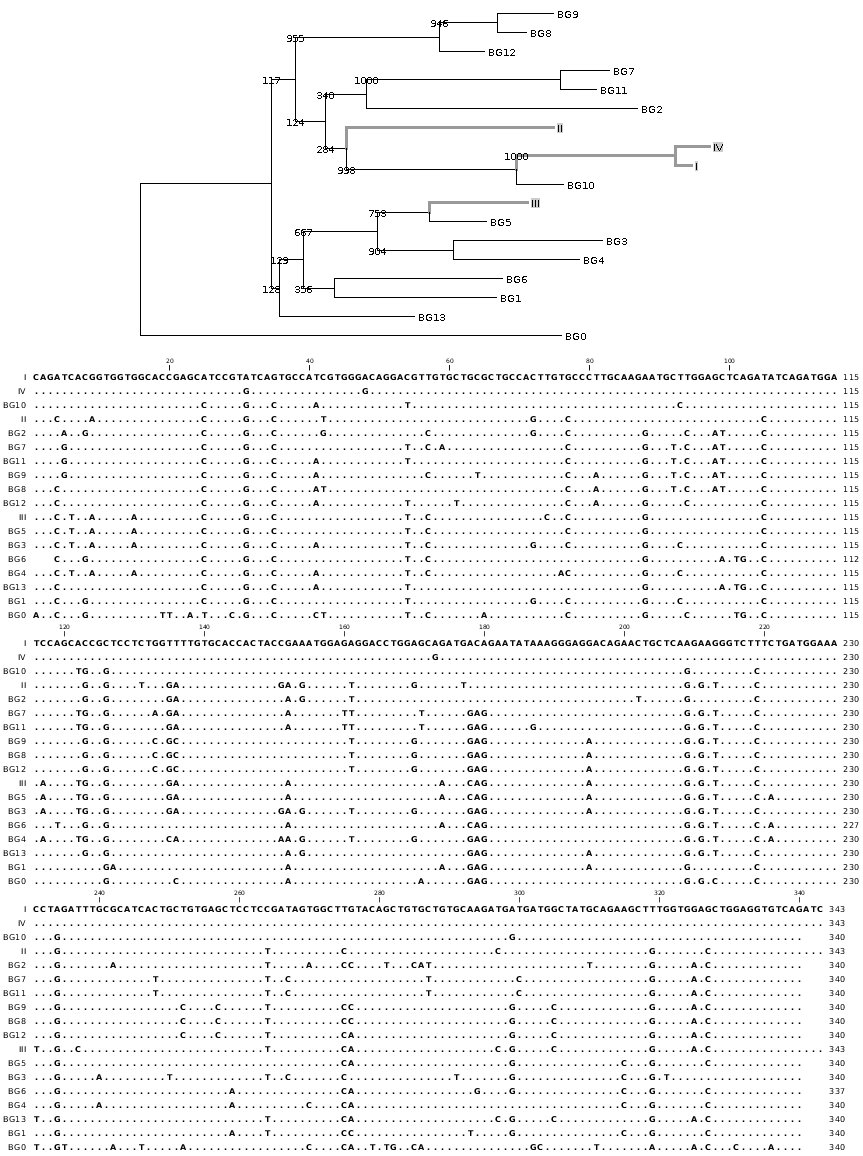

Supplement: Figure S4 — Phylogenetic tree and alignment of sequences of coding region from “signal sequence to transmembrane” of B12 genes compared to the sequences amplified by the “universal primers” from cDNA of CB and CC chickens, as described in Figure 2. (TIF) [file pgen.1004417.s004.tif]

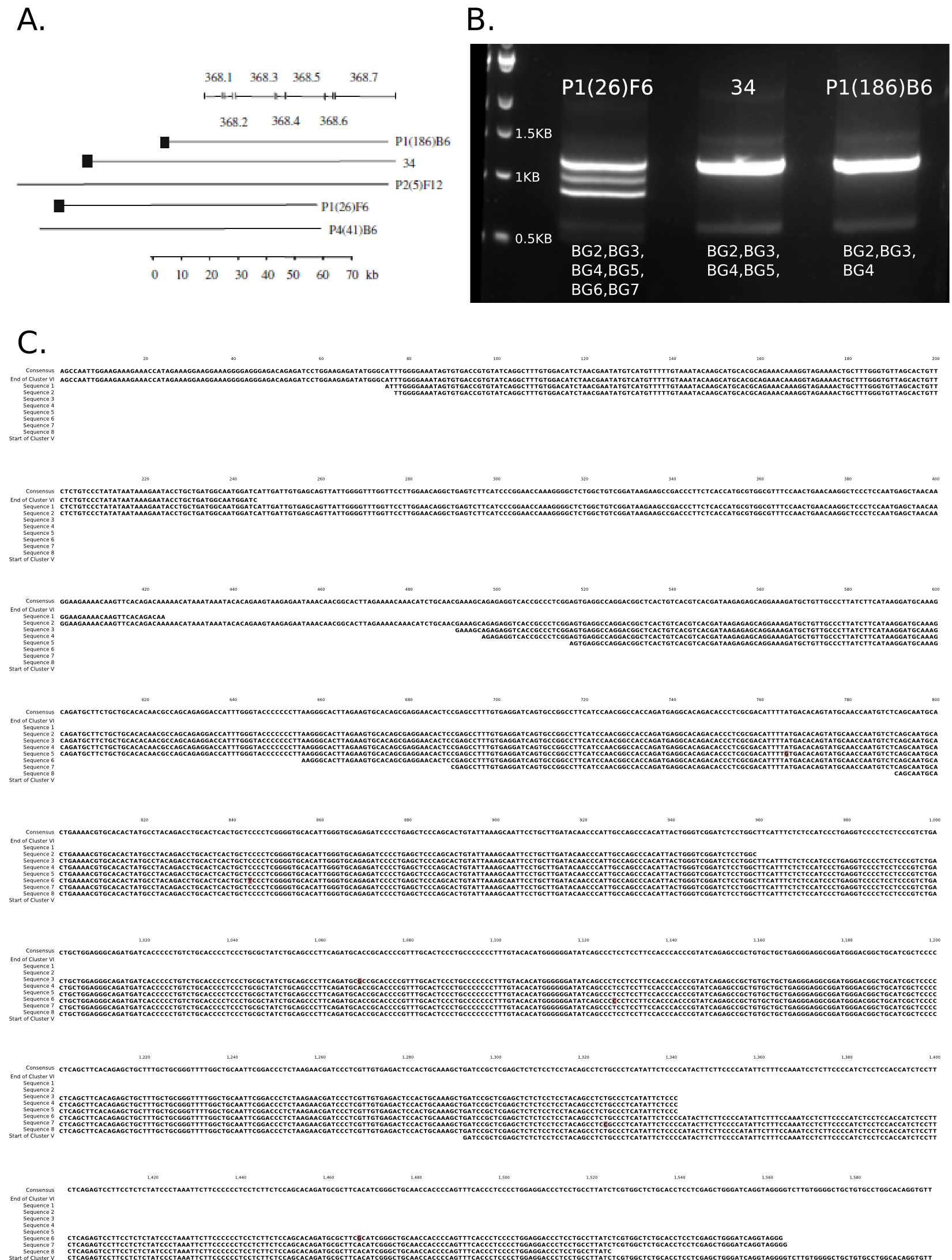

Supplement: Figure S5 — PCR, cloning and sequencing links and orients cosmid clusters VI and V, and cosmid cluster V with the TRIM region, validating and extending the interpretations of the fibre-FISH experiments. A. Representation of five BAC clones with the right ends in the BF-BL region, extending to the left into the TRIM cluster (by sequence) or to the BG region by hybridisation indicated by black boxes. Figure from Ruby et al 2005, with permission from Springer 2013. B. Agarose gel of PCR products from three BACs using “universal primers” for BG genes, along with names of BG genes identified following cloning and sequencing. C. Sequence alignment of clones recovered after PCR from genomic DNA (C-B12 chicken) using primers on the right end of the cosmid cG24 from cluster VI and from the left end of cosmid cG43 from cluster V, compared to the ends of the cosmid clusters as determined by sequences of cG43, cG3 and cG24. (TIFF) [file pgen.1004417.s005.tif]

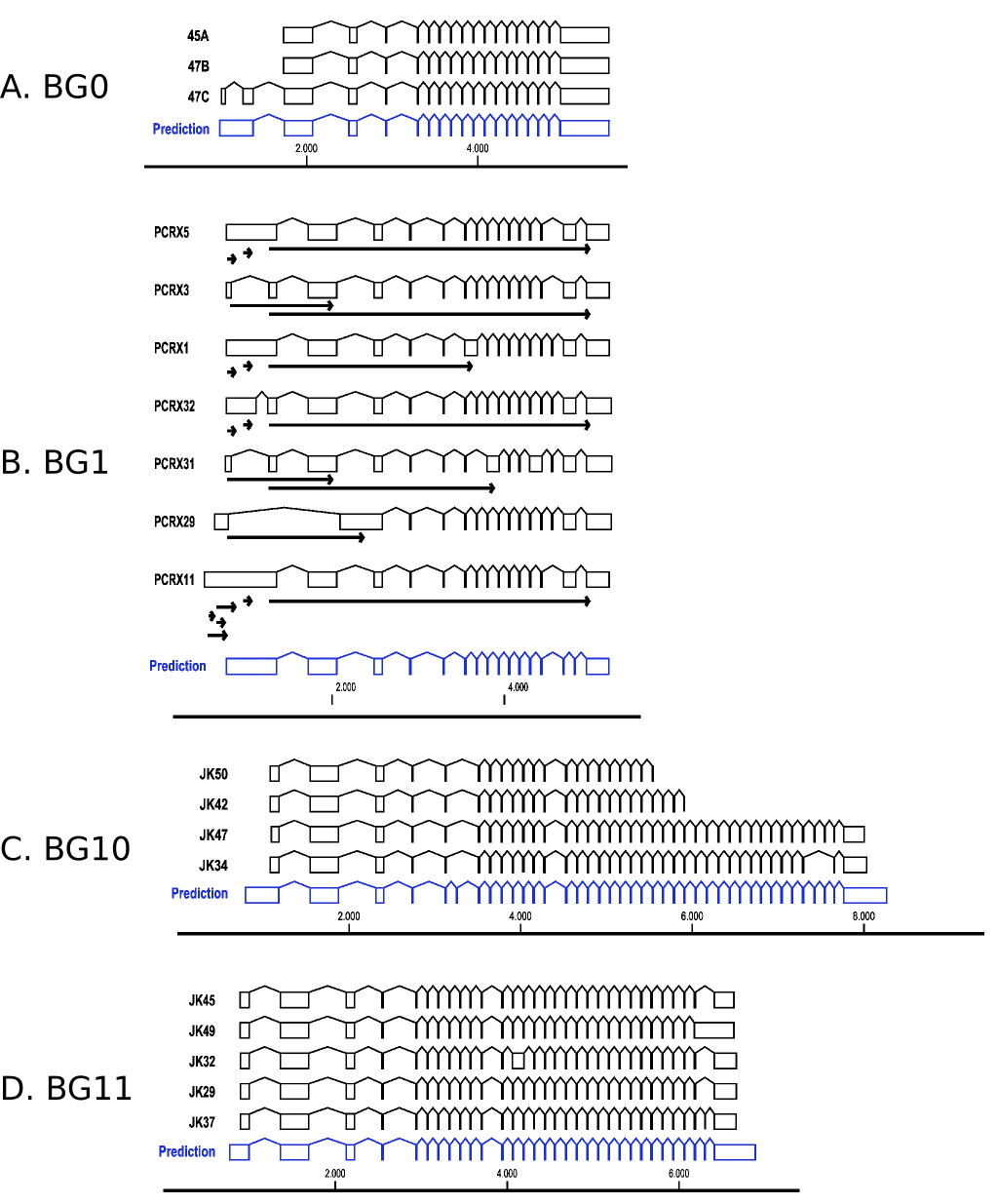

Supplement: Figure S7 — cDNAs isolated from B12 chickens and transfectants with B12 genes (black) validate the intron/exon structures predicted (blue) based on comparison with BG cDNAs from other haplotypes. A. BG0 (CTBG) cDNA clones from caecal tonsil (accession number KC955131), B. BG1 (8.5) clones after PCR from cDNA from L cells transfected with 8.5 gene (arrows indicate open reading frames) (accession numbers KC955132 to KC955136), C. and D. BG10 (13B, zipper protein-like) and BG11 (13A, 22E) clones after PCR from cDNA from L cells transfected with cosmids cG13 and cG222. (TIF) [file pgen.1004417.s007.tif]

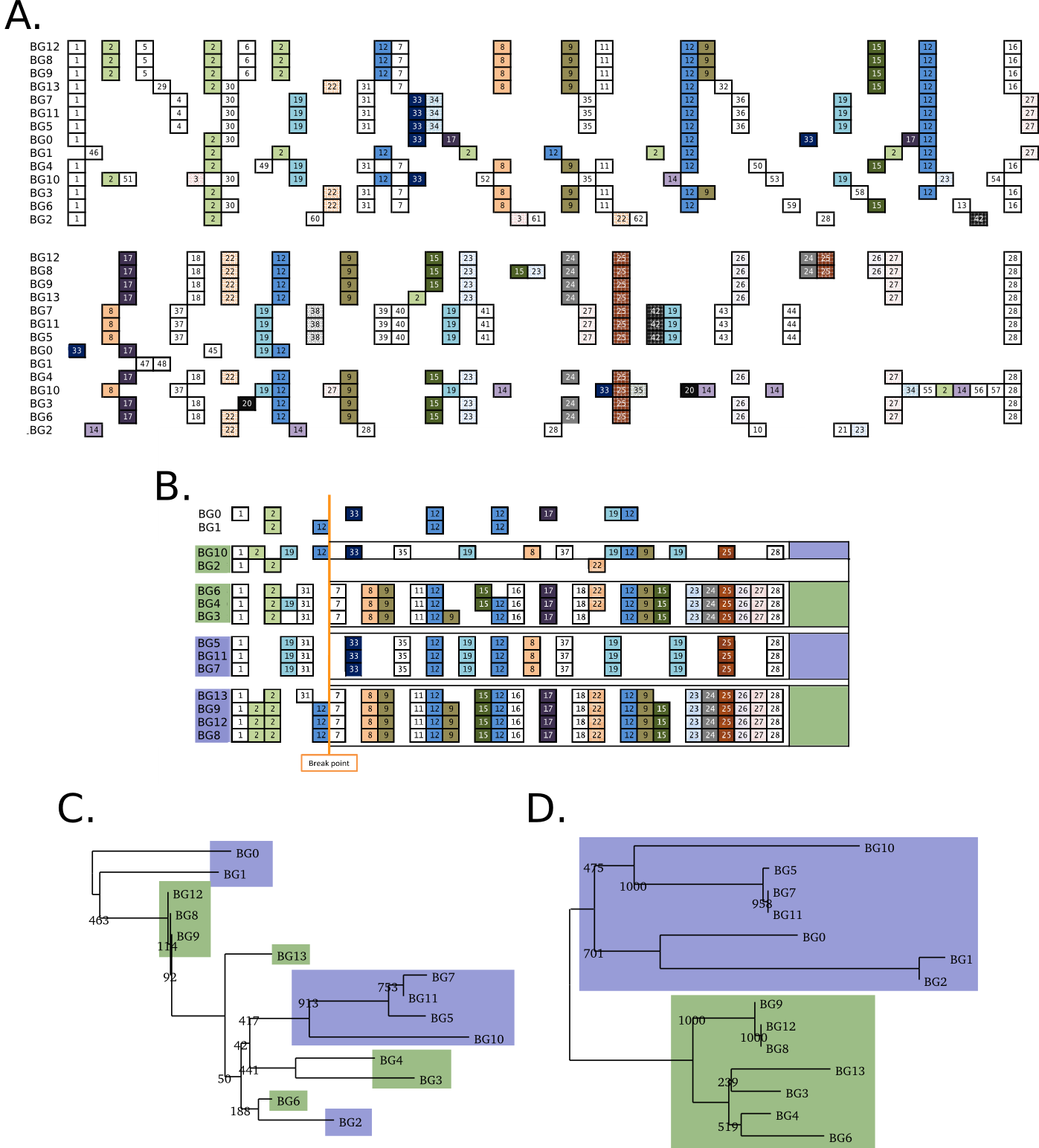

Supplement: Figure S10 — Classification of cytoplasmic exons reveals that the downstream 80% follow the same pattern as the 3′UTR, but the upstream 20% do not. Each cytoplasmic exon of all BG genes from the B12 haplotype was given an individual identification number, a distance matrix for the sequences of all exons against all other exons was constructed, and then a rule-based algorithm was employed to identify groups of exons with sequences with at least 80% nucleotide identity. Visual inspection led to a few instances of splitting or merging groups, based on maximising shared nucleotides. A total of 57 groups were formed, with 18 groups having only one member and the remaining 39 groups having up to 39 members each. The figure shows the cytoplasmic exons (identified by group number) for each BG gene of the B12 haplotype, stacked for maximum alignment. A. Arrangement of all the cytoplasmic exons. B. Arrangement without those exons which are unique (that is, are in groups with only one member). Phylogenetic trees comparing the nucleic acid sequences of exons in common between each pair of BG genes from B12 haplotype for C. first 20% and D. last 80% of cytoplasmic tails. (TIFF) [file pgen.1004417.s010.tif]

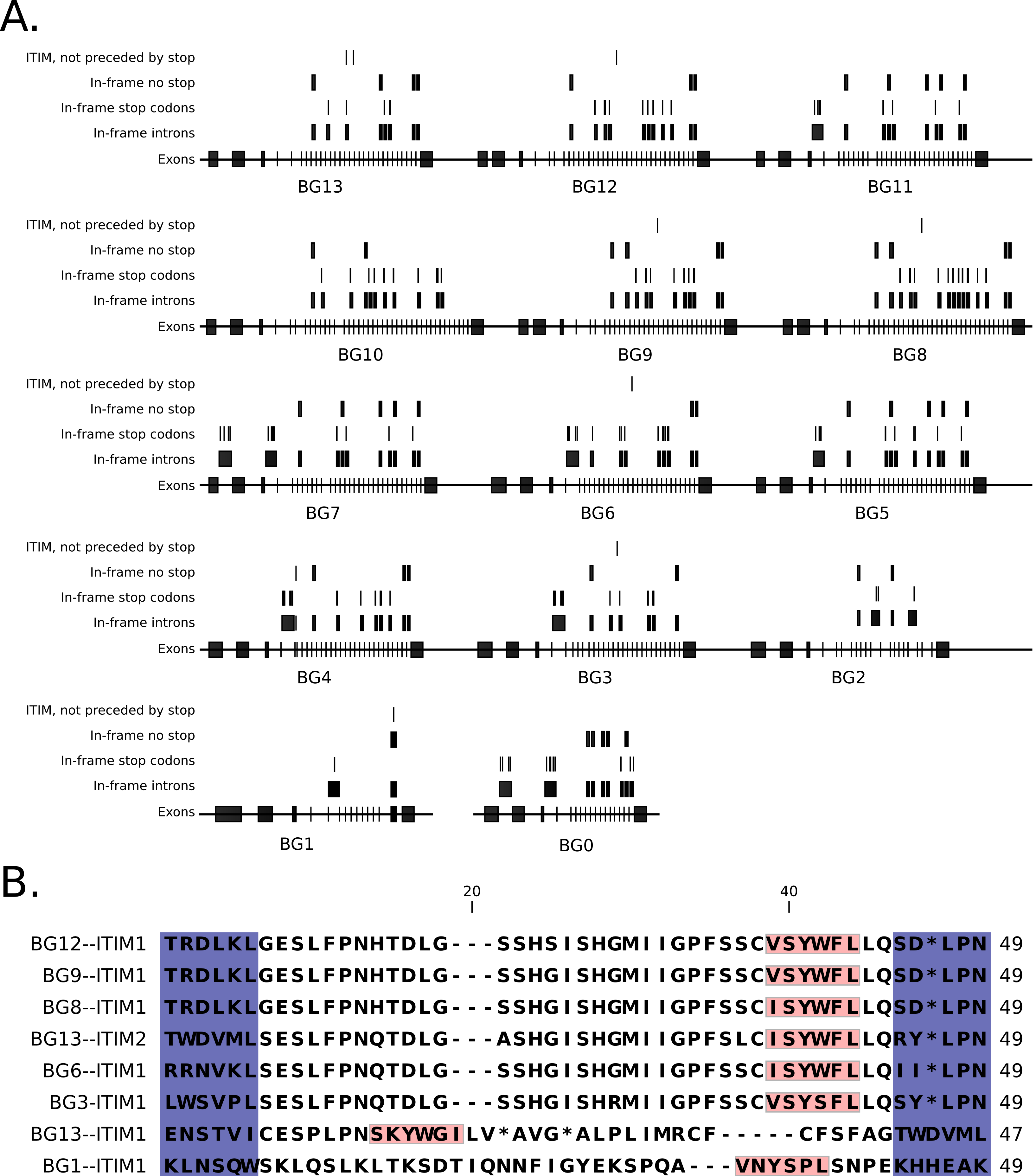

Supplement: Figure S11 — Six BG genes have the potential to conditionally express ITIM motifs, and all BG genes contain 1–5 introns which could be expressed as protein sequences without disrupting the continuity of the cytoplasmic tail. A. Depicted are ITIMs which are not preceded by a stop codon, introns which are in-frame with the two flanking cytoplasmic exons, in-frame stop codons and the resulting in-frame intron sequences without in-frame stop codons. B. The amino acid sequences of all predicted introns that contain an ITIM motif which is not preceded by a stop codon are shown as an alignment. ITIMs are highlighted in red. All numbers are relative to the start of the preceding heptad repeat. The sequences are named by the BG gene of origin and the ITIM number, starting with the 5' most ITIM which is not preceded by a stop codon. The amino acid sequence which is encoded by the cytoplasmic repeat exons is highlighted in blue. The first and last amino acids are omitted because they are partially encoded by the neighbouring exons, which may be alternatively spliced. The reading frame of the second short cytoplasmic exon can be altered by the ITIM containing intron, which can introduce in-frame stop codons. (TIFF) [file pgen.1004417.s011.tif]

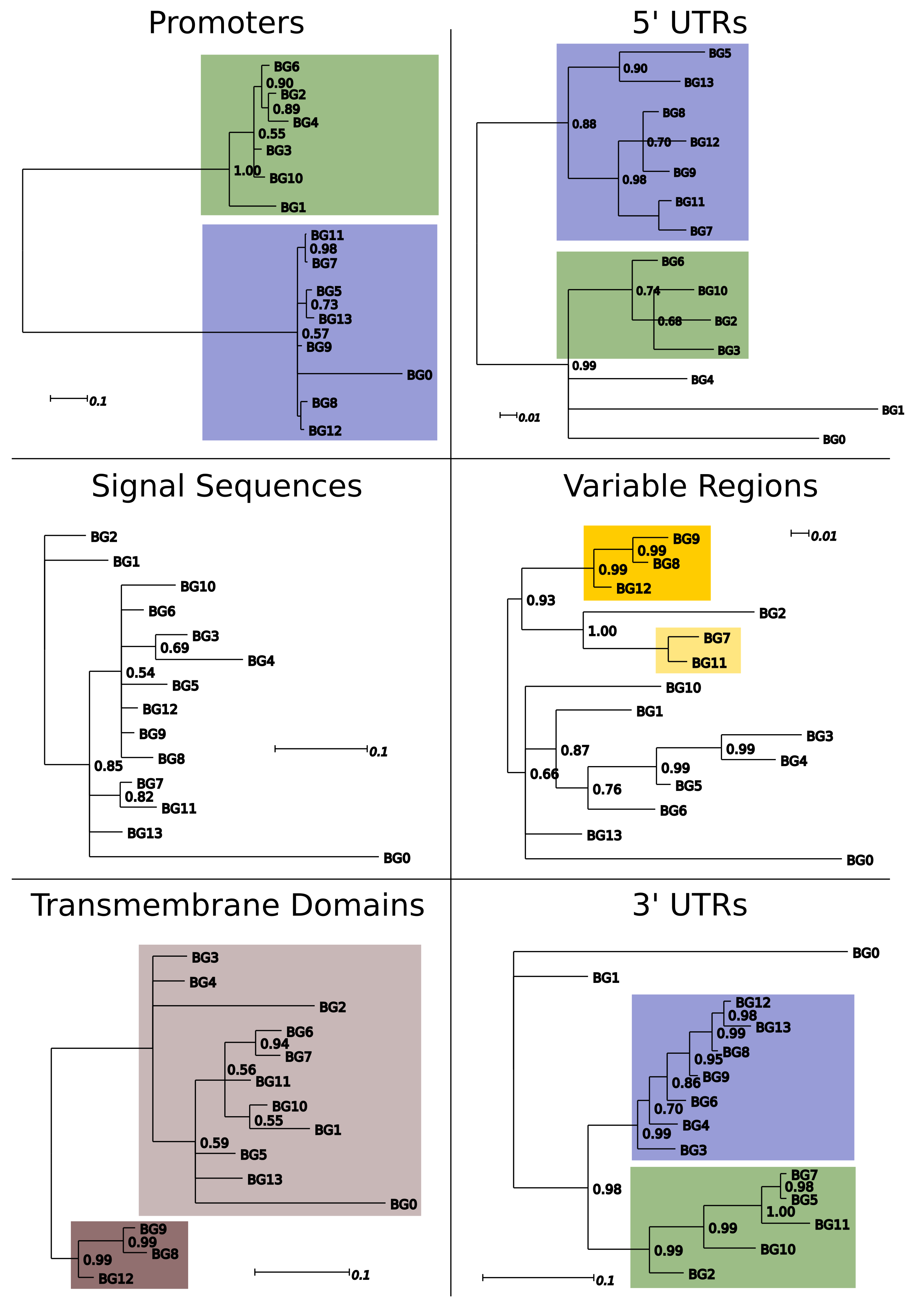

Supplement: Figure S12 — Trees created using a Bayesian approach are consistent with the trees created using a neighbour joining (NJ) approach. The trees are based on alignments of the promoter, 5'UTR, signal sequence, V-like region, transmembrane region or 3'UTR, and were created using MrBayes (version 3.1.2). The GTR substitution model was used with gamma-distributed rate variation across sites. The MCMC analysis used one chain, 20000 generations and was sampled every 100 generations. Nodes with a posterior probability less than 0.5 have been collapsed. The results of the Bayesian approach are consistent with the neighbour joining (NJ) approach, with the same groups found as in Figure 5. (PNG) [file pgen.1004417.s012.png]

# Whole cDNA

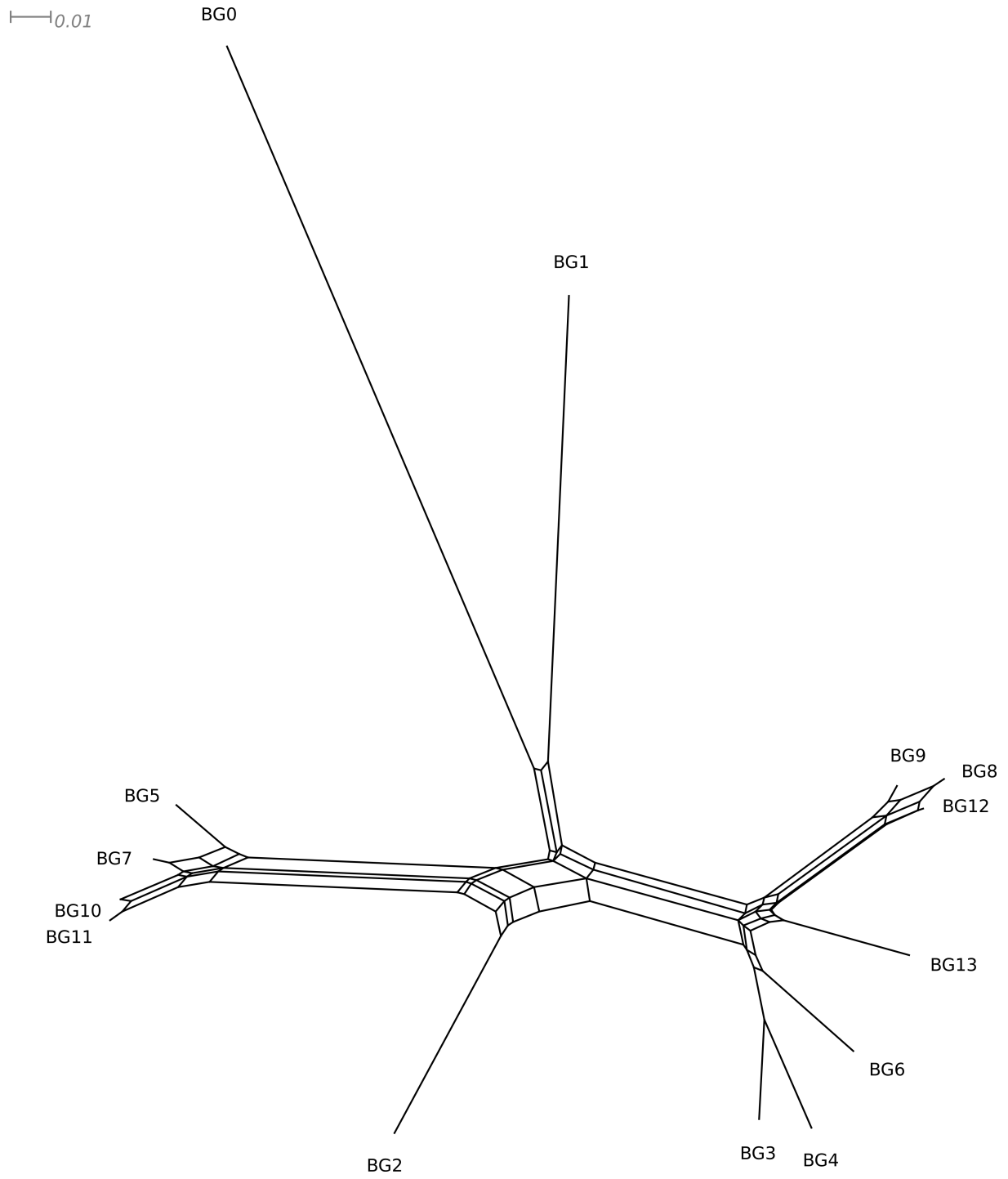

## Promoters

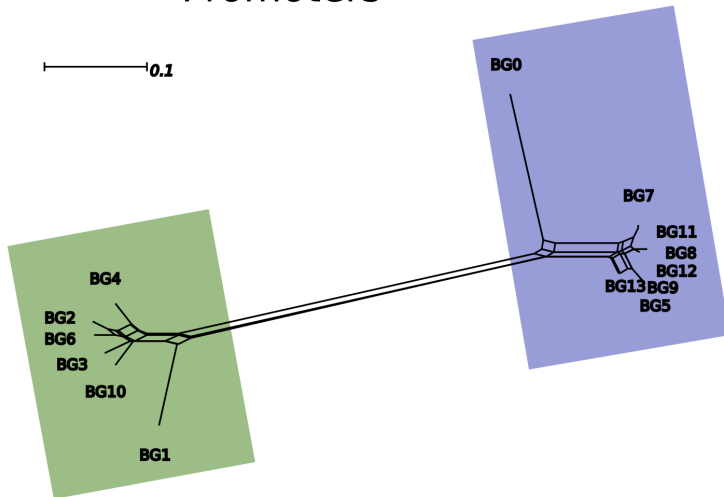

## 5' UTRs

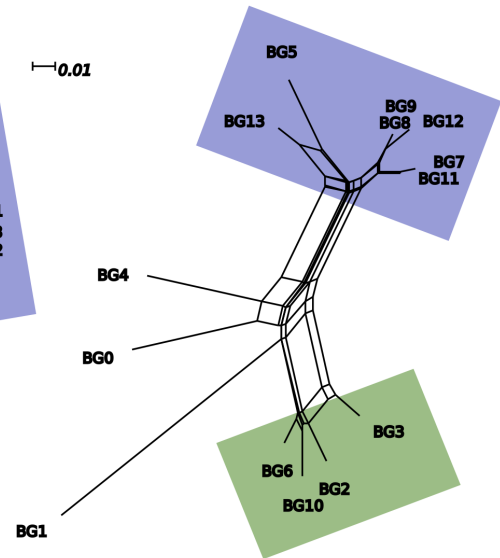

## Signal Sequences

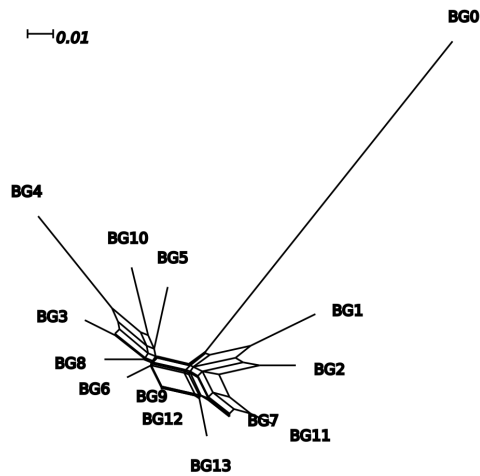

## Variable Regions

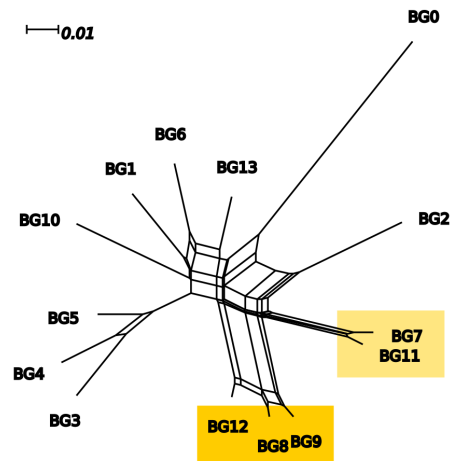

## Transmembrane Domains

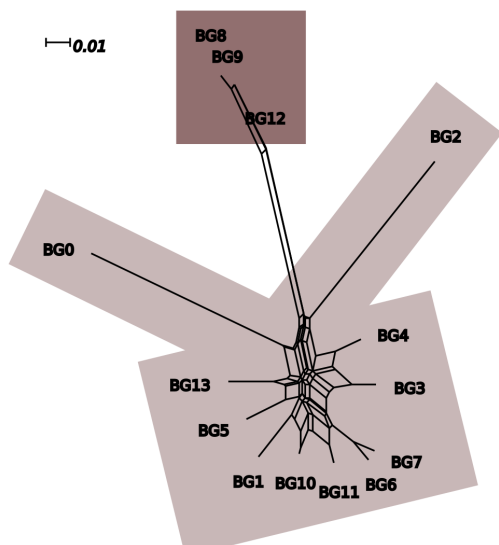

## 3' UTRs

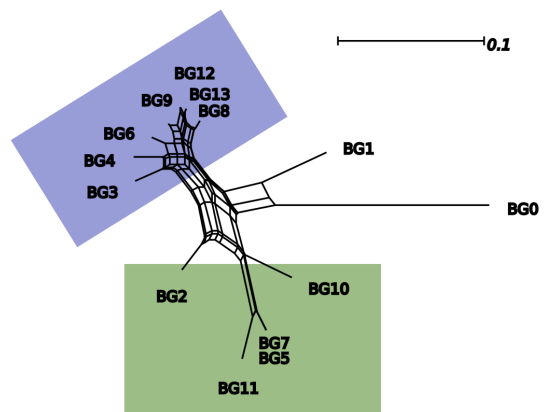

Supplement: Figure S14 — Neighbour networks show a complex recombination pattern across the whole gene, but with topologies similar to trees made with other approaches. Neighbour networks of whole cDNA, promoter, 5'UTR, signal sequence, V-like region, transmembrane region, and 3'UTR were created with SplitsTree. The groups of genes remain the same as with neighbour joining or Bayesian approaches, save for the whole cDNA of BG10 which clusters with BG5, BG7 and BG10 rather than with BG2. The complex networked structure of the trees indicates substantial past recombination within the exons, which is compatible with the recombination assessed using the Phi test, as implemented in SplitsTree. Tests were performed on sequences of the whole gene (p = 0), promoter (p = 0.86), 5'UTR (p = 0.01), V-like region (p = 0.0017) and 3'UTR (p = 9.13e-12). Only the promoter sequences do not appear to be undergoing significant recombination. (PDF) [file pgen.1004417.s014.pdf]

**A.**

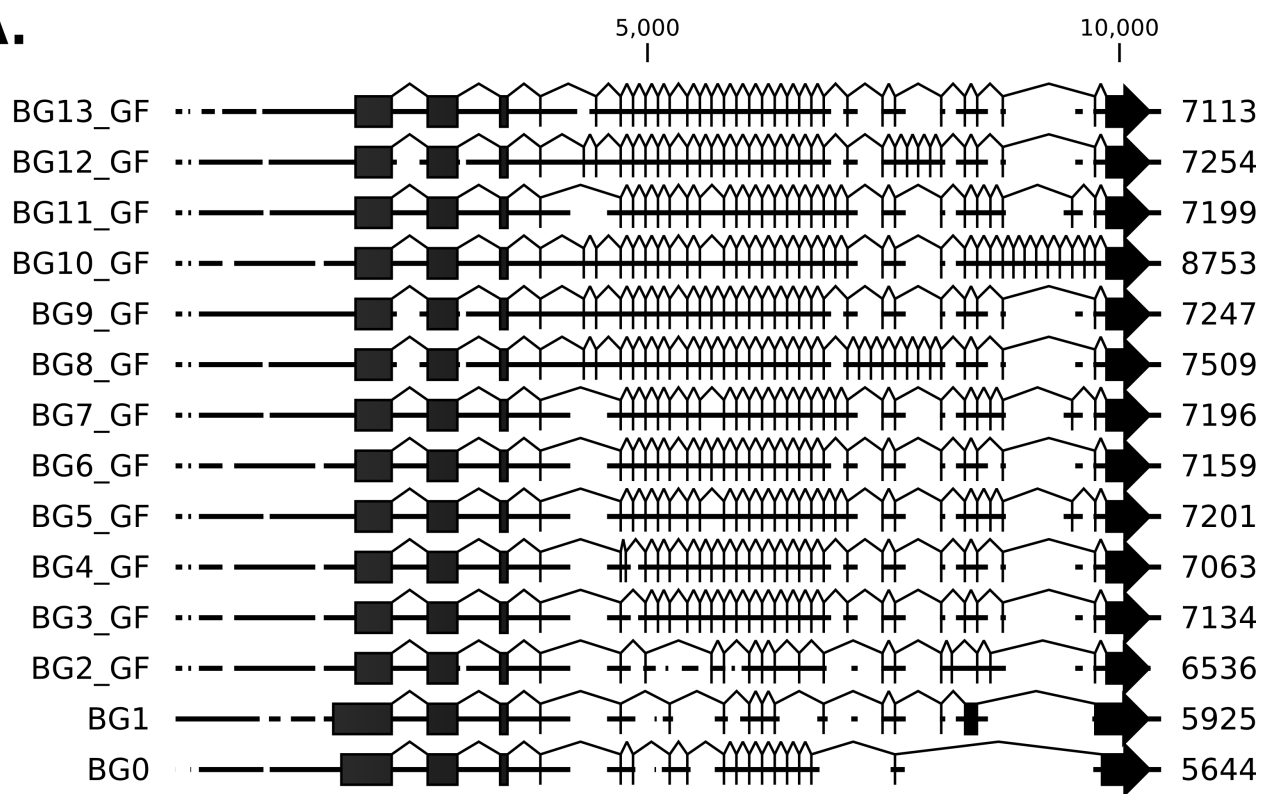

**B.**

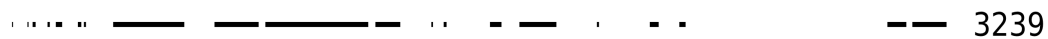

**C.**

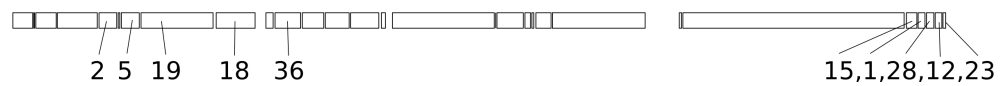

## D. Promotor

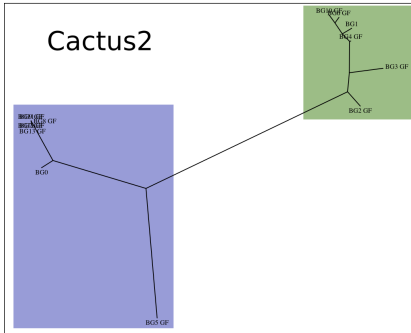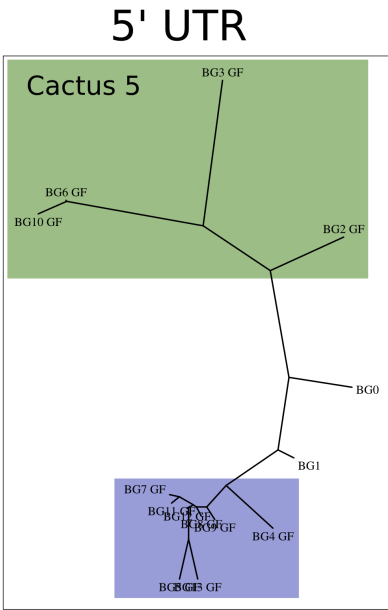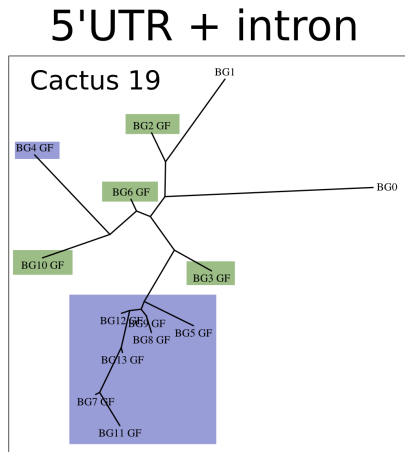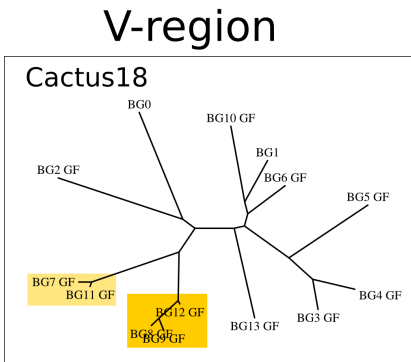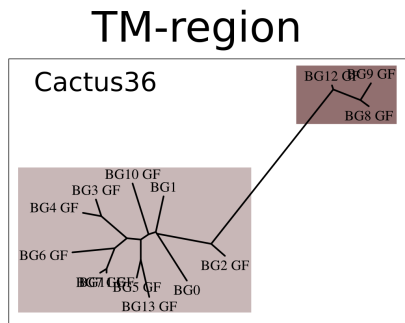

**E.**

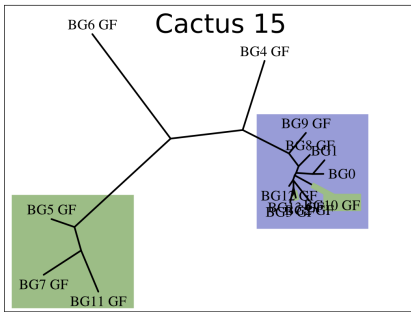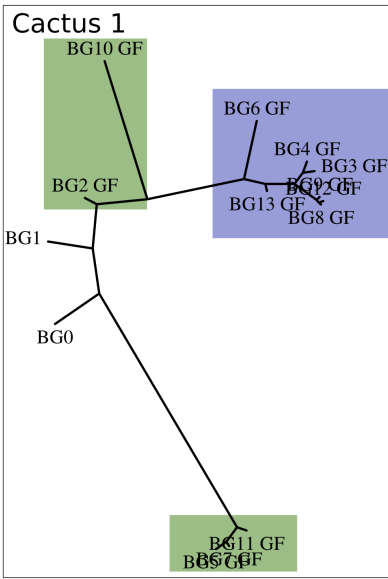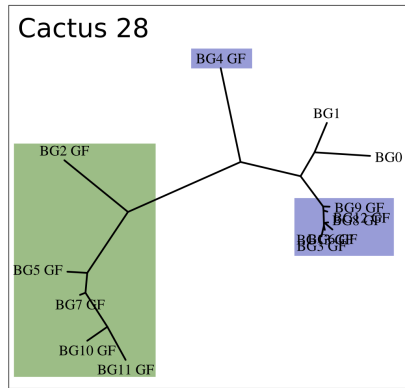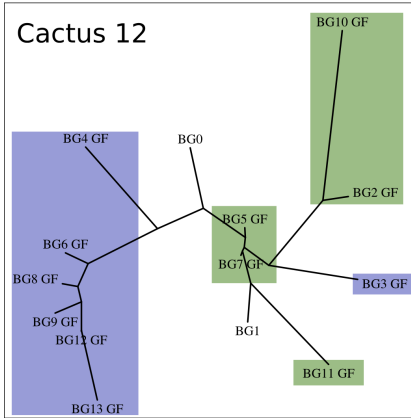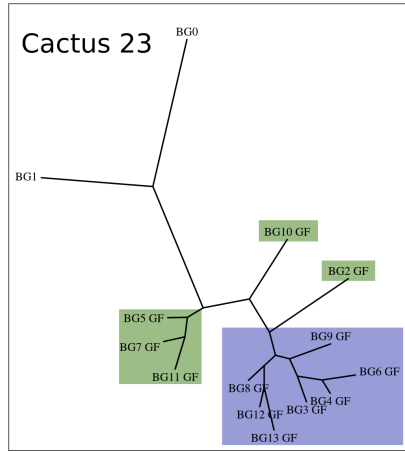

Supplement: Figure S15 — Cacti produced by SAGUARO are compatible with the topologies of NJ trees in Figure 5. A. The genomic fragments detailed in Figure S2 have been aligned. Sequence is represented as a horizontal line, exons as black boxes joined by chevrons, and gaps as white space. B. The alignment was processed using Gblocks to ensure that orthologous bases were in the same column and to remove gapped positions. Retained portions of the alignment are identified using a black line, with white space representing portions of the alignment which were excluded. C. The Gblocks alignment was then processed with SAGUARO, and 33 cacti were produced, indicated by white boxes. D. Cacti which cover regions analysed in Figure 5 were selected for further analysis. A neighbour joining (NJ) tree was produced for each cactus using PHYLIP. Cactus 2 covers part of the promoter alignment, and shows same two groups of promoters as in Figure 5. Cactus 5 covers the first half of the 5'UTR, and shows the same two groups of genes as the 5' UTR in Figure 5. Cactus 19 covers part of the 5'UTR and part of the following intron. This tree has clustered most of the hematopoietic BG genes together, like the 5'UTR tree in Figure 5. Cactus 18 covers the V-like region and produces a similar tree to the V-like region tree in Figure 5. Cactus 36 covers the transmembrane region, and identifies the same groups as the transmembrane tree in Figure 5. E. The 3'UTR sequence has been split into five cacti: 15, 23, 28, 12 and 1 which are 53, 176, 78, 70 and 71 nucleotides in length, respectively. These sequences are short, and the underlying nucleotide sequence is very similar (Figure S2); therefore one or two unique SNPs can radically alter the position of a particular sequence in the tree. Cactus 15 has the same pattern and groups as the 3'UTR tree in Figure 5. The remainder of the 3'UTR cacti produce the same groups of genes as the 3'UTR tree in Figure 5, but each one has a few genes which have migrated elsewhere in the tree [file pgen.1004417.s015.pdf]

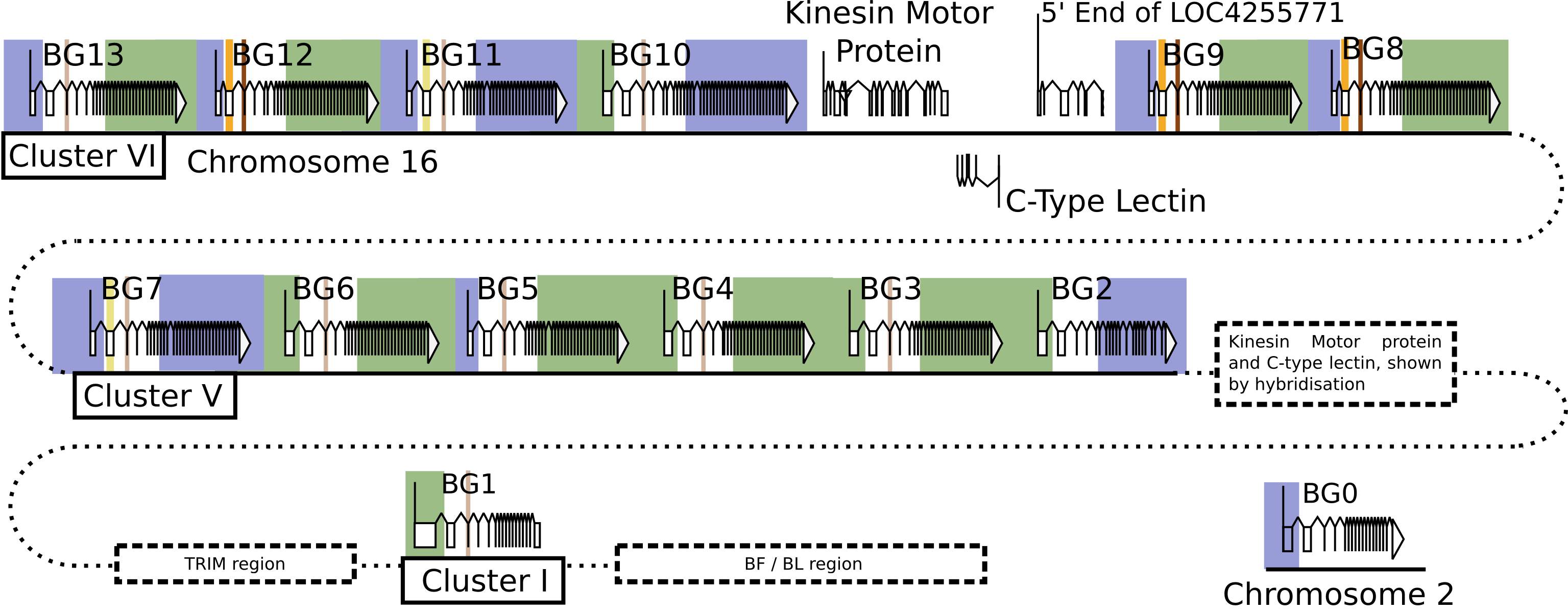

Supplement: Figure S16 — The presence of hybrid BG genes in the B12 haplotype shows no obvious pattern, consistent with a random process of recombination in the centre of the genes. The 14 BG genes of the B12 haplotype (as in Figure 1) are depicted with coloured boxes illustrating presumed origin (as in Figure 5 but with the colours for the cytoplasmic tail and 3′UTR reversed). (TIFF) [file pgen.1004417.s016.tif]

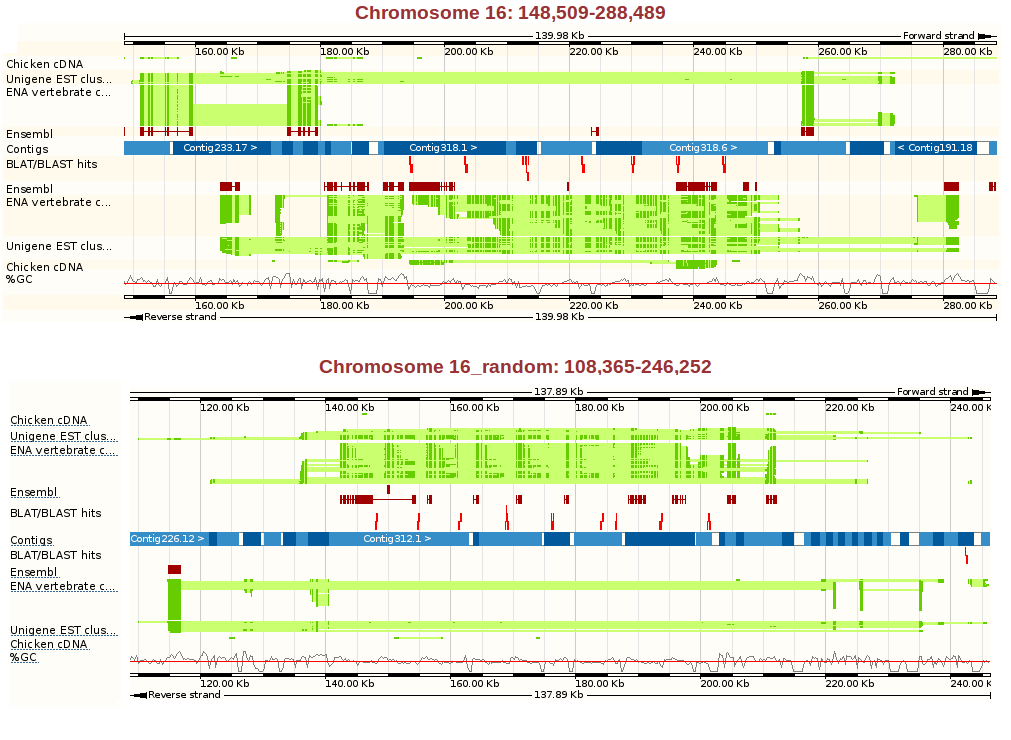

Supplement: Figure S17 — Identification of clusters of un-annotated BG genes in the WGS sequence (version 2.1) of the BQ (B21-like) haplotype. Upper panel, first red junglefowl cluster found in representation of the ENSEMBL analysis of a region assembled for chromosome 16. Lower panel, second red junglefowl cluster found in representation of the ENSEMBL analysis of a region of assembled contigs that are suspected but not shown to be part of chromosome 16. These representations taken from the ENSEMBL website show our location of BG genes as defined by a BLAST search with the 3′UTR of BG genes (red vertical lines labelled BLAT/BLAST hits), the location of two identified BG genes (dark red boxes labelled Ensembl), and location of most of the exons of the BG genes inappropriately linked (green boxes labelled Unigene EST clusters). In essence, the prediction programs failed to identify the 5′ end of the BG genes. (PNG) [file pgen.1004417.s017.png]

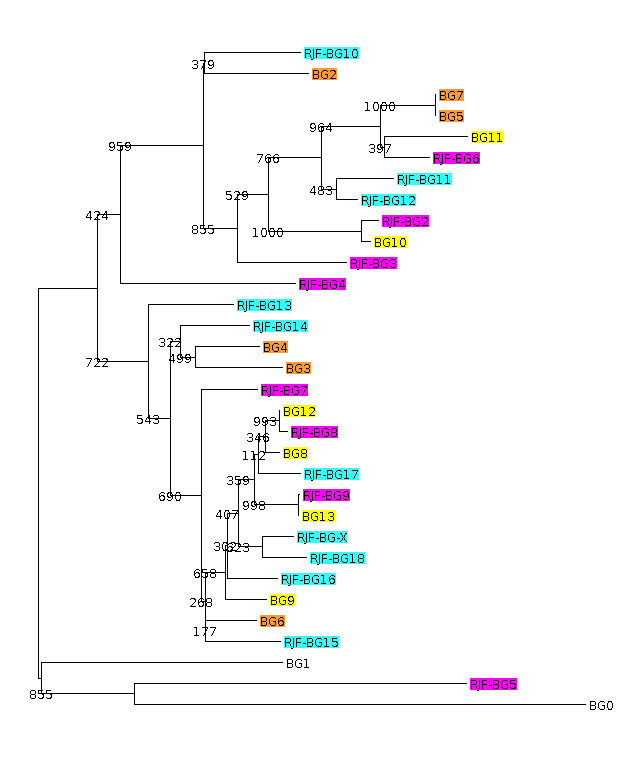

Supplement: Figure S18 — Phylogenetic tree comparing 3′UTR nucleotide sequences of all genes from the B12 haplotype and the genomic sequence of red junglefowl (RJF, BQ or B21-like haplotype, except BG1 from B21) showing that many genes from red junglefowl cluster 1 (purple) are the same as B12 cluster VI (yellow), but red junglefowl cluster 2 (teal) is not well-related to B12 cluster VI (orange). Genes from B12 and red junglefowl are named with the same convention: numbers begin with BG1 in the BF-BL region, and then rise in order of the location of the gene (or apparent location the case of red junglefowl cluster 2) compared to the BF-BL region. Note that RJF-BG5 and RJF-BG4 are quite different from all other BG genes. (TIFF) [file pgen.1004417.s018.tif]
