# Supplementary material for: Sequence of a Complete Chicken BG Haplotype Shows Dynamic Expansion and Contraction of Two Gene Lineages with Particular Expression Patterns
Source: PLoS Genet. 2014 Jun 5;10(6):e1004417. doi: 10.1371/journal.pgen.1004417 (PMC4046983; doi:10.1371/journal.pgen.1004417)

```
     BG13_GF G-------GGATCTGAATGAGGGGGTCCCATCTTGTTTAATATCCGT-------------ATCAATGACCT----------------------------------------------------------------GAATGAGGGGTCCCATCTTG------------ 71
     BG12_GF C-----------TCTCGGGTAAGAAGCCGAACCTCCTCACCATGCGT----------GGCGTT-----TCC----------------------------------------------------------------AACTGAACAAGGCTCCCTCC-ATGAGCTAA-- 74
     BG11_GF C-----------TGTCGGATAAGAAGCCGACCCTTCTCACCATGCAT----------GGCGTT-----TCC----------------------------------------------------------------AACTGAACAAGGCTCCCTCCAATGAGCTAA-- 75
     BG10_GF C---------TTTATCGGATAACAAGCTGACCCTTCTCACCAT--GT----------GGCGCTGCTATTCC----------------------------------------------------------------AGCACATCAAGGCTCTCTCCAGGAAGCTAA-- 80
      BG9_GF TGACGTGCTGAGTCCAGAGAGGGAGAAGGACCTCCAGAACCATCTCT----------GCTGTCAGTCAGGC----------------------------------------------------------------GGGTGGCGAAGGACTTTTCC-----ACCTG-- 86
      BG8_GF C-----------TGTCGGATAAGAAGCCGACCCTTCTCACCATGCGT----------GGAGTT-----TCC----------------------------------------------------------------AACTGAACAAGGCTCCCTCCAATGAGCTAA-- 75
      BG7_GF C-----------TGTCGGATAAGAAGCCGACCCTTCTCACCATGCGT----------GGCGTT-----TCC----------------------------------------------------------------AACTGAACAAGGCTCCCTCCAATGAGCTAA-- 75
      BG6_GF C---------TCTATCGGATAACAAGCTGACCCTTCTCACCA---------------GGCGCTGCTATTCC----------------------------------------------------------------AGCACAACAAGGCTCTCTCCAGGAAGCTAA-- 77
      BG5_GF C-----------TGTCGGATAAGAAGCCGACCCTTCTCACCATGCGT----------GGCGTT-----TCC----------------------------------------------------------------AACTGAACAAGGCTCCCTCCAATGAGCTAA-- 75
      BG4_GF C---------TCTATCGGATAACAAGCTGACCCTTCTCACCA---------------GGCGCTGCTATTCC----------------------------------------------------------------AGCACAACAAGGCTCTCTCCAGGAGGCTAA-- 77
      BG3_GF C---------TCTATCGGATAACAAGCTGACCCTTCTCACCA---------------GGCACTGCTATTCC----------------------------------------------------------------AGCAGAACGAGGCTCTCTCCAGGAAGCTAA-- 77
      BG2_GF C---------TCTATCGGATAACAAGCTGACCCTTCTCACCA---------------GGCACTGCTATTCC----------------------------------------------------------------AGCAGAACAAGGCTCTCTCCAGGAAGCTAA-- 77
 B12BG1Locus A-ACAGAACTGCGTCCAGACAGCGAGAAGCCCTTCTCCTTTATGTATCTTCATTACCAGAATTGCTTTTCCCCCCTTTTCTGGGATAGTTTCATTGCTGCATCATCTGCTTCATTTCCAATTCTCCTCCTTAACTGCCCCATGGAAACATCTTCCGGGGACAGATT 166
Annotated_BG0 G-----------------------------------------------------------------------------------------------------------------------------------------------------------CAGGCTTA-- 9

     BG13_GF ------------------------------------------------------------------------------------------------------------TTTAATATC-----------------CTTATTGATGATCTGAATGAGGGG--------- 104
     BG12_GF --------------------------------------------------------------------------------CAAGGAAGAAAACA-----AGTTCACAGACAAATACA-----------------TAAATAAATACACAGAAGTAAGAGAAT----AA 135
     BG11_GF --------------------------------------------------------------------------------CAAGGAAGAAAACA-----AGTTCACAGACAAATACA-----------------TAAATAAATACACAGAAGTAAGAGAAT----AA 136
     BG10_GF --------------------------------------------------------------------------------AAAGGAAGAAAAGA-----AATTAATAGGCAAAAAGAAAAAAATAAATAAAAAAAAAGAAAAACACAAAAACAAGAAAAATTT--TA 160
      BG9_GF --------------------------------------------------------------------------------CAAGCAGCCACAAA-----AATCCACACGCAAATCCC-----------------TCTTT---------------------------- 123
      BG8_GF --------------------------------------------------------------------------------CAAGGAAGAAAACA-----AGTTCACAGACAAATACA-----------------TAAATAAATACACAGAAGTAAGAGAAT----AA 136
      BG7_GF --------------------------------------------------------------------------------CAAGGAAGAAAACA-----AGTTCACAGACAAAAACA-----------------TAAATAAATACACAGAAGTAAGAGAAT----AA 136
      BG6_GF --------------------------------------------------------------------------------AAAGGAAGAAAATA-----AATTAATAGG-------------------------CAAAAAAATACACAAAACAAGAAAAATTT--TA 132
      BG5_GF --------------------------------------------------------------------------------CAAGGAAGAAAAGA-----AGTTCACAGACAAATACA-----------------TAAATAAATGCACAGAAGTAAGAGAAT----AA 136
      BG4_GF --------------------------------------------------------------------------------AAAGGAAGAAAAGA-----AAATAATAGG--------------------AAAAAAAAAAAAAAACACAGAACCAAGAAAATTT--TA 137
      BG3_GF --------------------------------------------------------------------------------AACGGAAGAAAAGA-----AATTAATAGG-------------------------CAAAAAAATACACAAAACCAAGAAAATTTAAAA 134
      BG2_GF --------------------------------------------------------------------------------AAAGGAAGAAAATA-----AATTAATAGG-------------------------CAAAAAAATACACAAAACAAGAAAAATTT--AA 132
 B12BG1Locus CATTGCTCCCCAGACTTTCGACACCTCTTCCTTTGCATATTGCTGGTTGTGCCCATCCCTTTGCAAACCCATTCCTTGCCAACGGAGGAGAGGA----------------------------------------TGGAGATGTGTGTGGTTCTGCACGGGGTCTGCG 293
Annotated_BG0 --------------------------------------------------------------------------------AGGAGAAAAAAATACTTACATTTTGCCGTTTAAAGGAGAATTAGGTAGTTGGTTCACTTAATCGTAGAATCACAGAACGGCCTGAGT 96

     BG13_GF ---TCCCATCTTGTTTAATATCCTTAT-CGATGAC---------------------CTGAATGAGGGGTCCCATCTTGTTTA------------------------------------------------------------------------------ATATCCT 168
     BG12_GF ACAACGGTACTTAGAAAACAAACATCTGCAATG-------------------------AAAGCAGAGAGGTCACCGCCCTCGGAGTGAGGCCAGGACG------------GCTCA--------------------------------CTGTCACGTCACGATA---- 229
     BG11_GF ACAACGGTACTTAGAAAACAAACATCTGC----AA---------------------TGAAAGCAGAGAGGTCACCGCCCTCGGAGTGAGGCCAGGACG------------GCTCA--------------------------------CTGTCACGTCACGATA---- 230
     BG10_GF AAAAGAATACTCC---AAAAAATCTAT------AA---------------------TTATTACAAAAATAATAACTTTTAAAAAA--CCACCAACGTT------------CCTCTCTGGGGGAGCACCAGTGACAGCCCTTTGTGTCCCATCGCAGTGCTATG---- 279
      BG9_GF ---CCCGTGCATGGGGAACATGGTTCTGTGCAGCC---------------------CCAAATTCCATGTTTTGTCCTCTCTGTGCACGGGGCAGCACC------------GCTCGGTTGCGTCACCCACACGTGTGGCTGTGCTGCTCAGCCATGTCACCATATCGT 254
      BG8_GF ACAACGGCACTTAGAAAACAAACATCTGCAATG-------------------------AAAGCAGAGAGGTCACCGCCCTCGGAGTGAGGCCAGGACG------------GCTCA--------------------------------CTGTCACGTCACGATA---- 230
      BG7_GF ACAACGGCACTTAGAAAACAAACATCTGC----AA---------------------TGAAAGCAGAGAGGTCACCGCCCTCGGAGTGAGGCCAGGACG------------GCTCA--------------------------------CTGTCACGTCACGATA---- 230
      BG6_GF AAAAGAATACTCC--AAAAAAATCTAT------AA---------------------TTATTACAAAAATAATAACTTAAAAAAAAACACACCAAACTT------------CCACCCTGGGAAAGCACCAACGACAGCCCTTTGTGTCCCATCGCGGTGCTATG---- 254
      BG5_GF ACAATGGTACTTAGAAAAAAAACATCTGC----AA---------------------TGAAAGCAGAGAGGTCTCTGCCCTCGGAGTGAGGCCAGGACG------------GCTCA--------------------------------CTGTCACGTCACGATA---- 230
      BG4_GF AAAAGAATACTCC---AAAAAATCTAG------AA---------------------TTACTACAAAAATAATAACTT-TAAAAAAACACACCAACCTT------------CCACCCTGGGGGAGCACCAACGACAGCCCTTTGTGTCCCATCGCGGTGCTATG---- 257
      BG3_GF AAAAGAATACTCCAAAAAAAAACCTGT------AA---------------------TTATTACAATAATAAC--TTT-AAAAAAAACACACCAACCTT------------CCTCACTGAGGGAGCACGACTGACAGCCCTTTGTGTCCCATCGCGGTGCTATG---- 255
      BG2_GF AAAAGAATACTCC-AAAAAAAACCTAT------AA---------------------TTATTATAATAATAAC----TTTAAAAAAACACACCAACCTT------------CCTCACTGAGGGAGCACCAACGACAGCCCTTTGTGTCCCATCGCGGTGCTATG---- 251
 B12BG1Locus GGAATAAGGGCTGCAAAAGACATCTCAGCAGCTGCTT-------------------TGCAGGCAGGCAGGATGCACTGAT------------------------------TCTTAGGGAGGGAGAGGTTA-----------------TCCGTGCGGGGATGCAGAGT 394
Annotated_BG0 TGAAAGGACCTC---AAAGATCATGTAGTTCCAACCCCCCTGCTGTGGGCAGGGCTGCAAACCAGCAGAGCAGGCTGCTGAGAGCCACATCCAGCCTGGCCTTGAACGCCTCCAGCAATGGGGCACCCAGAGTCTCTCTGGGCAACCTGTTCCAGTGCGTCACCAC 260

     BG13_GF TACTGATGACCTGAATGAGGGGGTCCCATCT---------------TGTTTAATATCCGTATCGATGACCTGAATGAGGGGTCCCATCTTGTATAATATCCTTACTGATGACCTGAATGAGGGACCCTCAGTAAGTCCGGAGGTGACACCA--AGCTGGAGGTGGT 318
     BG12_GF -------------------------------------AGAGAGCA-GGAAAGATGCTGTTGCCCTTATCT--------------------------------------TCATAAGGATGCAAAGCAGATGCTTCTGCTGCACACAACGCCAGCAGAGGACCATTTGG 320
     BG11_GF --------------------------------------AGAGAGCAGGAAAGATGCTGTTGCCCTTATCT--------------------------------------TCATAAGGATGCAAAGCAGATGCTTCTGCTGCACACAACACCAGCAGAGGACCATTTGG 321
     BG10_GF ------------------------------------------------------------------------------------------------------------------------AGAACAGCCACACACTTCAGAGTTTA---------CCCCACAGCAGA 317
      BG9_GF CACCATGAACCCAATGGAGCCAACTGCAGCATTGGCCAAAGGGCACTGTGGAACACGAGTGCTCTTCACTGCTGTTTGGGGCTGAGTTTTTGCACCACTCCTTCTCAACCATGTGGGCTCAAAGTTCACTCTTCTGCTGTAGAGAAGATGGGAAG-------CTCAA 414
      BG8_GF -------------------------------------AGAGAGCA-GGAAAGATGCTGTTGCCCTTATCT--------------------------------------TCATAAGGATGCAAAGCAGATGCTTCTGCTGCACACAACGCCAGCAGAGGACCATTTGG 321
      BG7_GF --------------------------------------AGAGAGCAGGAAAGATGCTGTTGCCCTTATCT--------------------------------------TCATAAGGATGCAAAGCAGATGCTTCTGCTGCACACAACGCCAGCAGAGGACCATTTGG 321
      BG6_GF ------------------------------------------------------------------------------------------------------------------------AGAACAGCCACACACTTCAGAGTTGA---------CCCCACAGCGGT 292
      BG5_GF --------------------------------------AGAGAGCAGGAAAGATGCTGTTGCCCTTATCT--------------------------------------TCATAAGGATGCAAAGCAGATGCTTCTGCTGCACACAACGCCAGCAGAGGACCATTTGG 321
      BG4_GF ------------------------------------------------------------------------------------------------------------------------AGAACAGCCACACACTTCAGAGCTGACCCTGTGATCCCCACAGTAGG 304
      BG3_GF ------------------------------------------------------------------------------------------------------------------------AGAACAGCCACACACTTCAGAGCTGACCCCGTGAGCCCCACAGTGGG 302
      BG2_GF ------------------------------------------------------------------------------------------------------------------------AGAACAGCCACACACTTCAGAGCTGATCCCGTTAGCCCCACAGTGGG 298
 B12BG1Locus TTGGGCTGACCTGGAAGATCAGCCCTGTGCAAAATGTGGGTGAGCAGCAGAAA--------------------AAAAAAAACATTCCAAAGGAGTGATTTCCCAGCAGGGAAAGGAGGGGAAGCTGCTTCCTGGTGCTGGCAGCAAAACTGCTGTGTCCTGATTGGG 541
Annotated_BG0 CCTGTGAGGCCGTGAGAACTTGCTGTCAAGTAAAGATAAGAGAGCAGGACAGATGTC-----------------------------------------TTGTTGATAAAGATAAGGAAGCAAAACAGATGCTTCAGCTCAACACAACACCAGCAGAGGCTGATTTAG 386

     BG13_GF GT------------GGATCCGCCTGAGG---GTGGGGAGGCCCTTCAGAGGGATCTCGATAAGCTGCATCGCTGGGCTGAAGCCAATG------------GGATGAGGTTCTCCCAGGCAGCACACAGGACCCGAGGAAATGGCCACGG------------GTTGTA 446
     BG12_GF GT------------ACACCCCCTTAAGG-------------------------GCACTTAGAAGTGCACAGCGAGGAACACTCCGAGC----CTTTGTGAGGATGA----------------GTGCCGGCCTTCATCCAACGGCCACCA------------GATGGG 418
     BG11_GF GT------------ACACCCCCTTAAGG-------------------------GCACTTAGAAGTGCACAGCGAGGAACACTCTGAGC----CTTTGTGAGGATGA----------------GTGCCGGCCTTCATCCAACGGCCACAA------------------ 413
     BG10_GF GGG-----------GACTCTCCTCCACAACCACCCCCATCACTCACATTGAATGCCCAAAGAA----------AAAACAGCCCAAGAGTTTCCTCCTGGTGCTTCAGCCTCGTGTGTTCCTCATTCTGCTGTGCTGATGGTGATCATTAACCCAACAGCTCATTAAC 463
      BG9_GF GC------------GATCGCTCTGTAGGCAAGAACACAAAATCCACACGGGCTGCTCTTCGTGCCACACCAATGGGACGGTCCCACACTTTGCTTCTTGGGTCTGACACACAGTCGTCACTTTTCTGGGTTTTAATACAAAAGGCATTA------------AAAAAA 557
      BG8_GF GT------------ACCCCCCCTTAAGG-------------------------GCACTTAGAAGTGCACAGCGCGGAACACTCCGAGC----CTTTGTGAGGATGA----------------GTGCCGGCCTTCATCCAACGGCCACCA------------GATGGG 419
      BG7_GF GT------------ACCCCCCCTTAAGG-------------------------GCACTTAGAAGTGCACAGCGAGGAACACTCCGAGC----CTTTGTGAGGATCA----------------GTGCCGGCCTTCATCCAACGGCCACCA------------------ 413
      BG6_GF GG------------GGACCTCCTCCACAACGACCCTCATCACTCACATAGAATGCCCAAAGTA----------AAAACAGCCCCAAAGGTTCCTCCTGGTGCTTCAGCCGCGTGTGTTCCTCATTCTGTTGTGCTGATGGTGACCATTAACCCAAGAGCTCATCGAT 437
      BG5_GF GT------------ACCCCCCCTTAAGG-------------------------GCACTTAGAAGTGCACAGCGCGGAACACTCCGAGC----CTTTGTGAGGATGA----------------GTGCCGGCCTTCATCCAACGGCCACCA------------------ 413
      BG4_GF AGGGAGGGGGGGGTGGACCTCCTCCACAACCACCCCCATCACTCACATTGAATGCCCAAAGGA----------AAAACAGCCCCAAAGGTTCCTCCTGGTGCTTCAGC--CGCGTGTTCCTCATTCTGCTGTGCTGATGGTGATCATTAACCCAAGAGCTCATCAAC 459
      BG3_GF G-------------GGACCTCCTCCACAACGACCCTCATCACTCACATTGAATGCCCAAAGGA----------AAAACAGCCCCAGATGTTCCTCCTGGTGCTTCAGCCACGTGTGTTCCTCATTCTGCTGTGCTGATGGTGATCATTAACCCAAGAGCTCATTGAC 446
      BG2_GF GGGGGGGGGGTGT-GGACCTCCCCCACAACCACCCTCATCACTCACATTGAATGCCCAAAGGA----------AAAACAGCCCCAAAGGTTCCTCCTGGTGCTTCAGC--CGCGTGTTCCTCATTCTGCTGTGCTGATGGTGATCATTAACCCAAGAGCTCATTGAC 452
 B12BG1Locus AACTCTGCTGGTGGGCATCTCCCCTCACCCCTTCCCCATCTGCCCTGCAGCATCCACGTGGAAATCCCCCATG--------------------------------------------TCACCATCATATCATCCCCTTCGTGATCATAA----GAAATTGGCTGGAG 660
Annotated_BG0 GCAGCCCCC-----CAACCCCCTTAAAG----------------------------GTTAGAAGTTCACAGCAAGGAAGACTTTGAGC----CTTTGTGAGGACAA----------------CTACTAGCCTTCATGCAACGACCACCA------------------ 482

     BG13_GF CCAGAGGAGGTTTAGGTTGGA---------------------------------------------------------------CAAAAGGA----------------------------------------AAAGCT-------------TTTCCTCTCAGAGAGT 497
     BG12_GF GCACAGACACCCTCGC------------------------GACATTTTATGACACA-------------------GTATGCAACCAA----------------------------------------------------------------TGTCTCAGCAATG--C 476
     BG11_GF ------------------GATGGGGCACAGACACCCTCGCGACATTTTATGACACA-------------------GTATGCAACCAATGTCTCAGC------------------------------------------------------------------AATGC 477
     BG10_GF CAGGTTATGGCTCAGGTGCGTGCTGCTGAACAAGCTTAGAGCCTAAAATGGTTCCA-------------------GCACACATCCCAGGGGACGGCCCTCCCCCTTTCACTCCCCGCCATTACAGCTCTCCTTAATCAGAGGAATACAGATTCCATGCACTGAGTGC 611
      BG9_GF AAAAACAAATTTTTGCTTGGAGTGAGCCAGACGGATCGGTATCGGATCACGCCTTC-------------------GTTTGCCACTGAGAGGA----------------------------------------GAGACGGGTGAGTGCTGCCCACCTCCACCATGGCT 665
      BG8_GF GCACAGAGACTCTCGC------------------------GACATTTTATGACACA-------------------GTATGCAACCAA----------------------------------------------------------------TGTCTCAGCAATG--C 477
      BG7_GF ------------------GATGAGGCACAGACACCCTCGCGACATTTTATGACACA-------------------GTATGCAACCAATGTCTCAGC------------------------------------------------------------------AATGC 477
      BG6_GF GAGGTTATGGCTCGGGTGGGTGCTGCTGAACAAGCTTGGAGCCTAAAATGGTTCCA-------------------GCACACATCCCAGGGTACTGCTCTCCCCCTTTCACTCCCCACCATTACAGCTCTCCTTAATCAGAGGAATACAGATTCCATGCACTGAGTGC 585
      BG5_GF ------------------GATGGGGCACAGACACCCTCACGACATTTTATGACACA-------------------GTATGCAACCAATGTCTCAGC------------------------------------------------------------------AATGT 477
      BG4_GF CAGGTTATGGCTCAGGTGCGTGCTGCTGAACAAGCTTGGAGCCTAAAATGGTTCCA-------------------GTACACATCCCAGGGGATGGCCCTCCCCCTTTCACTCCCCACCGTTACAGCTCTCCTTGATCAGAGGAATACAGATTCCATGCACTGAGTGC 607
      BG3_GF CAAATTATGGCTCAGGTGCGTGCTGCTGAACAAGCCTGGAGCCTAAAATGGTTCCA-------------------GCACACATCCCAGGGGACGGCCCTCCCCCTTTCACTCCCCACCATTACAGCTCTCCTTAATCAGAGGAATACAGATTCCATGCACTGAGTGC 594
      BG2_GF CAAATTATGGCTCAGGTGCGTGCTGCTGAACAAGCCTGGAGCCTAAAATGGTTCCA-------------------GCACACATCCCAGGGGACGGCCCTCCCCCTTTCGCTCCCCACCATTACAGCTGTCCTTAATAAGAGGAATCCAGATTCCATGCACTGAGTGC 600
 B12BG1Locus AAGAGTAGGCTTCAGGGGGACCTCACTGTGAATGCACTGGGGAAAATAACTCAACA--------------------------------------------------------------------------------------------------TTGCTCCAAGTGC 729
Annotated_BG0 ------------------GATGGGGCCCAGAGACCCTCCACACATTTTATGATGCATGCTCAGAAGGGTGGGATGATATGCAACCAATTTCTTGGA------------------------------------------------------------------AATGT 565

     BG13_GF G---------------------------------GTGAGGCGCTGGAATGGCTGCCCAGGGAGGTGG---------------TGGAGTCGCCATCCCGGGCAGTGTTGAAGAGGCGCCTGGATGAGGAG-------CTACGAGAGGTGGTTTGGTGCTTATGGAGC 609
     BG12_GF A---------------------------------CTGAATACATACACACTATGCCTACAGACCTGCACTCACTGCTCTCCTCAGGGTGCACATTGGGTGCAGAAATCCC-------CTGAG--------------CTCCAGGCACT------------------GT 571
     BG11_GF A---------------------------------CTGAAAACGTGCACACTATGCCTACAGACCTGCACTCACTGCTCCCCTCGGGGTGCACATTGGGTGCAGAGATCCC-------CTGAG--------------------------------CTCCCAGCACTGT 572
     BG10_GF A---------------------------------CCGAGCCATCGCCCACCTTCCCTACAAACAC----CTCCTGGTCCCCACAAACCCT-CACTGTGGGAAGAGGGGCT-------CTGGGGGGGTCACAGGGACAAACATTTAATAATTTCTGTATTAATGGTTG 733
      BG9_GF C---------------------------------CTCAGCCGTTTCCCACTTTGCACAGCGTTACCACTTTGCTGTGGCTCTCAGCCTTCCCATCTCTTGCAGGTGCCCCGACGCTGCTGGGACAAGAGGACGCCTTTCAAACAGTCACTCCCAGTGGAAGTGAAT 799
      BG8_GF A---------------------------------CTGAATACATGCACACTATGCCTACAGACCTGCACTCACTGCTCTCCTCAGGGTGCACATTGGGTGCAGAAATCCC-------CTGAG--------------TTCAGGCACT-------------------GT 572
      BG7_GF A---------------------------------CTGAAAACGTGCACACTATGCCTACAGACCTGCACTCACTGCTCCCCTCGGGGTGCACATTGGGTGCAGAGATCCC-------CTGAG--------------------------------CTCCCAGCACTGT 572
      BG6_GF A---------------------------------CTGAGCCATTGCCCACCTTCCCCACAACCC----CT--------------------CACTGTGGGAAGAGGGGCT-------CTGGGGGGATCACAGGGACAAACATTTAATAATTTCTGTATTAATGGTTG 688
      BG5_GF G---------------------------------CTGAATACATACACACTTGCCTACAGACCTGCACTCACTGCTCTCCTCAGGGTGCACATTGGGTGCAGAGACCCC-------CTGAG--------------------------------CTCCCAGCACTGT 572
      BG4_GF A---------------------------------CTGAGCCATCGCCCACCTTCCGCACAAACAC----CTCCTGGTCTCCACAAACCCCCCACTGTGGGAAGAGGGGCT-------CTGGGGGGATCACAGGGACAAACATTTAATAATTTCTGTAGTAATGGTTG 730
      BG3_GF A---------------------------------CTGAGCCATCGTCCACCTTCCCCACAAACAC----CT--------------------CACTGTGGGAAGAGGGGCT-------CTGGGGGGATCACAGGGACAAACATTTAATAATTTCTGTAGTAATGCTTT 697
      BG2_GF A---------------------------------CTGAGCCATCGCCCACCTTCCGCACAAACAC----CTCCTGGTCTCCACAAACCCCCCACTGTGGGAAGAGGGGCT-------CTGGGGGGATCACAGGGACAAACATTTAATAATTTCTGTAGTAATGGTTG 723
 B12BG1Locus AAGAGGATTTTAGGATAAAATTGACCATTCCAACATGAGAGGCTGCATGCTGGGATCACA------------------CCCTTGGAGTGCATCAAGTGTGGGAAGGGCTT--------------------------------------------------------- 821
Annotated_BG0 A---------------------------------ATTAATATGTATACACTATGCTTACAAATAAGCAGTTGCTGCTCCCCTTGGGGTGCATGTTCAGTGGAGAGATCCC-------CCGTG--------------------------------CACCCAGTGCTCT 660

     BG13_GF AGTGGGGATGGGAGGGCGGTTGGAACAGATGGTCTCGTAGGTCCTTTCCAACCTTGTGATTCTATGACTGTATAA---------CACACTGCCAGCC------------------CACGGTACCGG----------------------------------------- 708
     BG12_GF ATTAAA-------------------------------------------------GCAATTCCTGCTTGACACAA---------CCCATTGCCAGCC------------------CACGTTACCGG----------------------------------------- 621
     BG11_GF ATTAAA-------------------------------------------------GCAATTCCTGCTTGATACAA---------CCCATTGCCAGCC------------------CACGTTACTGG----------------------------------------- 622
     BG10_GF AGTAAC-----------------------------------------------TTAAAAATCCTTACTGATCAAA---------TAAACCTCTGCCCCTTGGGCATAGCTCAGAGCACGCTCATGG----------------------------------------- 803
      BG9_GF GGTAAA-------------------------------------------------TCCATTATCGCTTTCCTCCT---------CTCCCTGTC------------------------CACTGTTGG----------------------------------------- 843
      BG8_GF ATTAAA-------------------------------------------------GCAATTCCTGCTTGACACAA---------CCCATTGCCCGCC------------------CACGTTACCGG----------------------------------------- 622
      BG7_GF ATTAAA-------------------------------------------------GCAATTCCTGCTTGATACAA---------CCCATTGCCAGCC------------------CACATTACTGG----------------------------------------- 622
      BG6_GF ATTTAC-----------------------------------------------TTTAAAATCCTTACTGATTAAA---------TAAACTACCACCCCTTGGGCATAGCTCAGAGCAAGTTCATGG----------------------------------------- 758
      BG5_GF ATTAAA-------------------------------------------------GCAATTCCTGCTTGACACAA---------CCCATTGCCAGCC------------------CACATTACTGG----------------------------------------- 622
      BG4_GF AGTAAC-----------------------------------------------TTCAA-----------ATCAAA---------TAAATTACTG-CCCTTGTGCAAAGCTCAGAGCAAGCTCATGG----------------------------------------- 788
      BG3_GF ATTAAC-----------------------------------------------TTAAAAATCTGTACTGATCAAA---------TAAACTGCCACCCCTTGGGCATAGCTCAGAGCAAGCTCATGG----------------------------------------- 767
      BG2_GF ATTTAC-----------------------------------------------TTTAAAATCCTTACTGATTAAA---------TAAACTACCACCCCTTGGGCATAGCTCAGAGCAAGTTCATGG----------------------------------------- 793
 B12BG1Locus -----------------------------------------------------TTGGCTCTCCCCACTGCTCCAAGCCATATCCTTCACCACGTGCCCTTCAGAATAGCTCTGGCAGAATTACTGGTGCCTGTAACCTGTGGGATACAGGAGAAATTGAGAGAAATG 935
Annotated_BG0 ATTGAA-------------------------------------------------GCAATTCCTTCTTGACATAA---------CTCACTGGTGTGT------------------CAAGTTACTCT----------------------------------------- 710
```

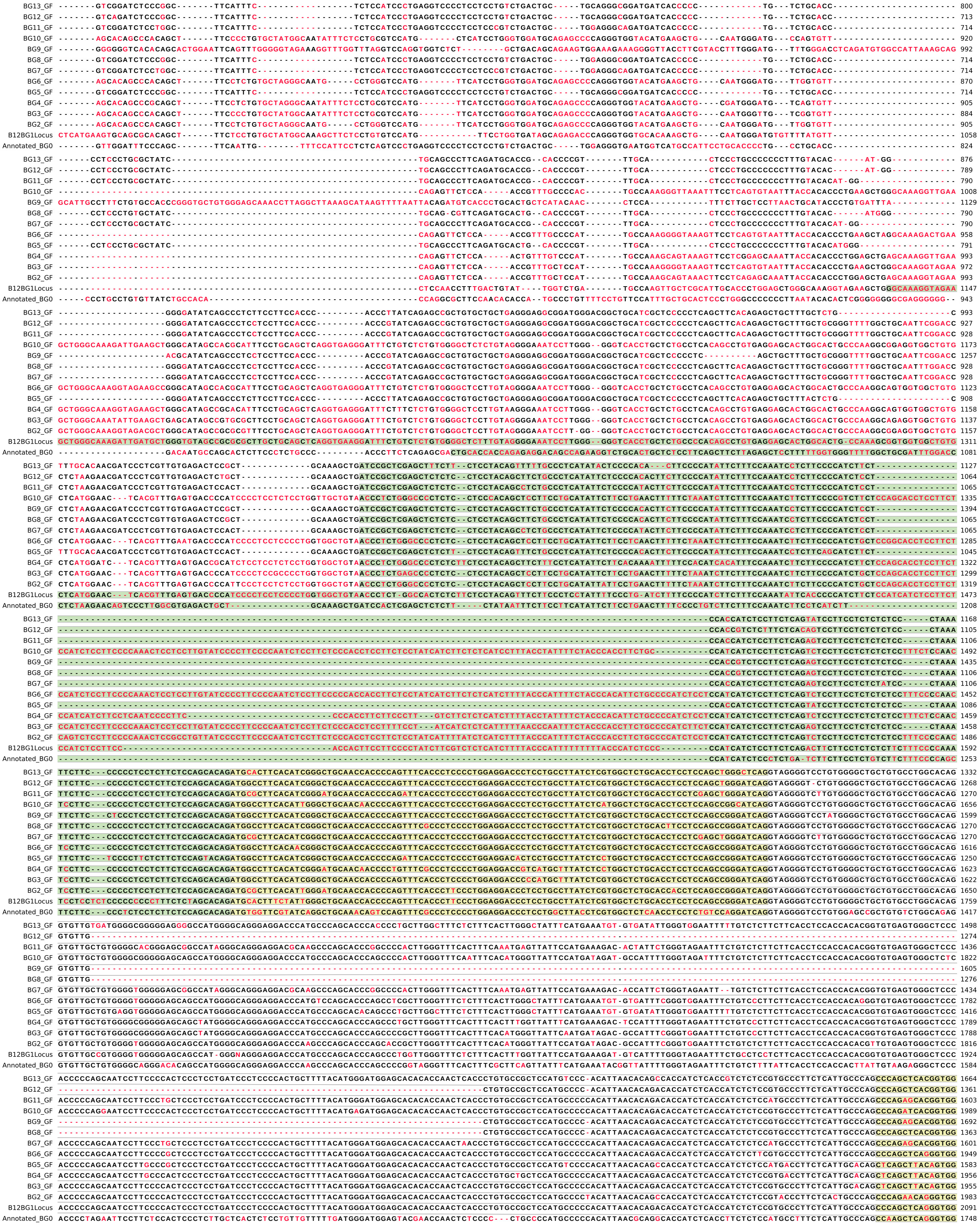

```
BG13_GF       TGGCACCGAGCCTCCGTGTCACTGCCAACGTGGGACAGGATGTTGTGCTGCGCTGCCACTTGTGCCCTTGCAAGGATGCTTGGAGATTGGACATCAGATGGATCCAGCAGCGGTCCTCTGGTTTTGTGCACCACTACCAAGATGGAGAGGACCTGGAGCAGATGGAG 1831
BG12_GF       TGGCACCGAGCCTCCGTGTCACTGCCAACGTGGGACAGGATGTTGTGTTGCGCTGCCACTTGTCCCCATGCAAGGATGCTCGGAGCTCAGACATCAGATGGATCCAGCAGCGGTCCTCTCGGCTTGTGCACCACTACCGAAATGGAGTGGACCTGGGGCAGATGGAG 1528
BG11_GF       TGGCACCGAGCCTCCGTGTCACTGCCAACGTGGGACAGGATGTTGTGCTGCGCTGCCACTTGTCCCCTTGCAAGGATGTTCGGAATTCAGACATCAGATGGATCCAGCTGCGGTCCTCTGGGATTGTGCACCACTACCAAAATGGATTGGACCTGGATCAGATGGAG 1770
BG10_GF       TGGCACCGAGCCTCCGTGTCACTGCCAACGTGGGACAGGATGTTGTGCTGCGCTGCCACTTGTGCCCTTGCAAGAATGCCTGGAGCTCAGATATCAGATGGATCCAGCTGCGGTCCTCTGGTTTTGTGCACCACTACCGAAATGGAGAGGACCTGGAGCAGATGACA 2156
BG9_GF        TGGCACCGAGCCTCCGTGTCACTGCCAACGTGGGACAGGACGTCGTGCTGTGCTGCCACTTGTCCCCATGCAAGGATGTTCGGAATTCAGACATCAGATGGATCCAGCAGCGGTCCTCTCGGCTTGTGCACCACTACCGAAATGGAGTGGACCTGGGGCAGATGGAG 1859
BG8_GF        TGGCACCGAGCCTCCGTGTCACTGCCAATGTGGGACAGGACGTTGTGCTGCGCTGCCACTTGTCCCCATGCAAGGATGTTCGGAATTCAGACATCAGATGGATCCAGCAGCGGTCCTCTCGGCTTGTGCACCACTACCGAAATGGAGTGGACCTGGGGCAGATGGAG 1530
BG7_GF        TGGCACCGAGCCTCCGTGTCACTGCCATCGTGGGACAGGATGTCGAGCTGCGCTGCCACTTGTCCCCTTGCAAGGATGTTCGGAATTCAGACATCAGATGGATCCAGCTGCGGTCCTCTAGGATTGTGCACCACTACCAAAATGGATTGGACCTGGATCAGATGGAG 1768
BG6_GF        TGGCACCGAGCCTCCGTGTCACTGCCATCGTGGGACAGGATGTCGTGCTGCGCTGCCACTTGTGCCCTTGCAAGGATGCTTGGAGATTGGACATCAGATGGATCCTGCAGCGGTCCTCTGGTTTTGTGCACCACTACCAAAATGGAGAGGACCTGGAGCAAATGCAG 2116
BG5_GF        TAGCACCGAGCCTCCGTGTCACTGCCATCGTGGGACAGGATGTCGTGCTGCGCTGCCACTTGTCCCCTTGCAAGGATGCTTGGAGCTCAGACATCAGATGGATACAGCTGCGGTCCTCTGGGATTGTGCACCACTACCAAAATGGAGAGGACCTGGAGCAAATGCAG 1750
BG4_GF        TAGCACCGAGCCTCCGTGTCACTGCCAACGTGGGACAGGATGTCGTGCTGCGCTGCCACTTGACCCCTTGCAAGGATGCCTGGAGCTCAGACATCAGATGGATACAGCTGCGGTCCTCTGGCATTGTGCACCACTACAAAGATGGAGTGGACCTGGGGCAGATGGAG 2123
BG3_GF        TAGCACCGAGCCTCCGTGTCACTGCCAACGTGGGACAGGATGTCGTGCTGCGCTGCCAGTTGTCCCCTTGCAAGGATGCCTGGAGCTCAGACATCAGATGGATACAGCTGCGGTCCTCTGGGATTGTGCACCACTACGAAGATGGAGTGGACCTGGGGCAGATGGAG 2122
BG2_GF        TGGCACCGAGCCTCCGTGTCACTGCCATGGTGGGACAGGACGTCGTGCTGCGCTGCCAGTTGTCCCCTTGCAAGGATGCTCGGAATTCAGACATCAGATGGATCCAGCAGCGGTCCTCTGGGATTGTGCACCACTACCAAGATGGAGTGGACCTGGAGCAGATGACA 2150
B12BG1Locus   TGGCACCGAGCCTCCGTGTCACTGCCATCGTGGGACAGGATGTTGTGCTGCGCTGCCAGTTGTCCCCTTGCAAGGATGCCTGGAGCTCAGACATCAGATGGATCCAGCACCGGACCTCTGGTTTTGTGCACCACTACCAAAATGGAGAGGACCTGGAGCAAATGGAG 2258
Annotated_BG0 TGGCATTGAACTTCCCTGTCACTGCCACTGTGGGACAGGATGTCGTGCTGCACTGCCACTTGTCCCCTTGCAAGGATGCTCGGAGCTTGGACATCAGATGGATCCAGCACCGGTCCTCTGGTCTTGTGCACCACTACCAAAATGGAGAGGACCTGGAACAGATGGAG 1915

BG13_GF       GAATATAAAGGGAGAACAGAACTGCTCAGGGATGGTCTCTCTGATGGAAATCTGGATTTGCGCATCACTGCTGTGAGCTCCTCTGATAGTGGCTCATACAGCTGTGCTGTGCAAGACGGTGATGCCTATGCAGAAGCTGTGGTGAACCTGGAGGTGTCAGGTCAGTG 1998
BG12_GF       GAATATAAAGGGAGAACAGAACTGCTCAGGGATGGTCTCTCTGATGGAAACCTGGATTTGCGCATCACTGCCGTGACCTCCTCTGATAGTGGCTCATACAGCTGTGCTGTGCAAGATGGTGATGCCTATGCAGAAGCTGTGGTGAACCTGGAGGTGTCAGGTCAGTG 1695
BG11_GF       GAATATGAAGGGAGGACAGAACTGCTCAGGGATGGTCTCTCTGATGGAAACCTGGATTTGCGCATCATTGCTGTGAGCTCCTCTGACAGTGGCTTGTACAGCTGTGTTGTGCAAGATGACGATGGCTATGCAGAAGCTGTGGTGAACCTGGAGGTGTCAGGTCAGTG 1937
BG10_GF       GAATATAAAGGGAGGACAGAACTGCTCAGGAAGGGTCTCTCTGATGGAAACCTGGATTTGCGCATCACTGCTGTGAGCTCCTCCGATAGTGGCTTGTACAGCTGTGCTGTGCAAGATGGTGATGGCTATGCAGAAGCTTTGGTGGAGCTGGAGGTGTCAGGTCAGTG 2323
BG9_GF        GAATATAAAGGGAGAACAGAACTGCTCAGGGATGGTCTCTCTGATGGAAACCTGGATTTGCGCATCACTGCCGTGACCTCCTCTGATAGTGGCTCCTACAGCTGTGCTGTGCAAGATGGTGATGCCTATGCAGAAGCTGTGGTGAACCTGGAGGTGTCAGGTCAGTG 2026
BG8_GF        GAATATAAAGGGAGAACAGAACTGCTCAGGGATGGTCTCTCTGATGGAAACCTGGATTTGCGCATCACTGCCGTGACCTCCTCTGATAGTGGCTCCTACAGCTGTGCTGTGCAAGATGGTGATGCCTATGCAGAAGCTGTGGTGAACCTGGAGGTGTCAGGTCAGTG 1697
BG7_GF        GAATATAAAGGGAGGACAGAACTGCTCAGGGATGGTCTCTCTGATGGAAACCTGGATTTGCGCATCATTGCTGTGAGCTCCTCTGACAGTGGCTTGTACAGCTGTGTTGTGCAAGATGACGATGGCTATGCAGAAGCTGTGGTGAACCTGGAGGTGTCAGGTCAGTG 1935
BG6_GF        GAATATAAAGGGAGGACAGAACTGCTCAGGGATGGTCTCTATGATGGAAACCTGGATTTGCGCATCACTGCTGTGAGCACCTCCGATAGTGGCTCATACAGCTGTGCTGTGCAGGATGGTGATGGCTATGCAGACGCTGTGGTGGACCTGGAGGTGTCAGGTCAGTG 2283
BG5_GF        GAATATAAAGGGAGAACAGAACTGCTCAGGGATGGTCTCTATGATGGAAACCTGGATTTGCGCATCACTGCTGTGAGCTCCTCCGATAGTGGCTCATACAGCTGTGCTGTGCAAGATGGTGATGGCTATGCAGACGCTGTGGTGGACCTGGAGGTGTCAGGTCAGTG 1917
BG4_GF        GAATATAAAGGGAGGACAGAACTGCTCAGGGATGGTCTCTATGATGGAAACCTGGATTTACGCATCACTGCTGTGAGCACCTCCGATAGCGGCTCATACAGCTGTGCTGTGCAAGATGATGATGGCTATGCAGACGCTGTGGTGGACCTGGAGGTGTCAGGTCAGTG 2290
BG3_GF        GAATATAAAGGGAGAACAGAACTGCTCAGGGATGGTCTCTCTGATGGAAACCTGGATTTACGCATCACTTCTGTGAGCTCCTCTGACAGTGGCTCGTACAGCTGTGCTGTTCAAGATGGTGATGGCTATGCAGACGCTGTTGTGGAGCTGGAGGTGTCAGGTCAGTG 2289
BG2_GF        GAATATAAAGGGAGGACAGAATTGCTCAGGAAGGGTCTCTCTGATGGAAACCTGGATTTGCACATCACTGCTGTGAGCTCCTCTGATAGAGGCTCCTACATCTGCATTGTGCAAGATGATGATGGCTATTCAGAAGCTGTGGTGAACCTGGAGGTGTCAGGTCAGTG 2317
B12BG1Locus   GAATATAAAGGGAGAACAGAACTGCTCAGGAAGGGTCTCTCTGATGGAAACCTGGATTTGCGCATCACTGCTGTGAGCACCTCTGATAGTGGCTCCTACAGCTGTGCTGTGCTAGATGGTGATGGCTATGCAGACGCTGTGGTGGACCTGGAGGTGTCAGGTCAGTG 2425
Annotated_BG0 GAATATAAAGGGAGGACAGAACTGCTCAGGGACGGTCTCTCTGATGGAAATCTGTATTTGCACATTACTGCAGTGAGCTCCTCCGATAGCGGCTCATATATGTGCACTGTGCAAGATGATGGCGGCTATGTAGAAGCTATGGTGAACCTGCAGGTATCAGGTCAGTG 2082

BG13_GF       GCTGGGGTGATGTCTCCAGGTGTTGCTGGGTTTGTGTGTCCCACC-CAAACTCTGTCCATTCTCATCCTCTTGTCCATGGATAGACAAGTGAAGCACAGCAGCCTTTGGAAGAGGTTGGGGCTGAACTGTTCCATGAGATGCTGGATTCGCAGCGGGTGCA--GTTT 2162
BG12_GF       GCTAGGG----------------------------------------------------------------TGTCTAAGGATGGAGAGCTGATGGATCGCAGCCTTTGGAAGTGGTCAGGGCTGAACAGCTCCATGAGATGCTGGAATTGCAGTGGGCGCACGCTGT 1798
BG11_GF       GCTGGGGTGACATCTCGAGGTGTCCCTGGGTTTGTGTGTCCCATC-CAACCTCTGTCCATCCTCATCCTCACGTCCATGGATGGAGAGCTGAAGGACAGCAGCCTTTGGAAGAGGCTGGGGCTGAATTGCTCCATGAGATGCTAGAGTTGGAACGGGCACATGGTGT 2103
BG10_GF       TCTGGGGTGACGTCTCCAGGTGTCCCTGGGTTTGTGGGTCCCACC-TGACCTCTGTCCATTCTCATCCTCACATCCATGGATGGAGAGCTGAAGAACAGCAGCCTTTGGAAGAGGTTGGGGCTGAATTGCTCCATGAGATGCTGGAATTAGAGCGGACACATGGTGT 2489
BG9_GF        GCTGGGG----------------------------------------------------------------TGTCTAAGGATGGAGAGCTGATGGATCGCAGCCTTTGGAAGTGGTCAGGGCTGAACAGCTCCATGAGATGCTGGAATTGCAGTGGGCGCACGCTGT 2129
BG8_GF        GCTGGGG----------------------------------------------------------------TGTTCAAGGATGGAGAGCTGACGGATCGCAGCCTTTGGAAGTGGTCAGGGCTGAACAGCTCCATGAGATGCTGGAATTGCAGTGGGCGCACGCTGT 1800
BG7_GF        GCTGGGGTGACATCTCGAGGTGTCCCTGGGTTTGTGTGTCCCATC-CAACCTCTGTCCATCCTCATCCTCACGTCCATGGATGGAGAGCTGAAGGACAGCAGCCTTTGGAAGAGGCTGGGGCTGAATTGCTCCATGAGATGCTAGAGTTGGAACGGGCACATGGTGT 2101
BG6_GF        GCTGGGGTGATGTCTCCAGGTGTCCCTGGGTTTGTGTGTCCCATC-CGACCTCTGTCCATCCTCATCCTCACATCCATGGATGGAGAACTGAAGGACAGCAGCCTTTGGAAGAGCTCAGGGCTGAACAGCTCCATGAGATGCTGGAGTTGGATCGGGCACATGGTGT 2449
BG5_GF        GCTGGGGTGATGTCTCCAGGTGTCACTGGGTTTGTGTGTCCCATC-CGACCTCTGTCCATTCTCATCCTCACGTCCATGGATGGAGAACTGAAGAACAGCAGCCTTTGGAAGAGGTTGGGGCTGAATTGCTCCTTGAGATGCTGGAGTTGCAGCGGGTGCA--GTTT 2081
BG4_GF        GCTGGGGTGACGTCTCCAGGTGTCGCTGGGTTTGTGGGTCCCACC-TGACCTCTGTCCATCCTCATCCTCACGTCCATGGATGGAGAGCTTAAGGACAGCAGCCTTTGGAAGAGCTCAGGGCTGAATTTTCCATGAGATGCTGGAGTTGCAGCGGGCACATGGTGT 2456
BG3_GF        GCTGGGGTGACATCTCCAGGTGTCCCTGGGCTTGTGTGTCCCACC-CAACCTCTGTCCATCCTCATCTTCATGTCCATGGATGGAGAACTGAAGGACAGCAGCCTTTGGAAGAGCTCAGGGCTGAATTTTCCATGAGATGCTGGAGTTGGAACGGGCACATGGTGT 2455
BG2_GF        GCTGGGGTG----------------------------------------------------------------TCTAAGGATGGAGAGCTGAAGGACAGCAGCCTTTGGAAGTGGTCAGGGCTAAAGTGATCCATGAGATCCTGGAGTTGGAGT-GGTGCATGGTGT 2419
B12BG1Locus   GCTGGGGTGACGCCTCCAGGTGTTGCTGGGTTTGTGTGTCCCACCCCGACCTCTGTCCATCCTCATCCTCACATCCATGGATGGAAAGCTGAAGGACAGCAGCCTTTGGAAGAGGTCGGAGCTAAATTGTTCCATGAGATGCTGGTGTTGGAGCAGGTGCACAGTGC 2592
Annotated_BG0 GCTGGGGTGACACCTCCATGTGTTTCTGAGTTTGTGTGTCCCACCCCGACCTCTGTCTATCCTCATCCTCATGTCCATCAGTGGAGAGCTGAAGGACACGAGCCTTTGAAAAAGGTCGGGGCTGAATTGTTCCATGAAATGCCTGACTTCGAGCAGGTGCATTGTGT 2249

BG13_GF       GATTTGTGGATAAGTCTGCATGGATGAGGTGGTTGGGTTGGGTTTCTGGGATGGGTTTCTCCATGTCTCAGTGGCAGAGGGCACACGATGCTGAGCAGTTCCTCCGCCTGTGTCAATATGGGGA-TGTTGCCATTGTGTGTCACTGCTCCCTGCTTGCTGCCCCTTT 2328
BG12_GF       GATTTGGAGATGGGTCTGCATGGATGAGGTGGTTGGGTTGGGTTTCTGGGATGGGTTTCTCCATGGCTCAGTGGCAGTGGGCACACAATGCTGAGCAGCTCCTCCACCTGTGCCAATGTGGAGA-TGCTGCTATTGTGTGTCACTGCTCCCTGCTTGCTGCCCCTTC 1964
BG11_GF       GATTTGAAAATGGATATGCATGGATGAGGTGGTTGGGTTGGGTTTCTGGGATGGGTTTCTCCATGGCTCAGTGGCAATGGGCACACAATGCTGAGCAGCTCCTCCACCAGTGTCAATATGGGGAGCGCTGCCATTGTGTGTCACTGCTCCCTGGTTGTTGCCCCTTC 2270
BG10_GF       GATTTGGGGATAAGACTGCATGGATGAGGTGGTTGGGTTGGGTTTCTGGGATGGGTTTCTCCACATCTCAGTGGCAGTGGGCACATGATGCTGAGCAGCTCCTCCGTCTGTGCCAAGCTGGGGA-CACTGCCATTGTATGTCATTGTTCCAAGGTTGCTGCCCCTTT 2655
BG9_GF        GATTTGGAGATGGGTCTGCATGGATGAGGTGGTTGGGTTGGGTTTCTGGGATGGGTTTCTCCATGGCTCAGTGGCAGTGGGCACACAATGCTGAGCAGCTCCTCCGCCTGTGCCAATGTGGAGA-TGCTGCTATTGTGTGTCACTGCTCCCTGCTTGCTGCCCCTTC 2295
BG8_GF        GATTTGGAGATGGGTCTGCATGGATGAGGTGGTTGGGTTGGGTTTCTGGGATGGGTTTCTCCATGGCTCAGTGGCAGTCGGCACACAATGCTGAGCAGCTCCTCTGCCTGTGCCAATGTGGGGA-TGCTGCTATTGTGTGTCACTGCTCGCTGGTTGCTGCCCCTTC 1966
BG7_GF        GATTTGAAAATGGATCTGCATGGATGAGGTGGTTGGGTTGGGTTTCTGGGATGGGTTTCTCCATGGCTCAGTGGCAGTGGGCACACAATGCTGAGCAGCTCCTCCACCTGTGTCAATATGGGGAGCGCTGCCATTGTGTGTCACTGCTCCCTGGTTGTTGCCCCTTC 2268
BG6_GF        AATTTGAAAATGGATATGCATGGATGAGGTGGTTGGGTTGGGTTTCTGGGATGGGTTTCTCCACATCTCAGTGGCAGAGGGCACACGATGCTGAGCAGCTCCTCCGCCTGTGCCAATATGGGGA-CGCTGCCATTGTGTGTCACTGCTCCCTGGTTGTTGTCCCTTC 2615
BG5_GF        GATTTGTGGATAAGTCTGCATGGATGAAGTGGTTGGGTTGGGTTTCTGGGATGCCTTTCTCCATGGCTCAGTGGCAGTGGGCACATGATGCTGAGCAGCTCCTCTGCCTGTGCCAATATGGGGA-TGTTGCCATTGTGTGTCACTGCTCCCTGGTTGCTGCCCCTTT 2247
BG4_GF        GATTTGAAAATGGATATGCATGGATGAGGTGGTTGGGTTGGGTTTCTGGGATGGGTTTCTCCACGTCTCAGT---------------------TTCTCCACGTCTTTCTGTGCCAATATGGGGA-CGCTGCCATTGTGTGTCACTGCTCCCTGGTTGTTGCCCATTC 2601
BG3_GF        GATTTGAAAATGGATCTGCATGGATGAGGTGGTTGGGTTGGGTTTCTGGGATGGGTTTCTCCACGTCTCAGTGGTAGTGGGCATACGATGCTGAGCAGCTCTTCCATCCATGCCAATATGGGGA-CGCTGCCATTGTGTGTCACTGCTCGCTGGTTGCTGCCTCTTC 2621
BG2_GF        GATTTGGGGATGGGTCTGTGTGGATGAGGTGGTTGGGTTGGGTTTCTGGGATGGGTTTTTCTGTGTCTTAGTAGCAGTGGGCACACGATGCTGAGCAGCTCCTCTGCCTGTGCCAAGCTGGGTA-TGCCGCCATTGTGTGTCACTGCTCCCTGGTTGTTGCCCCTTT 2585
B12BG1Locus   AATTTGTGGATAAGTCTGCATGGATGAGGTGGTTGGGTTGGATTTCTGGGATGGGTTTCTCCATGGCTCAGTGGCAGTGGGCACACGATGCTGAGCAGCTCCTCCACCTGTGCCAATATGGGGA-TGCCGTCATTGTGCATCACTGCTCCCTGGTTGCTGCCCCTTT 2758
Annotated_BG0 GATTTGGGGATGGATATGCATGGATGAGGTGATTTCATTGGGTTTCTTTGATGGGTTTCTTCATGTCTCAGTGGCAGAGGGCACAGTCTGCTGAGTAGCTCCTCCACCTGTGCCAATCTGAGAA-CACTGCCATTGTGCATCAGTGCTCCCTGGTTGCTGCCCCTTT 2415

BG13_GF       GGGTTCTGTGATCTCGAGAACTCAAAGTCGTGCTTCTCCACATAAGGCAGTGGAAAAGGAACCCTTGTCCTGATGTTTTTTCCAGATCCCTTTTCCCAAATCGTCCATCCCTGGAAGGTGGCTCTGGCTGTGATAGTCACACTTCTGGTTGGGTCATTTGTCATCAT 2495
BG12_GF       GGGTTCTGTGATCTCCAGAAGTCAAAGTCATGCTTGTCTACATAAGGCAATGGAAAAGGAACCCTTGTCCTGATGTTTTTTTCAGATCCCTTTTCCCAGATCATCCTTTACTGGACAGTGGCTCTGGCTGTGATCATCACACTTCTGGTTGGGTCATTTGTCGTCAA 2131
BG11_GF       GGGTTCTGTGATCTCCAGAAGTTGAAGTCATGTTTGTCCACATAAGGCAATGGAAAAGGAACCATTTTCCTGATGTTTTTTCCAGATCTCTTTTCCCAGATCGTCCATCCCTGGAAGGTGGCTCTGGCTGTGGTCGTCACAATTCTGGTTGGGTCATTTGTCATCAT 2437
BG10_GF       GGGTTCTGTGATCTTGAGAAGTCGAAGTCATGCTTGTCTACATAAAGCAATGGAAAAGGAACTCTTGTCCTGATGTTTTTTCCAGACCCCTTTTCCCAGATCGTCCATCCCTGGAAGGTGGCTCTGGCTGTGATCGTCACAATTCTGGTTGGGTCATTTGTCATCAT 2822
BG9_GF        GGATTCTGTGATCTCCAGAAGTCAAAGTCATGCTTGTCTACATAAGGCAATGGAAAAGGAACCCTTGTCCTGATGTTTTTTCCAGATCCCTTTTCTATGATCATCCTTTACTGGACAGTGGCTCTGGCTGTGATCATCACACTTCTGGTTGGGTCATTTGTCGTCAA 2462
BG8_GF        GGGTTCTGTGATCTCCCAAGGCT-GAGTCTTGCTTTTCCACATATGGGAATTTAAAAGG-ACCCTCTTCTTGACATTTCTTCCAGACCCCTTTTCTATGATCATCCTTTACTGGACAGTGGCTCTGGCTGTGATCATCACACTTCTGGTTGGGTCATTTGTCGTCAA 2131
BG7_GF        GGGTTCTGTGATCTCCAGAAGTTGAAGTCATGCTTGTCCACATAAGGCAATGGAAAAGGAACCCTTGTCCTGATGTCTTTTCCAGATCCCTTTTCCCAGATCGTCCATCCCTGGAAGGTGGCTCTGGCTGTGGTCGTCACAATTCTGGTTGGGTCATCTGTCATCAT 2435
BG6_GF        GGGTTCTGTGATCTCCAGAAGTCGAAGTCGTGTTTGTCCACATAAGGCAGTGGAAAAGGAACCCTTGTCCTGATGTCTTTTCCAGATCCCTTTTCCCAGATCGTCCATCCCTGGAAGGTGGCTCTGGCTGTGGTCGTCACAATTCTCGTTGGGTCATTTGTCATCAT 2782
BG5_GF        GGGTTCTGTGATCTCGAGAAGTCAAAGTCATGCTTCTCCACATAAGGCAGTGGAAAAGGAACTATTTTCCTGATGTTTTTTCCAGATCCCTTTTCCCAGATCCTTCATCCCTGGAAGGTGGCTCTGGCTGTGATCGTCACACTTCTGGTTGGGTCATTTGTCATCAT 2414
BG4_GF        GGGTTCTGTGATCTCGAGAAGTTGAAGTCGTGCTTCTCCACATAAGGCAATGGAAAAGGAACCCTTGTCCTGATGTTTTTTCCAGATCCCTTTTCCCACATCATCCATCCCTGGAAGGTGGCTCTGGCTGTGATTGTCACACTTCTGGTTGGGTCATTTGTCATCAA 2768
BG3_GF        GGGTTCTGTGATCTCGAGAAGTTGAAGTCGTGCTCCTCCACATAAGGCAATGGAAAAGGAACCCTTGTTCTGATGTTTTTTCCAGATCTCTTTTCCCAGATCGTCCATCCCTGGAAGGTGGCTCTGGCTGTGGTTGTCACACTTCTGGTTGGGTCATTTGTCATCAA 2788
BG2_GF        GGGCTCTGTGATCCCC-CAAGTCCAAGTTATGCTTTTCCACATATCACAATGGAAAAGGAACCCTTTTCTTGATGTTTTTTCCAGATCCCTTTTCCCAGATCATCCATCCTTGGAAGGTGGCTCTGGCTGTGGTCATAATTCCTCTGGCTGGGTCATTTGTCATCAA 2751
B12BG1Locus   GGGTTCTGTGATGTCC-CAAGGCTGAGTCTTGCTTTTCCACTTATGGGAATGGAAAAGGAACCCTTGTCCTGATGTTTTTTCCAGACCCCTTTTCCCAGATCACCCATCCCTGGAAGGTGGCTCTGGCTGTGATCGTCACAATTCTGGTTGGGTCATTTGTCATCAC 2924
Annotated_BG0 GGATTCTGTGATCTTC-CAAGTCTGAGTCTTGCTTTTCCACATATGGGAATGTAAAAGTAATCCTT-----------TTTTCCAGATCCCTTTTCCCATATTGTCCATCCCTGGAAGGTGGCTCTGGCTGTGGTCCTCACACTTTTGTTTGCGTCATTCGTCATCAT 2570

BG13_GF       TGCTTTTCTCTATAGAAAGAAAGGTGAGCTGACAGTGGAGGGGATGGAGCACAGGGAGGTGTTGTGCATGGACAGGGATGGTCGGGGTGGTGCTGAGCTGTGGTCCATGGAGGTACACAGGAGGAGGAAGGGAGA-TTTTTCCTGAGATTCCCAGTGCTCATTAAAT 2661
BG12_GF       TGTTTTTCTCCATAGAAAGAAAGGTAAGCTGACAGTGGAGGGGATGGAGCACAGGGAGGTGTTGTGCATGGACAGGGATGGTCGGGGTGGTGCTGAGCTCTGGTCCATGGAGGTACACAGGAGGAGGAACGGTGA-CTTTTCATGGGATTCCCAGTGCTCATTAAAT 2297
BG11_GF       TGCTTTTCTCTATAGGAAGAAAGGTGAGCTGAGAGCGGAGGGGATGGAGCACAGGGAGGTGTTGTGCATGGACAGGGATGGTCGGGGTGGTGCTGAGCTCTGGTCCACGGAGGTACACAGGAGGAGGAAGGGAGA-TTTTTCCTGACATTCCCAGTGCTCATTAAAT 2603
BG10_GF       TGCTTTTCTCTATAGGAAGAAAGGTGAGCTGAGAGCGGAGGAGATGGTGCACAGGGAGGTGTTGTGCATGGACAGGGATGGTCGGGGTGGTGCTGAGCTCTGGTCCATGGAGGTACACAGGAGGAGGAAGTGTGA-CTTTCCATGGGATTCCCAGTGCTCATTAAAT 2988
BG9_GF        TGTTTTTCTCCATAGAAAGAAAGGTGAGCTGACAGTGGAGGGGATGGAGCACAGGGAGGTGTTGTGCATGGACAGGGATGGTCGGGGTGGTGCTGAGCTCTGGTCCACGGAGGTACACAGGTGGAGGAACGGTGA-CTTTTCATGGGATTCCCAGTGCTCATTAAAT 2628
BG8_GF        TGTTTTTCTCCATAGAAAGAAAGGTGAGCTGAGAGCGGAGGGGATGGAGCACAGGGAGGTGTTGTGCATGGACAGGGATGGTCGGGGTGGTGCTGAGCTGTGGTCCACGGAGGTACACAGGTGGAGGAACCGTGA-CTTTTCATGGGATTCCCAGTGCTCATTAAAT 2297
BG7_GF        TGTTTTTCTCTGTAGGAAGAAAGGTGAGCTGAGAGCGGAGGGGATGGAGCACAGGGAGGTGTTGTGCATGGACAGGGATGGTCGGGGTGGTGCTGAGCTCTGGTCCACGGAGGTACACAGGAGGAGGAAGGGAGA-TTTTTCCTGACATTCCCAGTGCTCATTAAAT 2601
BG6_GF        TGTTTTTCTCTGTAGGAAGAAAGGTGAGCTGAGAGCGGAGGGGATGGAGCACAGGGAGGTGTTGTTCATGGACAGGGATGGTCGGGGTGGTGCTGAGCTCTGGTGTACAGAGGTACACAGGAGGAGGAAGGGAGA-TTTTTCCTGACATTCCCACTGCCCATTAAAT 2948
BG5_GF        TGCTTTTCTCTATAGGAAGAAAGGTGAGCTGACAGTGGAGGGGATGGAGCACAGGGAGGTGTTGTGCATGGACAGGGATGGTCGGGGTGGTGCTGAGCTCTGGTCCATGGAGGTACACAGGAGGAGGAAGGGAGA-TTTTTCCTGACATTCCCACTGCCCATTAAAT 2580
BG4_GF        TGTTTTTCTCTATAGGAAGAAAGGTGAGCTGACAGCAGAGGGGATGGAGCACAGGGAGGTGCTGTTCATGGACAGGGATGGTCGGGGTGGTGCTGAGCTGTGGTCCATGGAGGTACACAGGAGGAGGAAGGGAGA-TTTTTCCTGACATTCCCACTGCTCATTAAAT 2934
BG3_GF        TGTTTTTCTCTATAGGAAGAAAGGTGAGCTGACAGC-GGGGGGATGGAGTACAGGGAGGTGTTGTTCATGGACAGGGATGGTCGGGGTGGTGCTGAGGTCTGATTCATGGAGGTACACAGGAGGAGGAAGGGAGA-TTTTTCCTGACATTCCCACTGCTCATTAAAT 2953
BG2_GF        TGTTTTTCTTTATAGAAAGAGAGGTGAGCTGAGAGCAGAGGGGATGGAGCACATGGAGGTGTTGTGCATGGACAGGGATGGTTGGGGTGGTGCTGAGCTCTGGTCCATGGAGGTACACAGGAGGAGGAAGGGAGA-TTTTTCCTGACATTCCCAATGCTCATTAAAT 2917
B12BG1Locus   TGTTTTTCTCTATAGGAAGAAAGGTGAGTTGAGCATGGAGGGGATGGATCACAGGGAGGTGTTGAGCAGGGATAGGGATGGTCGGGGTGGTGCTGAGCTCAGGTCCGTGGAGGTATGCA---GGAGAAAGGGAGA-TTTTTCTGGGGATTGACAGTGGTCATTAAAT 3087
Annotated_BG0 TGTTTTTCTCCATAGAATGCAAGGTGAGCTGAGAGTGGAGGGGATAGTGCACAGGGAGCTATTCTGCAGTTACGGGGATGGTTGGGGTGGTGCTGAGCTCTTGTCCATGGAGGTACACGGGAGGAGGAAGGGAGATTTTTTCCTCAGATTCACAGTGGTCATTAAAT 2737

BG13_GF       AACTTTTGCCTTTCTTTTG------GGGAATA----AAGGGGAAGAAGAAAAGGGGTGTGGGTGGGCAGATAGGAATGTGGCTGGACCGTGGGGCAGACGGAAAGGTCCAGACCCTCTGGAGAAGTCCCCACAAACCAAGCTGCCCTGCTGACCACCTCTTCCTCTA 2818
BG12_GF       AACATTTGCCTTTCTTTTT------GGGAATAAAGGAAGGGGAAAAAGGATAGTGGTAAGGGTGGGCAGATAGGAATGTGGCTGGACTGTGGGGCAGGTGGAAA-GTCCAAACCCTCTGGAGAAGTCCCCACAAACCAAGCTGCCCTGCTGACCAGCTATTTCTCTG 2457
BG11_GF       AACATTTGCCTTTCTTTTT------GGGAATG----AA-GGAAGGAAAAAAAAGGGGTGTGGGTGGGAAGACAGGAATGTGGCTGGACCGTAGGGCAGGTGGAAAGGTTCAGACCCTCTGGAGATGTCCCCACAAACCAAGTTGCCCTGCTGACCAGCTATTTCTCTG 2759
BG10_GF       AACATTTGACTTTCTTTTT------GGGAATGAAGGAAGGGGAAAAAGGAAAGGGGTGTGGGTGGGAAGATAGGAATGTGGGTGGACTGTGGGGCAGGTGGAAAGGTCCTGACCCTCTTGAGGCATCCCCA-AAACCAAGCTGCCCTGCTGACCACCTCTTCCTCCA 3148
BG9_GF        AACATTTGCCTTTCTTTTT------GGGAATAAAGGAAGGGGAAAAAGGATAGTGGTAAGGGTGGGCAGATAGGAATGTGGCTGGACTGTGGGGCAGGTGGAAA-GTCCAAACCCTCTGGAGAAGTCCCCACAAACCAAGCTGCCCTGCTGACCAGCTATTTCTCTG 2788
BG8_GF        AACATTTGCCTTTCTTTTG------GGGAATAAAGAAGGGGAAAAACGATAGTGGTAAGGGTGGGCAGATAGGAATGTGGCTGGACTGTGGGGCAGGTGGAAA-GTCCAAACCCTCTGGAGAAGTCCCCACAAACCAAGCTGCCCTGCTGACCAGCTATTTCTCTG 2457
BG7_GF        AACATTTGCCTTTCTTTTT------GGGAATG----AA-GGAAGGAAAAAAAAGGGGTGTGGGTGGGAAGACAGGAATGTGGCTGGACCGTAGGGCAGGTGGAAAGGTTCAGACCCTCTGGAGATGTCCCCACAAACCAAGTTGCCCTGCTGACCAGCTATTTCTCTG 2757
BG6_GF        AACATTTGCCTTTCTTTTG------GGGAATGAAGGAAGGAAAAAAAGA-----AGTGTGGGTGGGCAGATAGGAAAGTGGGTGGACCGTGGGGCAGGTGGAAAGGTCCAGACCCTCGGGGACGTCCCCA-AAACCAAGCTGCCCTGCTGACTACCTCTTCCTCCA 3103
BG5_GF        AACATTTGCCTTTCTTTTG------GGGAATA----AAGGGGAAGAAGAAAAGGGGTGTGGGTGGGCAGACAGGAATGTGGCTGGACCGTGCGGCAGACGGAAAGGTCCAGACCCTCTGGAGTTGTCCCCA-GAACCAAGCTGCCCTGCTGACTACCTCTTCCTCCA 2736
BG4_GF        AACATTTGCCTTTCTTTTG------GGGAATGAAGGAAGGAAAAAAAGG-----GGTGTGGGTGGGAAGATAGGAAAGTGGGTGGACCGTGCAGCAGACGGAAAGGTTCAGACCCTC-GGGGTCGTCCCCA-AAACCAAGCTGCCCTGCTGACCACCTCTTCCTCTG 3088
BG3_GF        AACATTTCCCTTTCTTTTG------GGGAACAAAGGAAGGGGAAAAAGGAAAGTGGTGTGGGTGGGCAGATAGGAATGTGGCTGGACCGTGCAGCAGACGGAAAGGTCCAGACCCTCTGGAGACATCACCACAAACCAAGCTGTCCTGCTGACCACCTCTTCCTCTG 3114
BG2_GF        AACATTTCCCTTTCTTTTT------GGGAATGATGGAAGGGGAAAAAGGAAAGTGGTGTGGGTGGGAAGATAGGGATGTAGCTGGACCGTGGGGCAGGTGGAAA-GTCCAGACCCTCTGGAAAAGTCCCCACAAACCAAGCTGCCCTGCTGACCACCTCTTCCTCTG 3077
B12BG1Locus   AACATTTGCCGTTTGATT-------GGGAATGAAGGAAAGGGAAAAAGG-----GGTGTGGGCGGAAAGATAGGAATGTGGGTGGACCGTGGGGCAGGTGGAAAGGTCCAGACCCTCTGGAGACGTCCCCAGAAACCAAGCTGCCCTGCTGACCAGCTATTTCTCTG 3242
Annotated_BG0 AACATTTGCCTTTTCTTTTTTAATGGGGAATGAAGGAAGGGGAAAAAGAAAAAGAGTATAGGTGAGAAGACAGGAATGTGGCTGGATCATGGGGCATGTGGAAAGGTCTAGAACCTCTTGAGACGTCACCAGAAACCAGGTAGCCCTGCTGACCACCTCTTTCTCCA 2904

BG13_GF       CTTTGTTTTCCAGCGGCACAGAGCAGAGAGCTGAGTGAGTCCTTCCAGCTCCTTCCACCACCAAAGTCCCTTTAATGGAACTGATAGAAGACTGCAGAGTGCTGGGTTTATGCC---------------------------------------------------TT 2934
BG12_GF       CTTTGTTTTCCAGTGGCACAGAGCAGAGAGCTGAGTGAGTCCTTCCAGCTCCTTCCACCACCAAAGTCCCTTTAATGGAACTGACAGCAGACTGCAGAGTGCTGGG-TTATGCC---------------------------------------------------GT 2572
BG11_GF       CTTTGTTTTCCAGTGGCACAGAGCAGAGAGCTGAGTGAGTCCTTCCAGTCCCTTCCACCACCAAAGCCTCTTTAATGGAAATGATAGAAGACTGCAGAGTGTTGGGTTTATGCC---------------------------------------------------AT 2875
BG10_GF       CATTGTTTTCCAGCGACACAGAGCAGAGAGTTGAGTGAGTCCTTCCATCCCCTTCCACCACCAAAGTCCCTTTAATGGAACTGATAGAAGACTGCAGAGTGCTGGGTTTATGCC---------------------------------------------------AT 3264
BG9_GF        CTTTGTTTTCCAGTGGCACAGAGCAGAGAGCTGAGTGAGTCCTTCCATCCCCATCCACCACCAAAGTCCCTTTAATGGAACTGACAGCAGACTGCAGAGTGCTGGG-TTATGCC---------------------------------------------------AT 2903
BG8_GF        CTTTGTTTTCCAGTGGCACAGAGCAGAGAGCTGAGTGAGTCCTTCCATCCCCATCCACCACCAAAGTCCCTTTAATGGAACTGACAGCAGACTGCAGAGTGCTGGG-TTATGCC---------------------------------------------------AT 2572
BG7_GF        CTTTGTTTTCCAGTGGTACAGAGCAGAGAGCTGAGTGAGTCCTTCCAGTCCCTTCCACCACCAAAGCCTCTTTAATGGAAATGATAGAAGACTGCAGAGTGTTGGGTTTATGCC---------------------------------------------------AT 2873
BG6_GF        ATTTGTTTTCCAGCGGCACAGAGCAGAGAGCTGAGTGAGTCCTTCCAGCCCCTTCCACCACCAAAGTCCCTTTAATGGAACTGATAGAAGACTGCAGAGTGCTGGGTTTATGCC---------------------------------------------------TT 3219
BG5_GF        ATTTGTTTTCCAGCGGCACAGAGCAGAGAGCTGAGTGAGTCCTTCCAGCCCCTTCCACCACCAAAGTCCTTTTAATGGAACTGATAGAAGACTGCAGAGTGCTGGGTTTATGCCATGTGCTGGGGCTGTGGGATGTTTGGGGCCTTGGGATGTTGGGATCTTGGGAT 2903
BG4_GF        CTTTGTTTTCCAGCCTCACAGAGCAGAGAGCTGAGTGAGTCCTTCCAGTCCCTTCCACCACCAAAGTCCCTTTAATGGAACTGATAGAAGACTGCAGAGTTCTGGGTTTATGCC---------------------------------------------------AT 3204
BG3_GF        CTTTGTTTTCCAGCGGCACAGAGCAGAGCATTGAGTGAGTCCTTCCAGTCCCTTCCACCACCAAAGTCCCTTTAATGGAACTGATAGAAGACTGCAGAGTGCTGGGTTTATGCC---------------------------------------------------AT 3230
BG2_GF        CTTTGTTTTCCAGCCGCACAGAGCAGAGAGCTGAGTGAGTCCTTCCCGCCCCTTCCACCACCAAATTCCCTTCAATGGA-----------ACTGCAGAGTGCTGGGTTTATGCC---------------------------------------------------AT 3182
B12BG1Locus   CTTTGTTTTCCAGCAGCTGAGACCACAAAGCAGAGTGAGTCCTTCCAGCCCCTTCTACCACCAAAATTCCTCTATTGGAACTGATAGAAGACTGCAGAGTGCTGGGCTTTTGTG---------------------------------------------------AT 3358
Annotated_BG0 CTTTGTTTTTCAGCGGCACAGACCAAAATCTGAGTGAGTCCTTCCAGCCCCTTCCACAACCAAAGTTCCTTTAATGGAACTGACAGAAGACTGGAGAGTCCTAGGTTTATGTG---------------------------------------------------AT 3020
```

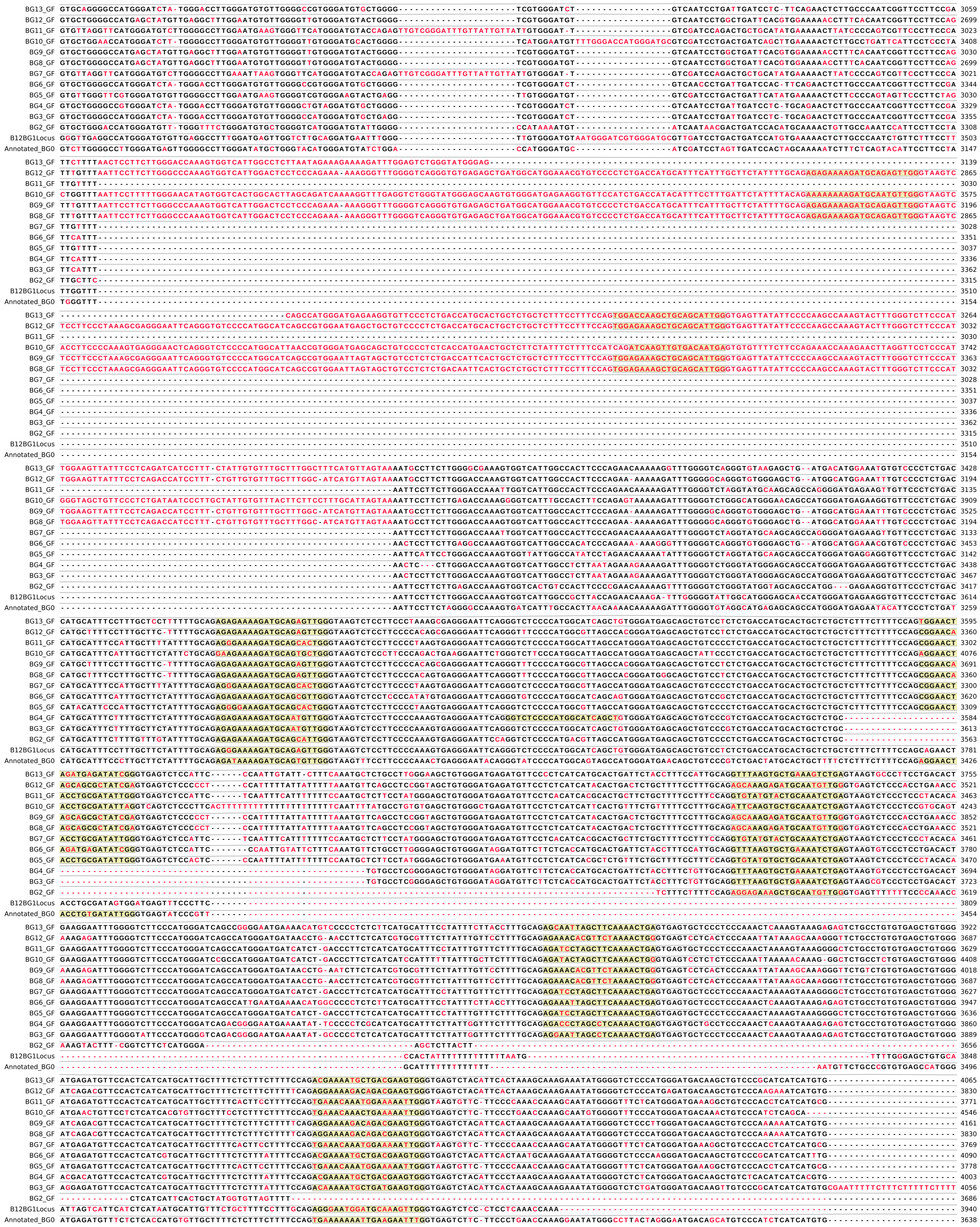

```
BG13_GF      -------------------GTGATTCTTC-------------------TGTATTTTTTTTTTTGCAGAGCATTGCAATTTAGATCTGAGTAAGTTGCAGTCACTGAACTGAGGGAATGTGGGGTCTTCCCAAGGGACTGTGTATGTATGGGATGAAAAATCCCCTCT 4194
BG12_GF      -------------------GTGCTTTTTCTTGTCTT----TTTATTTATTTATTTATTTATTTGCAGAGAATTGGAATTCAGTGCTGAGTAAGTTGCAGTCACTGAACTGAGGGAATGTGGGGTCTTCCTAAGGGACTGCGTA-------GGGGAGAAGTTC----- 3962
BG11_GF      -------------------TTGGTTTTT-----------------------CTTTCTGTTTTGCAGAGATTCAGAATTCAGTCTTGGGTAATTTGCAGACACCAACTGCGGGAATTTGTGGTCCCCCAGAGGACAATGTA----TGGGATGAAAATCCCCTCT 3891
BG10_GF      -------------------TCCATTGCT-------------------TTCTTTTTCTTTTTTACAGACATACGGAATTCACTAATGAGTAAGCTGCACTCAGTAAATGGAGCAAATTTGGGGTCCTTCCATGGGACTGCGTA----TGGGATGAAAGATCCCCTCT 4670
BG9_GF       -------------------GTGCTTTTTCTTGTCTTTTTATTTACTTATTTATTTATTTATTTGCAGAGAATTGGAATTCAGTGCTGAGTAAGTTGCAGTCACTGAACTGAGGGAATGTGGGGTCTTCCTAAGGGACTGCGTA-------GGGGAGAAGTTC----- 4297
BG8_GF       -------------------GTGCTTTTTCTTGTCTTTTTATTTATTTATTTATTTATTTATTTGCAGAGAATTGGAATTCAGTGCTGAGTAAGTTGCAGTCACTGAACTGAGGGAATGTGGGGTCTTCCTAAGGGACTGCGTA-------GGGGAGAAGTTC----- 3966
BG7_GF       -------------------TTGGTTTTT-----------------------CTTTCTGTTTTGCAGAGATTCAGAATTCAGTCTTGGGTAATTTGCAGTCACCAACTGAGGGAATTTGTGGTCCCCCAGAGGACAATCTA----TGGGACAAAAATCCCCTCT 3889
BG6_GF       -------------------GTGCTTCTT-------------------CTGTCTTTTTTTTTTGCAGAGGATTGCAATTCAGAGCTGAGTAAGTTGCAGTCACTGAACTGAGGGAATGTGGGGTCTTCCCAAGGGACTGTGCA----TGGGATGAAAAATCCCCTCT 4214
BG5_GF       -------------------TTGGTTTTT-----------------------CTTTCTGTTTTGCAGAGATTCAGAATTCAGTCTTGGGTAATTTGCAGTCACCAACTGAGGGAATTTGTGGTCCCCCAGAGGACAATCTA----TGGGACAAAAATCCCCTCT 3898
BG4_GF       -------------------GTGCTTCTT-------------------CTGT-CTTTTTTTTTGCAGAGGATTGCAATTCAGAGCTGAGTAAGTTGCAGTCACAGAACTGAGGGAATGTGGGGTCTCCCAAGGGACTGTGTA----TGGGATGAAAAATCCCCTCT 4126
BG3_GF       TCTTTTTTTCTTTTTCTTTTTCCTTTTT-------------------TTTT-TTTTCTTTTTGCAGAGGATTGCAATTCAGAGCTGAGTAAGTTGCAGTCACAGAACTGAGGGAATGTGGGGTCTCCCAAGGGACTGTGTA----TGGGATGAAAAATCCCCTCT 4198
BG2_GF       -------------------TTCCTCCCT-------------------ATTTTTTTAATTCCTG--------TTTGGTACCGAACTTGCCATTACCCACTTACTAGAAAAAAGAAT-TGGATTGTAGGCATGGGAGCAGCCA----TGCGATGCGGTGTTCCCTCT 3801
B12BG1Locus  ----------------------------------------------------------------------------------------------------------------------------------------------------------------------------- 3940
Annotated_BG0 -------------------TTGATTTTTCTTTTTTT-----------TTTTTCTCTTTCTTTGCAGAGAAAGAGAATTCACAATTGAGTAAGTTGCATTCACTAAACTGAG------------------------------------------------------------ 3719

BG13_GF      GACCATGCACTGCTTTT-CTCTTTCTTTTCCAGAGAAAGACTGTGAAGAGATGCGTGAGTCTCCCCTCCCAAAATAAAAACATTGGGGTCCTCATGT---------GGATGAGATGTTCCTCTCATCACGCACTGTTTCTACTTTTCTTTGCAGGTTCTGGCGTT 4350
BG12_GF      --CCATGCACTGCTTTT-CTCTTTCTTTTCCAGAGAAAGACAGTGAAGAGATGGGTGAGTCTCTCCTCCCAAATTAAAAACGTTGGGGTTCCCATGTGGGAGCTGTGGGATGAGATGTTCCTCTCATCACCATCTTTTTTACTTTTCCTTTGCAGGTTATGGCTTT 4126
BG11_GF      GACCATACACTGCTTTT-CTCTTTCTTTTCCAGAGAAACGGTATGAGAATACGGGTGAGTCTCCTCTCCCAAATTAAAACCATTGCGGTCTTCCTTGTGAGCTGTAGGATGAGATGTTTCTCTCATCATGCACTGTTTCTGCTTTTCCTTTGAAGATTTAAGTGCT 4057
BG10_GF      GACCATGCACTGCTCTT-CTCTTTCTTTTCCAGAGAAACAGTATGAAATGACAGGTGAGTCTCCCCTCACAGATTAAAAACATCGGGGTCTTCCTGTGTGAGTTGTGGGATGAGATGTTCCTCTCATCAGGCCTTGTTTTTGCTTTTCCTTTGCAAGTT-AAATGCT 4835
BG9_GF       --CCATGCACTGCTTTT-CTCTTTCTTTTTCAGAGAAAGACAGTGAAGAGATGGGTGAGTCTCTCCTCCCAAATTAAAAACGTTGGGGTTCCCATGTGGGAGCTGTGGGATGAGATGTTCCTCTCATCACCATCTTTTTTACTTTTCCTTTGCAGGTTATGGCTTT 4461
BG8_GF       --CCATGCACTGCTTTT-CTCTTTCTTTTCCAGAGAAAGACAGTGAAGAGATGGGTGAGTCTCTCCTCCCAAATTAAAAACGTTGGGGTTCCCATGTGGGAGCTGTGGGATGAGATGTTCCTCTCATCACCATCTTTTTTACTTTTCCTTTGCAGGTTATGGCTTT 4130
BG7_GF       GACCATACACTGCTTTT-CTCTTTCTTTTCCAGAGAAACGGTATGAGAATACGGGTGAGTCTCCTCTCCCAAATTAAAACCATTGCGGTCTTCCTTGTGAGCTGTAGGATGAGATGTTTCTCTCATCATGCACTGTTTCTGCTTTTCCTTTGAAGATTTAAGTGCT 4055
BG6_GF       GACCATGCACTGCTTTT-CTCTTTCTTTCCCAGAGAAAGACTGTGAAGAGATGGGTGAGTCTCCCCCTCCCAAATTAAAAACGTTGGGGTCCTCATGTGGGAGCTGT-GGATGAGATGTTTCCTCTCATCACGCACTGTTTCTACATTTCCTTTGCAGGTTCTGGCGTT 4379
BG5_GF       GACCATACACTGCTTTT-CTCTTTCTTTTCCAGAGAAATGGTATGAGAATACGGGTGAGTCTCCTCTCCCAAATTAAAACCATTGCGGTCTTCCTTGTGAGCTGTAGGATGAGATGTTTCTCTCATCATGCACTGTTTCTGCTTTTCCTTTGAAGATTTAAGTGCT 4064
BG4_GF       GACCATGCACTGCTTTT-CTCTTTCTTTTGCAGAGAAACACTGTGAAGAGATGGGTGAGTCTCCCCTCCCAAATTAAAAACGTTGGGGTCTTCATGTGGGAGCTGT-GGATGAGATGTTTCCTCTCATCACGCACATTTCTCCTTTTCTTTGCAGGTTTTGGCGTT 4291
BG3_GF       GACCATGCACTGCTTTTCCTCTTTCTTTTCCCAGAGAAAGACTGTGAAGAGATGGGTGAGTCTCCTCTCCCAAATTAAAAACATTGGGGTCTTCATGTGGGAGCTGT-GGATGAGATGTTCCTCTCATCACGCTCTGTTTCTACTTTTCCTTCGCAGGTTCTGGCGTT 4364
BG2_GF       GACCATGCATTTCCTTTGCTTCTATTTTGCAGGAAGAAAA-----------------------------------------------------------------------------------------------------------------------------GAT 3844
B12BG1Locus  ----------------------------------------------------------------------------------------------------------------------------------------------------------------------------- 3940
Annotated_BG0 ----------------------------------------------------------------------------------------------------------------------------------------------------------------------------- 3719

BG13_GF      GCAGATCTGAGTAAGTCTCCCTCCCAACAGTGAAGGAATTTGGGGTCTTCCCATGGGATCAGCCATGGGACGATCATCTGAGCC-CTCTCATCATGCATTTCATATTTGTTTCTTTTGCAGAGAAACTGGCTAGAGAACTGGGTGAGTGCTGCCTCCCAAATTAAAT 4516
BG12_GF      GCAGAACTGAGTAAGTCTCCCTCCCAACACGGAAGGGATTTGTGGTCTTCCCATGGGATCAGCCATGGGATGATCATCTGACCC-CTCTCATCATGCATTTCATATTTGTTTCTTTTGCAGAGAAACTGGCTGCAGAACTGGGTGAGTGCTGCCTCCCAAATTAAAT 4292
BG11_GF      GCAAATCTCAGTATTGCTCCCTATGTACACGGAAGGAATGTGGGGTCTTCCCAT------------GGGATGATAATCTCACTC-TCCTCACAATGAGTTTCTTATTTGTTTCTTTTGCAGAGGAACTGGCTGCAGATCTGGGTGAGTGCTGCCTCCCAAATTAAGA 4211
BG10_GF      GCAAATCTCAGTAACTCTCATCTGTACACAGAAGGAATTTTGGGTCTCCCCATAGGATCAGCTGTGAGATGATAATCTGACGC-TTCTCACCAAGCATTTCGTTCTTTTGTTTTATTTTACAGAGAAACAAGCTGCAGAACTGGGTGAGTGCTGCCTCCCACTTTTTAA 5001
BG9_GF       GCAGAACTGAGTAAGTCTCCCTCCCAACACGGAAGGGATTTGTGGTCTTCCCATGGGATCAGCCATGGGATGATCATCTGACCC-CTCTCATCATGCATTTCATATTTGTTTCTTTTGCAGAGAAACTGGCTGCAGAACTGGGTGAGTGCTGCCTCCCAAATTAAAT 4627
BG8_GF       GCAGAACTGAGTAAGTCTCCCTCCCAACACGGAAGGGATTTGTGGTCTTCCCATGGGATCAGCCATGGGATGATCATCTGACCC-CTCTCATCATGCATTTCGTATTTGTTTCTTTTGCAGAGAAACTGGCTGCAGAACTGGGTGAGTGCTGCCTCCCAAATTAAAT 4296
BG7_GF       GCAAATCTCAGTATCGCTCCCTATGTACACGGAAGGAATGTGGGGTCTTCCCAT------------GGGATGATAATCTCACTC-TCCTCACAATGAGTTTCTTATTTGTTTCTTTTGCAGAGGAACTGGCTGCAGATCTGGGTGAGTGCTGCCTCCCAAATTAAGA 4209
BG6_GF       GCAGATCTGAGTAAGTCTCCCTACCAGCACGGAAGGAATTTGTGGTCTTCCCATGGGATCAGCCATGGGATGATCATCTGACCC-CTCTCATCATGCATTTCATATTCGTTCCTTTTGCAGAGGAACTGGCTGCAAAATTGGGTGAGTGTTGCCTCCCAAATTAAAT 4545
BG5_GF       GCAAATCTCAGTATCGCTCCCTATGTACACGGAAGGAATGTGGGGTCTTCCCAT------------GGGATGATAATCTCACTC-TCCTCACAATGAGTTTCTTATTTGTTTCTTTTGCAGAGGAACTGGCTGCAGATCTGGGTGAGTGCTGCCTCCCAAACTAAGA 4218
BG4_GF       GCAGATCTGAGTAAGTCTCCCTACCAGCACGGAAGGAATTTGGGGTCTTCCCATGGGATCAGCCATGGGATGATCATCTGACCC-CTCTCATCATGCATTTCGTATTTGTTCCTTTTGCAGAGGAACTGGCTGCAGAACTGGGTGAGTGCTGCCTCCCAAATTAAA- 4456
BG3_GF       GCAGATCTGAGTAAGTCTCCCTCCCGACAATGAAGGAATTTGGGGTCTTCCCATGGGATCAGCCATGGGATGATCATCTGACCC-TTCTCATCATGCATTTTATATTTGATTCCTTTATGCAGAGGAACTGGCTGCAGAACTGGGTGAGTGCTGCCTCCCAAATTAAA- 4529
BG2_GF       GCAATGTTGAGTAAGTCTCATTCCCAAACTGTGGGAATTCAGGATCTCCCCATGGCATTAGCCGGGGATGAGCAGCTATACC-CTCTGACCATGTACTGCTCTGCTCTTCCTTTTCCAGAAAAACTGACTGCAATGTGGGGTAAG------------------ 3990
B12BG1Locus  -----------------------------------GAGATTTGGGGACTTCCCATGGGATCAGCCATGGGATGATAACCTGAGCC-TTCTCATCATGCATGCTTTATTTGATTCTTTATCAGGACACTAGCTGCAGAACTGGGTAAGTCCTCCCTCCCAAACAAAAT 4072
Annotated_BG0 -----------------------------------GGCATTTGGGGTCTTCCCATGGGATCAGCCATGGGATGATAACCTGACCCTTTCTCATCATGCACTTCTTATTTGTTTCCTCTGCAGAGAAACAAGTTGCAGAACTGGGTGAGTCTCCCCTCCCAAATAAAAA 3852

BG13_GF      AAAAAATT--------AGTCTGCCTGTGCGAGCGGTGGGATGAGATGTTCCTCTCACTGTGTATTGCTTTTCCCTTTCTTTTCCAGAGGAAAAATTCTACAGTGATTTGTGAGTCTCCTCTCCCAAATTCTAAATACTGGGGAATTCTTGTATGAGCTGTGGGATGAG 4675
BG12_GF      AAAAAGGG--------GGTCTGCCTGGGAGAGTGGTGGGATGGCACGTTCCTCTCACTGCGTGTTGCTTTT-CCTTTCTTTTCCAGAGAAACACTCTGAAGAGATGGGTGAGTCTCCCCTCCCCAATTATAAATGCTGGGGACTTCTTGTGGGAGCTGTGGGATGAG 4450
BG11_GF      AACAGG----------GGCCTGACTCTGTGAGCAGCGGGATGGGATGTTCCTCTCATCATGTGTTGCTTTTCTCTTCCATTACCAGAGGAACATCTTGCTGAGAAGGGTGAGTCTCCCCTGCCAAATTAAAAACATTGGAGTTTTCCTGTGTGAGCTGTGGGATGAG 4368
BG10_GF      AAAGAGGGTCT-----GGTCTGCCTGTGTGAGCTGTGGGGTGAGGTTTTCTGTTCATCATGTGCTGATTTTCTCTTCCATTTCCAGAGAAACACTTAATAAATACCGGTGAGTCTCCTCTCCCATATTAAAAAGTTGGGGTCTTCCTATGGGAGCTGTGGGATGAG 5163
BG9_GF       AAAAAGGG--------GGTCTGCCTGGGAGAGTGGTGGGATGGCATGTTCCTCTCACTGCGTGTTGCTTTT-CCTTTCTTTTCCAGAGAAACACTCTGAAGAGATGGGTGAGTCTCCCCACCCCAATTATAAATGCTGGGGACTTCTTGTGGGAGCTGTGGGATGAG 4785
BG8_GF       AAAAAATG--------GGTCTGCCTGGGAGAGTGGTGGGATGGCATGTTCCTCTCACTGCGTGTTGCTTTT-CCTTTCTTTTCCAGAGAAACACTCTGAAGAGATGGGTGAGTCTCCCCTCCCCAATTATAAATGCTGGGGACTTCTTGTGGGAGCTGTGGGATGAG 4454
BG7_GF       AACAGG----------GGCCTGACTCTGTGAGCAGTGGGATGGGATGTTCCTCTCATCATGTGTTGCTTTTCTCTTCCATTACCAGAGGAACATCTTGCTGAGAAGGGTGAGTCTCCCCTGCCAAATTAAAAACATTGGAGTTTTCCTGTGTGAGCTGTGGGATGAG 4366
BG6_GF       TAAAAAAG-GG-----GGTCTGCCTGGGCTCGCTGTGGGATAGGATCTTCCTCTCACTGTGTGTTGCTTTTCCCTTTCTTTTCCAGAGGAATATATTGCAGTGAATCGTGAGTCTCCCCTCCGAAATTATAAATGCTGGGGAAATCTTGTGTGAGCTGTGGGATGAG 4706
BG5_GF       AACAGG----------GGCCTGACTCTGTGAGCAGTGGGATGGGATGTTCCTCTCATCATGTGTTGCTTTTCTCTTCCATTACCAGAGGAACATCTTGCTGAGAAGGGTGAGTCTCCCCTGCCAAATTAAAAACATTGGAGTTTTCCTGTGTGAGCTGTGGGATGAG 4375
BG4_GF       TAAAAAAG-GG-----GGTCTGCCTGGGCTCACTGTGGGATAGGATCTTCCTCTCACTGTGTGTTGCTTTTCCCTTTCTTTTCCAGAGGAACACCCTGTCGTGATTTGTGAGTCTCCCCTCTAAAATTATAAATGCTGGGGACTTCTTGTGTGAGCTGTGGGATGAG 4617
BG3_GF       TAAAAAAG-GG-----GGTCTGCCTGGGCTCACTGTGGGATAGGATCTTCCTCTCACTGTGTGTTGCTTTTCCCTTTCTTTTCCAGAGGAACACTCTGAAGAGATGGGTGAGTCTCCCCTCCAAAATTATAAATGCTGGGGACTTCTTGTGTGAGCTGTGGGATGAG 4690
BG2_GF       ------------------------------------------TCTCCCTTCCATTTTTTGTTGAAGTTCTCCCCCTTT---------------------------------------------------------------------------AAATCTGTTGGATGAG 4042
B12BG1Locus  ACAAAA------------------------------------------------------------------------------------------------------------------------------------TGAGGGATGCCTGTGGAGCTGTGGGATGAG 4109
Annotated_BG0 AAAAAAGGGGGGGGAGAGGTCTGCCTGTGTGAGCCATGGGATGAGGTGTTTCTCTCACCATGTGCTGCTTTTTCTCTTTCTTTTCCAGTGGAACAAATTGAAGAATTTGTGAGTCTTCTTCCCTGAACCAAAGGAATATGGGCCTTACTAGGGAAT------GACAAC 4013

BG13_GF      CTCTTCC-TCTCATCATGCGCTG------------TTTCTGCTTTTCCTTTGCAGGGACATGGGATGTAATGTTGAGTGAGTCTCTCTTCCCAAACCAAACAGATCTGGGTGCTTCCCATGGCATCAGCCATGGGATGATAATCGGACCCT-TCTCATTATGCATTT 4828
BG12_GF      CTCTTCC-TCTCATCATGCGCTG------------TTTCTGCTTTTCCTTTGCAGGGACAAGGGATTTAAAGTTGGGTGAGTCTCTCTTCCCAAACCATACAGATTTGGGGTCTTCCCAGCATCAGCCATGGGATGATAATCGGACCCT-TCTCATCATGTGTAT 4603
BG11_GF      ATGTTCC-TCTCATCATGCACTG------------TATCCGCTTTTCCTCTGCAGATTTAAGCACTGCAGATCTGAGTAAGTCTCCCTGCCTACAATGAAGGAATTTGGGGTCTTCCCATGGT-----TGATCCCATGATCATCTAACCCA-TCTCATCATGCATTT 4516
BG10_GF      AAGTTCC-TCTCATCATGCACTG------------TTTCTGCTTTTCCTTTGCAGATTTAAGTGCTGCAGATCTGAGTAAGTCTCCCTCCCTACACTGGAGGAATTTGGCGTCTTCCCATGGG-TCAGTCATGGGATGACAATCTGACCCC-TCTCATCATGCAATT 5315
BG9_GF       CTCTTCC-TCTCATCATGCGCTG------------TTTCTGCTTTTCCTTTGCAGGGACAAGGGATTTAAAGTTGGGTGAGTCTCTCTTCCCAAACCATACAGATTTGGGGTCTTCCCACGGCATCAGCCATGGGATGATAATCGGACCCT-TCTCATCATGTGTAT 4938
BG8_GF       CTCTTCC-TCTCATCATGCGCTG------------TTTCTGCTTTTCCTTTGCAGGGACAAGGGATTTAAAGTTGGGTGAGTCTCTCTTCCCAAACCATACAGATTTGGGGTCTTCCCACGGCATCAGCCATGGGATGATAATCGGACCCT-TCTCATCATGCATTT 4607
BG7_GF       ATGTTCC-TCTCATCATGCACTG------------TTTCCGCTTTTCCTCTGCAGATTTAAGCACTGCAGATCTGAGTAAGTCTCCCTGCCTACAATGAAGGAATTTGGGGTCTTCCCATGGT-----TGATCCCATGATCATCTAACCCA-TCTCATCATGCATTT 4514
BG6_GF       CTCTTCC-TCTCATCATGCACTG------------TTTCTGCTTTTCCTTTGCAGGGAGAAGGAATGTAAAGTTGAGTGAGTCTCTCTTCCCAAACCAAACAGATTTGGGGTCTTCCCATGGGATCAGCCATGGGATGATAATCGGACCCT-TCTCATCATGCATTT 4859
BG5_GF       ATGTTCC-TCTCATCATGCACTG------------TTTCCGCTTTTCCTCTGCAGATTTAAGCACTGCAGATCTGAGTAAGTCTCCCTGCCTACAATGAAGGAATTTGGGGTCTTCCCATGGT-----TGATCCCATGATCATCTAACCCA-TCTCATCATGCATTT 4523
BG4_GF       CTCTTCC-TCTCATCATGCACTG------------TTTCTGATTTTCCTTTGCAGGGACAAGGGATTTAAAGTTGGGTGAGTCTCCCTTCCCAGACAAAACAGATTTGGGGTCTTCCCATGGGATCAGCCATGGGATGATAATCGGACCAT-TCTCATCATCCATTT 4770
BG3_GF       CTCTTCC-TCTCATCATGCACTG------------TTTCTGATTTTCCTTTGCAGGGACATGGAGTGTACCGTTGAGTGAGTCTCTCTTCCCAAACCAAACAGATTTGGGGTCTTCCCATGGGATCAGCCATCGGATGATAATCGGACCCT-TCTCATCATGCGTTT 4843
BG2_GF       ATGTTCC-TTTCCTCATGCACTA------------TTTCTGCTTTTCCTCATTGTAGATTTAAGTGCTGCAAATCTGAGTAAGTCTCCCTGCCTACACTGAAGGATTTGGGGCTTTCCCATGGGATCGGCCATGGGATGATAATCCGACCCT-TCCCATCATGCATTT 4195
B12BG1Locus  CTGTTCA-GTTCATCACGCACTG------------CTTCTGCTTTTCCTTTGCAGAGAGAAGGGATGCAAAGTTGGGTGAGTC---CTCCGCAAACCAAAGAGATTCAAGGTCTTCCCATGGGATCAGCCATGGGATGATATTCTGACCCT-TCTCATCACGCATTT 4259
Annotated_BG0 ATGTCCATCTCATCATGTGTGATTTTTCTTTTTTTTTTTTTCTTTCTTTGCAGAGAAAGAAATCACAATTGAGTAAGTTACATTCACTAAACTGAGGGGAATTTGGGGTCTTCCCATGGGATCAGCCATGGGATGATAACCTGACCCTTTCTCATCATGCACTT 4180

BG13_GF      CTTATTGGTTCCTTTT----GCAGAGGTACTAGCTGCCAAACTGGGTGAGT-CCCCCCTCCCAAATT--ACATAAAAAATGGG----GTCATCCTGTGTGAGCTGTGGGATGAG-ATGTTCCTCTCATCATGCGCTGCTTTTCTCTTCCTTTTCCAGGACACAAAG 4982
BG12_GF      CTTATTGGTTCCTTTT----GCAGAGCGACTAGCTGCCAAACTGGGTGAGT-CCCCCCTCCCAAATT--AAATAAAAAATGGG----GTCTGCCTGTGTGAGCTGTGGGATGAG-ATGTTCCTCTCATCATGCGCTGCTTTTCTCTTCCTTTTCCAGAACATCAAA 4757
BG11_GF      CTCATTCGTTCCTTTT----GCAGAGCTACTAGCTGCAAAACTGGGTGAGT-CCTCCCTCCCAAATTAAATAAAAAAAA-GGG----GTCTGCCTGTGTGAGCTGTGGGATGAG-ATGTTCCTCTCATCATGTGTTGCTTTTCTCTTTCTTTTCCAGTGGAACAAA 4671
BG10_GF      CTTTTTTTTTTTTTTTTTGTAGACATAGCAGCTGCAAAACTGGGTGAGT-GCCCCCTCCTAAATTAAAAACAG-----GGT-----GTCTACCTTGTGAGCTGTGGTATGAG-ACGTTCCTCTCATCAGGCATTGCTTTTCACTTCCTTTTCCAGACAAACAAA 5470
BG9_GF       CTTATTGGTTCCTTTT----GCAGAGCGACTAGCTGCCAAACTGGGTGAGT-CCCCCCTCCCAAATT--ACATAAAAAATGGG----GTCTGCCTGTGTGAGCTGTGGGATGAG-ATGTTCCTCTCATCATACGCTGCTTTTCTCTTCCTTTTCCAGAACATCAAA 5092
BG8_GF       CTTATTGGTTCCTTTT----GCAGAGCGACTAGCTGCCAAACTGGGTGAGT-CCCCCCTCCCAAATT--AAATAAAAAATGGG----GTCTGCCTGTGTGAGCTGTGAGATGAG-ATGTTCCTCTCATCATGCGCTGCTTTTCTCTTCCTTTTCCAGAACATCAAA 4761
BG7_GF       CTCATTGGTTCCTTTT----GCAGAGCTACTAGCTGCAAAACTGGGTGAGT-CCTCCCTCCCAAATTAAATAAAAAAAA-GGG----GTCTGCCTGTGTGAGCTGTGGGATGAG-ATGTTCCTCTCATCATGTGTTGCTTTTCTCTTTCTTTTCCAGTGGAACAAA 4669
BG6_GF       CTTATTGGTTCCTTTT----GCAGATAATATAGCTGCCAAACTGGGTGAGT-CCCCCCTCACAGATT--ACATAAAAAATGGG----GTCTGCCTGTGTGAGCTGTGGGATGAG-ATGTTCCTCTCATCATGTACTACTTTTCTCTTCCTTTTCCAGCACAACAAA 5013
BG5_GF       CTCATTGGTTCCTTTT----GCAGAGCTACTAGCTGCAAAACTGGGTGAGT-CCTCCCTCCCAAATTAAATAAAAAAAA-GGG----GTCTGCCTGTGTGAGCTGTGGGATGAG-ATGTTCCTCTCATCATGTGTTGCTTTTCTCTTTCTTTTCCAGTGGAACAAA 4678
BG4_GF       CCCATTGGTTCCTCT-----GCAGAGCGAATAGCTGCCAAACTGGGTGAGT-CCCCCCTCCCAAATT--AAATAAAAAATGGG----GTCTG-CTGTGTGAGCTGTGGGATGAG-ATGTTCCTCTCATCATGCGCAGCTTTTCTCTTCCTTTTCCAGCACAACAAA 4923
BG3_GF       CTTATTCGTTCCTTTT----GCAGAGCTACTAGCTGCCAAACTGGGTGAGTCCCCCCCTCCCAAATT--AAATAAAAAATGGG----GTCTGCCTGTGTGAGCTGTGGGATGAG-ATGTTCCTCTCGTCATTTGCTGCTTTTCCCTTCCTTTTCCAGGACGACAAA 4998
BG2_GF       CTCATTTGTTTCTTTT----GCAGAAGCACAGGCTACCAAACTGGGTGAGT-CCTCCCTCACAAATTAGAAACAATAAAGGG----CTCTGCCTGTGTGAGCTGTGGGATGAGTGCATGATGCTCATCATGCACTGCTTTCTCTTTTCTTCTTTTCCAGTGGAAAGAA 4352
B12BG1Locus  CTTATTTGATTCTTTT----GCAGAGCACTAGTTGAAAATCTGGGTGAGT-GCTGCCTTAAAATTAAACAAAAAA---GGG----GTTTTCCTTTGGGAGCTGTGGGACGAG-ATGCTCCTGTCTTCATGCATTGTTTCTGCCTTTCTTTTGTAGAGAGAAGAA 4412
Annotated_BG0 CTTATTTGTTTCCTCT----GCAGAGAAACAAGTTGCAGAACTGGGTGAGT-CTCCCATCCCAAATAAAAAAAAAGGGGGGGGGGAGGTCTGCCTGTGTGAGCCATGGGATGAG-ATGTTCTCTCACCACGTGCTGCTTTTCTCTTTCTTTTCCAGTGGAACAAA 4341

BG13_GF      CTAAAGAATTGGGTGAGTCTTCTTTCCCCAA-CCAAAGAAATAT-GGGTTTCCCATGGGATGACAAGCTGTGCCACCTCATCATGCCCTGTTTCTTCTGTTCTT-----------TTTGCAGAGAAACAGCATTTACAGTTCTGTAAGTTGCAGCCACTGAACTGAA 5136
BG12_GF      CTAAAGAATTGGGTGAGTCTTCTTTCCCCAACCCCAAGAAATAT-GCGTTTCCCATGGGATGACAAGCTGTGCCACCTCATCATGCCCTGTTTTTTCTGTTCTT-----------TTTGCAGAGAAACAGCATTCACAGTTCCGTAAGTTGCAGTCACTGAACTGAA 4912
BG11_GF      GAGAAGCAGTGGGTGAGTCTTCTTCCCCTAGA-CCCAGGAACATGGGATGTTCAATGGGATGGCAAGCTGTCCCAATTCATCATGTGTTACTTTTCCTTT---------------TTGGCAGAGGAATGGGATTCACAGCTGAGTAAGTTGCAGTCACTAAACTGAG 4822
BG10_GF      CTGAAGAACTGGGTGAGTCTTCTTCCCAGAA-CCAAATGAATTTGGGGTTTCCAATGAGATGACAAGCTTACCATCTCACTATTCATTGTTTTCCTCTTCTTT----------TTGGCAGACAAATGGAAATCAGCACTGAGTAAGTTGTAATCAATAAACTGAG 5626
BG9_GF       CTAAAGAATTGGGTGAGTCTTCTTTCCCCAACCCCAAGAAATAT-GCGTTTCCCATGGGATGACAAGCTGTGCCACCTCATCATGCCCTGTTTTTTCTGTCCTT-----------TTTGCAGAGAAACAGCATTCACAGTTCCGTAAGTTGCAGTCACTGAACTGAA 5247
BG8_GF       CTAAAGAATTGGGTGAGTCTTCTTTCCCCAACCCCAAGAAATAT-GCGTTTCCCATGGGATGACAAGCTGTGCCACCTCATCATGCCCTGTTTTTTCTGTCCTT-----------TTTGCAGAGAAACAGCATTCACAGTTCCGTAAGTTGCAGTCACTGAACTGAA 4916
BG7_GF       GAGAAGCAGTGGGTGAGTCTTCTTCCCCTAGA-CCCAAGGAACATGGGATGTTCAATGGGATGGCAAGCTGTCCCAATTCATCATGTGTTACTTTTCCTTT---------------TTGGCAGAGGAATGGGATTCACAGCTGAGTAAGTTGCAGTCACTAAACTGAG 4820
BG6_GF       CTAAAGAATTGGGTGAGTCTTCTTTCCCCAA-ACAAAGAAATAC-GGGTTTCCCATGGGATGACAAGCTGTGCCACCTCATCATGCCCTGTTTTTTCTGTCCTT-----------TTTGCAGAGAAACAGCATTTCAGCGTATGGGTAAGTTGCAGTCACTAAACTGAA 5167
BG5_GF       GAGAAGCAGTGGGTGAGTCTTCTTCCCCTAGA-CCCAAGGAACATGGGATGTTCAATGGGATGGCAAGCTGTCCCAATTCATCATGTGTTACTTTTCCTTT---------------TTGGCAGAGGAATGGGATTCACAGCTGAGTAAGTTGCAGTCACTAAACTGAG 4829
BG4_GF       CTAAAGAATTGGGTGAGTCTTCTTTCCCCAA-ACAAAGAAATAC-GGGTTTCCCATGGGATGACAAGCTGTGCCACCTCATCATGCCCTGTTTTTTCTGTCCTT-----------TTTGCAGAGAAACAGCATTCACAGTTCTGTAAGTTGCAGCCACTAAACTGCA 5077
BG3_GF       CTAAAGAATTGGGTGAGTCTTCTTTCCCCAA-CCAAAGAAATAC-GGGTTTCCCATGGGATGACAAGCTGTGCCACCTCATCATGCCCTGTTTTTTCTGTCCTT-----------TTTGCAGAGAAACAGCATTCACAGTTCTGTAAGTTGCAGCCACTAAACTGAA 5152
BG2_GF       CTGGAGAATTGGGTGAGCGTCTTCCCCAAA-CTCAAGGAATGTGGGGTTTCCCATGGGATGACAAGCTGTCCCACCTCATCATGCGGTGCTTTTTCTTTTTTTCCTTATTTGCTTTGCAGAGATACGGAATTCACTCTTGAGTAAGTTGCAGACAGAAACTGAG 4518
B12BG1Locus  ACACAGAGTTCGGTGAGTCTTCCTCCGCTAA-CCAAAGAGATCTGGGGTATTC-------------------------------------------------------------------------------------------------------------------- 4464
Annotated_BG0 TTGAAGAATTTGGTGAGTCTTCTTCCCTGAA-CCAAAGGAATATGGGCCTTACCAGGGAATG--------TGTTGATTTTCCTTTTTTTTTTTTTTCTTTCTTTC--------TTTGCAGAGAAAGAGAATTCACAATTGAGTAAGTTGCATTCACTAAACTGAG 4490

BG13_GF      GGAATGTGGGGTTTTCCCAAAGTCCTGCATATGAGATGAGAAATCCCCTCTGACCATGAACTGCTTTTCTCTTCTTTTATTCCAGACAGACACTTTCAGCATATGTGTGAGTCTCTCCCCCCCAAAT---TAAAAACATTGGGGTCTTCCTGTATGAGCTGTGGGATGA 5300
BG12_GF      GGAATGTGGGGTCTTCCCAAAGTCCTGCATGTGGGATGAAAAATCCCCTCTGACCATGCACTGCTTTTTCTCTTCTATTCCAGAGAGACACTTTCAGAATATGTGTGAGTCTCCCAACCCCTGATAAATAAAAACGTTGGGGTCTTGCTGTGTGAGCTGTGGGATGA 5079
BG11_GF      GGAATCTGGGGTCATCCCAAGGACTGCACATGGGATGAAAAATCCCCTCCACCATGCACTCCTTTTTCTCTTTCTTTTTCCAGGGAAACAGTACGAAAAGTTGGGTGAGTCTCCATTTCCATACA---AAAAAACGTTGGGGTCTTGCTTTGTGAGCTGGGGATGA 4986
BG10_GF      AGAATATGGGGCTCTCCCAAGCGATTGTGTATGGGATGAAATATCCCCTCTGACCATGCACTGCTTT--TCTTTCTTTTTCCAGAGATACAATATGAAAAGTTGGGTGAGTCTCCA-ACCTATCAAAA---TAAAAACACTGGGATCTTCCTGCGGAGCTGTGGGATGA 5787
BG9_GF       GGAATGTGGGGTCTTCCCAAAGTCCTGCATGTGGGATGAAAAATCCCCTCTGACCATGCACTGCTTTTTCTCCTTCTATTCCAGAGAGACACTTTCAGAATATGTGTGAGTCTCCCCACCCCTGATAAATAAAAACGTTGGGGTCTTGCTGTGTGAGCTGTGGGATGA 5414
BG8_GF       GGAATGTGGGGTCTTCCCAAAGTCCTGCATGTGGGATGAAAAATCCCCTCTGACCATGCACTGCTTTTTCTCCTTCTATTCCAGAGAGACACTTTCAGAATATGTGTGAGTCTCCCCACCCCTGATAAATAAAAACGTTGGGGTCTTGCTGTGTGAGCTGTGGGATGA 5083
BG7_GF       GGAATCTGGGGTCATCCCAAGGACTGCACATGGGATGAAAAATCCCCTCCAACCATGCACTCCTTTTTCTCTTTCTTTTTCCAGGGAAACAGTACGAAAAGTTGGGTGAGTCTCCATTTCCATACA---AAAAAACGTTGGGGTCTTGCTTTGTGAGCTGGGGATGA 4984
BG6_GF       GGAATGTGGGGTCTTCCCAAAGTCCTGCATACGGGATGAAAAATCCCCTCTGACCATGCACTGCTTTTTCTCTTCTATTCCAGACAGACACTTTCAGCGTATGGGTGAGTCTCTCCCCCCCAAAT---TAAAAACGCTGGGGTCTTCCTATGGGAGCTGTGGGATGA 5331
BG5_GF       GGAATCTGGGGTCATCCCAAGGACTGCACACGGGATGAAAAATCCCCTCCGACCATGCACTCCTTTTTCTCTTTCTTTTTCCAGGGAAACAGTATGAAAAGTTGGGTGAGTCTCCATTTCCATACA---AAAAAACGTTGGGGTCTTGCTTTGTGAGCTGGGGATGA 4993
BG4_GF       GGAATGTGGGGTCTTCCCAAAGTCCTGCATATGGGATGAAAAATCCCCTCTGACCATGCACTGCTTTTTCTCTTCTATTCCAGACAGACACTTCCAGCATATGGGTGAGTCTC-CCCCCCAAAT---TAAAAACATTCGTGTCTTCCTATGGGAGCTGTGGGATGA 5240
BG3_GF       GGAATGTGGGGTCTTCCCAAAGTCCTGCATGTGGGATGAAAAATCCCCTCTGACCATGCACTGCTTTTTCTCTTCTATTCCAGACAGACACTTCCAGCATATGGGTGAGTCTC-CCCCCC-AAAT---TAAAAACATTCGTGTCTTCCTATGGGAGCTGTGGGATGA 5314
BG2_GF       GGAATTTGGGGTCTTCCCAAGGGACTGTGTATGGGATGAAAAATCCCCTCTGACCTTGCACTGCTTTTTCTCTTTCTTTTTACAGAGAGACATTTAAGAAAGATGGGTGAGTCTC-CACTCCTAAAT---TAAAATCTTTGGGGTCTTCCGGTGTGAGCTGCGGGATGA 4681
B12BG1Locus  ----------------------------------------------------------------------------------------------------------------------------------------------------------------------------- 4464
Annotated_BG0 GGAATTTGGGGTCTTTCCAAGGGATTACTTATGGGACAAAATATCCCCTCTGACCATGCACTGCTTTTTCTCTTTCTTTTTCCAGAGGAACATTATATGAAGATGGGTGAGTCTC-TCCTCCCAAAT---TAAAAACGCTGGGGTTTTCTTGTGGAGCTGTGGGATGA 4653
```

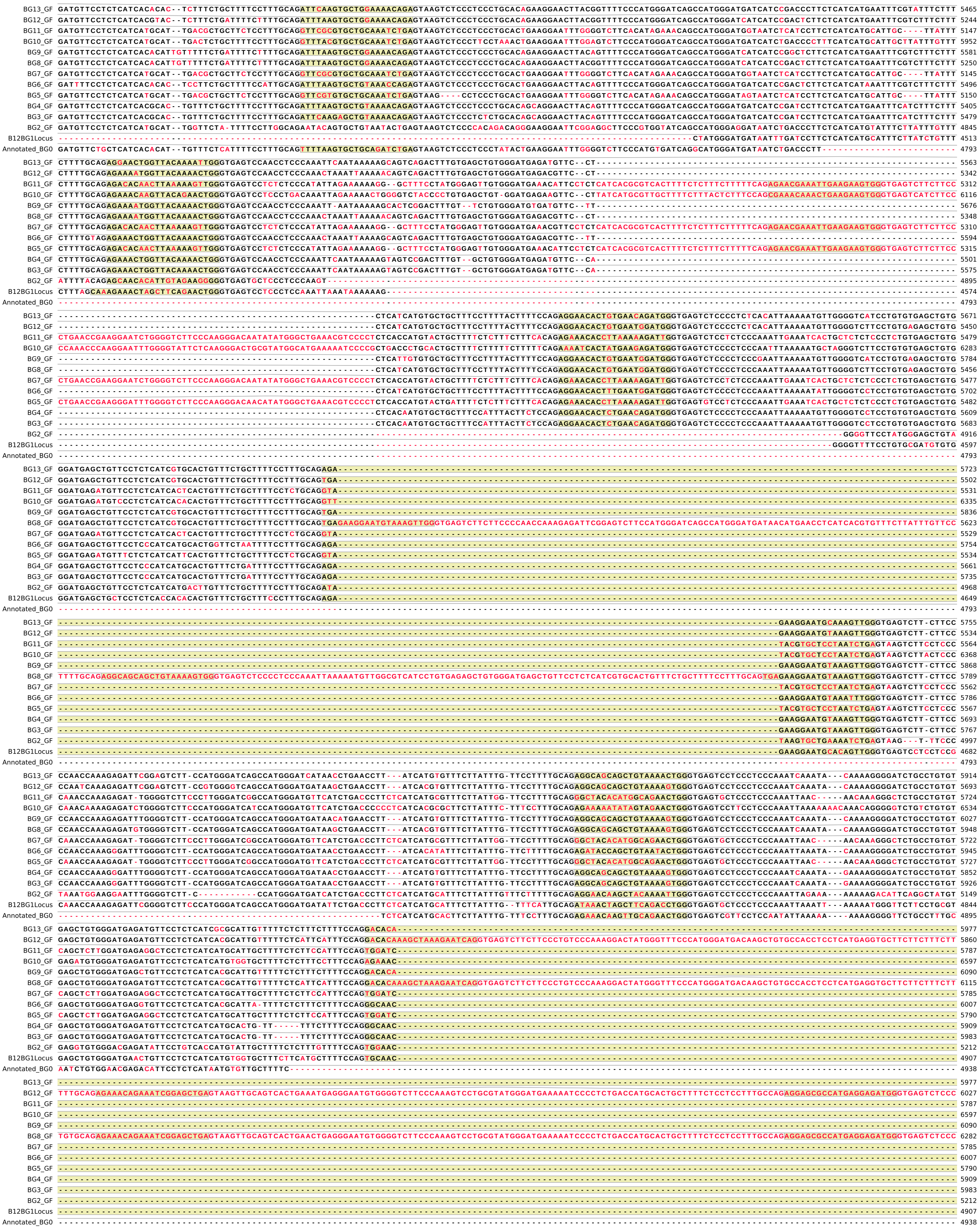
BG13_GF
BG12_GF
BG11_GF
BG10_GF
BG9_GF
BG8_GF
BG7_GF
BG6_GF
BG5_GF
BG4_GF
BG3_GF
BG2_GF
B12BG1Locus
Annotated_BG0

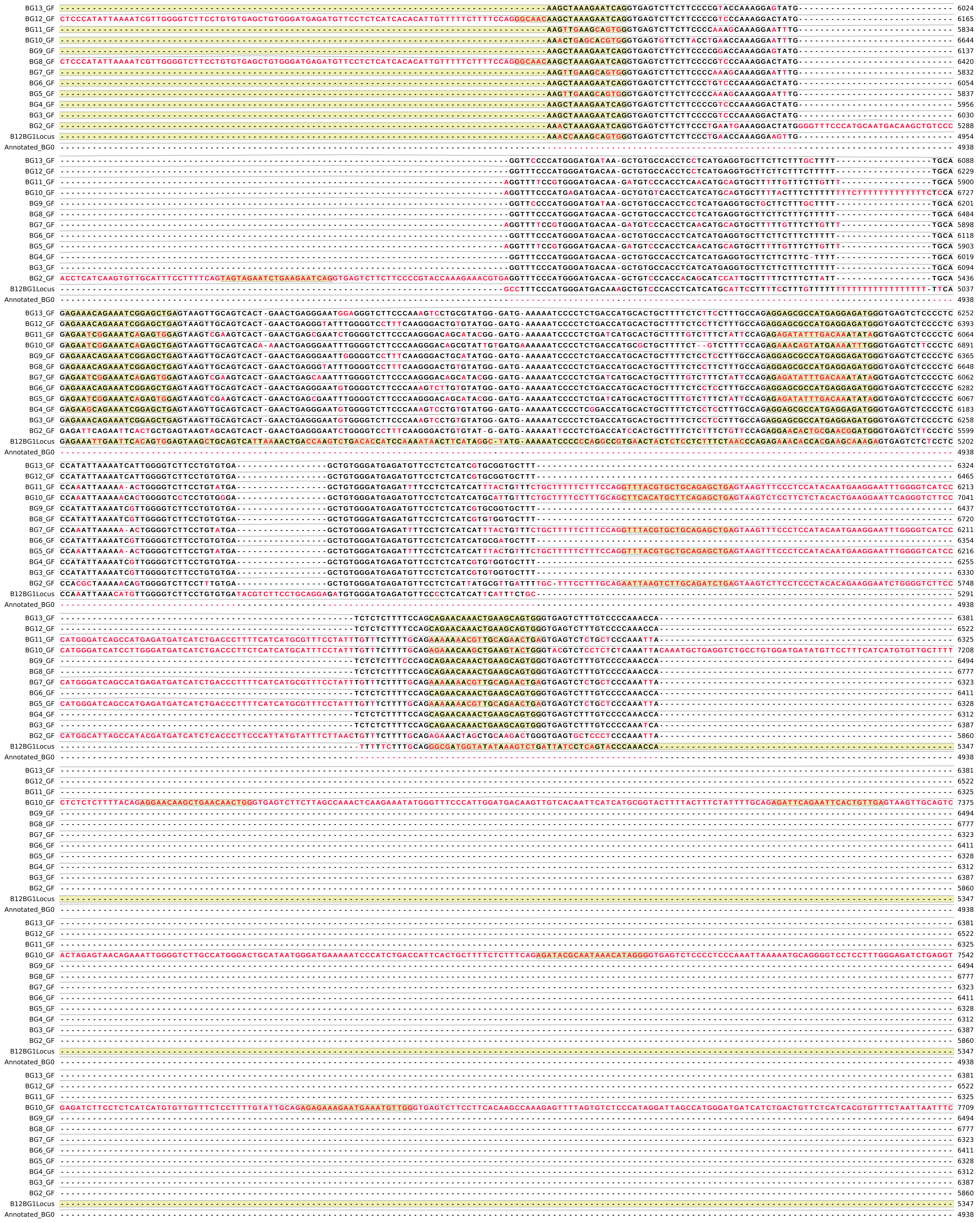

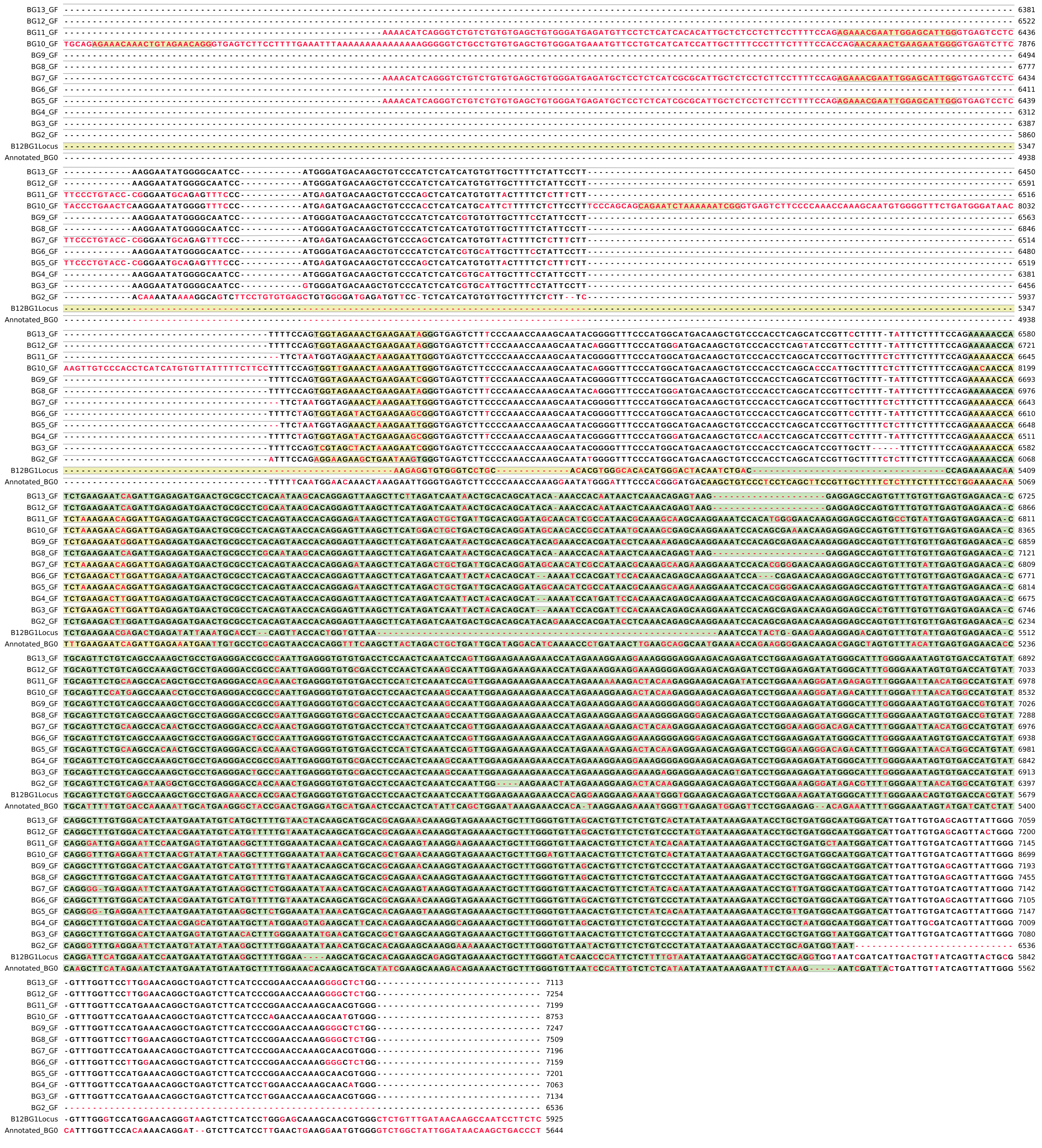

Supplement: Figure S2 — Alignment of the genomic sequence from all of the B12 BG genes, showing the locations of the coding sequence (yellow background), UTRs (green background) and the introns/intergenic sequence (white background). Residues which differ from a majority consensus sequence (not shown) are red; exons are highlighted in green; dashes are gaps included to maximise alignment. Each Genomic Fragment (GF) was cut 76 nucleotides after the predicted end of the 3'UTR, with the start of the 5' end just after the breakpoint of the previous 3' end (except for BG 10, preceded by the hypothetical protein, which was trimmed to match the 5' ends of the other genes). All features of the BG genes were initially predicted by reference to known BG cDNAs (as shown in Figure S5), and most have been validated by comparison to cDNAs from the particular gene. While not shown in this figure, the features of the C-type lectin-like, kinesin motor protein and hypothetical genes were established by reference to ESTs which span at least two exons and are over 90% identical to the genomic sequence (lectin: CD216101,CN217694; kinesin: BU471750, BU333462, BU251676, BU112055, BU108596, AM068009, AM063157, CV892843, CD217632, BU487705, BU330950, AJ442873, AJ398446, AJ398368, DT659179, DT655924, DR419449, CO507363, CN232628, CD217592, BU386334, BU354226, BM491516, BM490485, BM489471, BM425939, BG625424, AJ453838, AJ444817, DT657232, CD738123, CD728354, CD215461, BU199828, BU108016, BI393544, BI391565, AJ452709, AJ398223, AJ393429, BU328290, AJ399432, AJ393414, AJ393212 and the hypothetical protein: BU207055, BU209867, CN232293, BU374683, AJ442408, CB017432, AJ445065, AJ399199, BU451417, BU299883, BU357505, BU333629. (PDF) [file pgen.1004417.s002.pdf]
