# Supplementary material for: Sequence of a Complete Chicken BG Haplotype Shows Dynamic Expansion and Contraction of Two Gene Lineages with Particular Expression Patterns
Source: PLoS Genet. 2014 Jun 5;10(6):e1004417. doi: 10.1371/journal.pgen.1004417 (PMC4046983; doi:10.1371/journal.pgen.1004417)
Supplement: Figure S8 — There is only one 3′UTR exon for the BG0 gene in B21 chickens (line N). A. Dot plot analysis of the sequence around the 3′UTR from the WGS sequence shows duplication including the 3′UTR, with an insertion in the second copy. Three pairs of primers were designed just outside the apparent duplication and used for PCR from genomic DNA from a line N chicken, with the sizes expected for amplicons from the region with and without a duplication indicated below the dot plot. B. Picture of the amplification products separated by agarose gel electrophoresis, showing major amplified band below 3 kB compared to markers, as expected if the duplication is not found in the genome. C. Alignment between the WGS sequence 2.1 and the end sequences from the 3 kb band from PCR1. (PDF) [file pgen.1004417.s008.pdf]

A

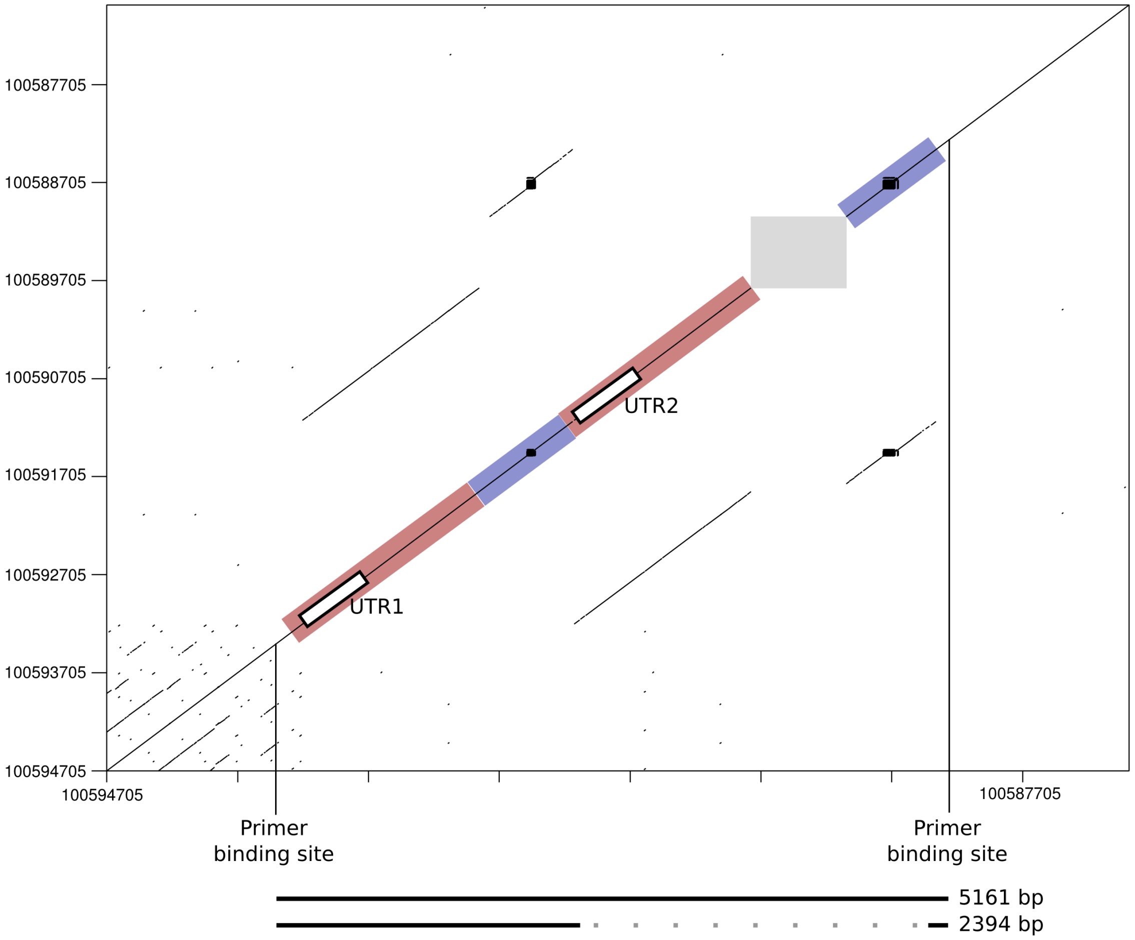

100587705
100588705
100589705
100590705
100591705
100592705
100593705
100594705
UTR2
UTR1
100594705
100587705
Primer binding site
Primer binding site
5161 bp
2394 bp

B

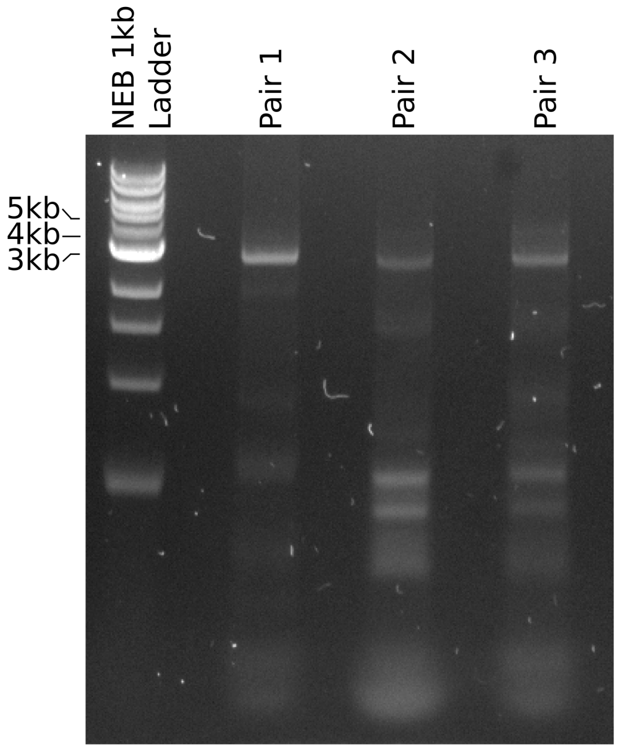

NEB 1kb Ladder
Pair 1
Pair 2
Pair 3
5kb
4kb
3kb

C

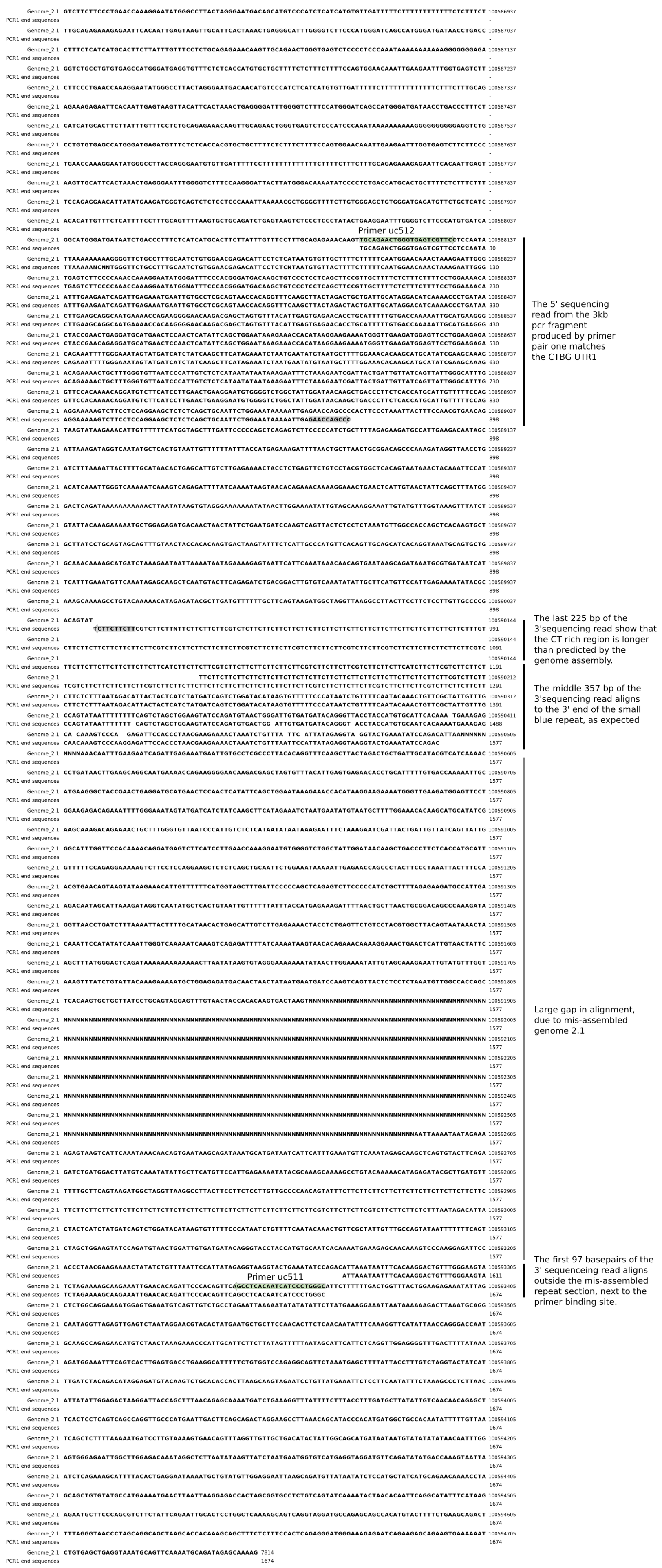
Genome_2.1
PCR1 end sequences
Primer uc512
The 5' sequencing read from the 3kb pcr fragment produced by primer pair one matches the CTBG UTR1
The last 225 bp of the 3'sequencing read show that the CT rich region is longer than predicted by the genome assembly.
The middle 357 bp of the 3'sequencing read aligns to the 3' end of the small blue repeat, as expected
Large gap in alignment, due to mis-assembled genome 2.1
The first 97 basepairs of the 3' sequenceing read aligns outside the mis-assembled repeat section, next to the primer binding site.
Primer uc511
